# Interfacial Bonding between a Crystalline Metal–Organic Framework and an Inorganic Glass

**DOI:** 10.1021/jacs.3c04248

**Published:** 2023-10-11

**Authors:** Celia Castillo-Blas, Ashleigh M. Chester, Ronan P. Cosquer, Adam F. Sapnik, Lucia Corti, Roman Sajzew, Bruno Poletto-Rodrigues, Georgina P. Robertson, Daniel J.M. Irving, Lauren N. McHugh, Lothar Wondraczek, Frédéric Blanc, David A. Keen, Thomas D. Bennett

**Affiliations:** † Department of Materials Science and Metallurgy, University of Cambridge, Cambridge CB3 0FS, U.K.; ‡ Department of Chemistry, University of Liverpool, Crown Street, Liverpool L69 7ZD, U.K.; § Leverhulme Research Centre for Functional Materials Design, Materials Innovation Factory, University of Liverpool, Liverpool L7 3NY, U.K.; ∥ Otto Schott Institute of Materials Research, University of Jena, Fraunhoferstrasse 6, 07743 Jena, Germany; ⊥ Diamond Light Source Ltd., Diamond House, Harwell Campus, Didcot, Oxfordshire OX11 0QX, U.K.; # Stephenson Institute for Renewable Energy, University of Liverpool, Crown Street, Liverpool L69 7ZF, U.K.; ∇ ISIS Facility, Rutherford Appleton Laboratory, Harwell Campus, Didcot, Oxfordshire OX11 0QX, U.K.

## Abstract

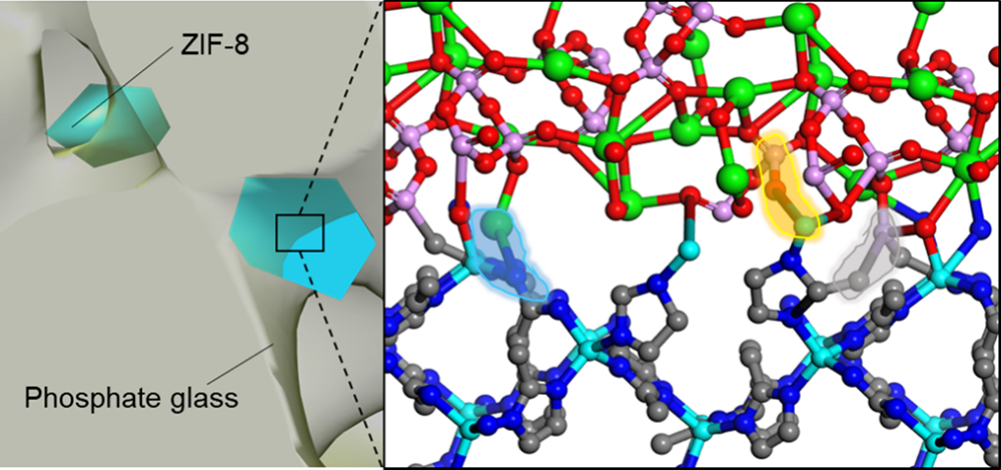

The interface within a composite is critically important for the chemical and physical properties of these materials. However, experimental structural studies of the interfacial regions within metal–organic framework (MOF) composites are extremely challenging. Here, we provide the first example of a new MOF composite family, i.e., using an inorganic glass matrix host in place of the commonly used organic polymers. Crucially, we also decipher atom–atom interactions at the interface. In particular, we dispersed a zeolitic imidazolate framework (ZIF-8) within a phosphate glass matrix and identified interactions at the interface using several different analysis methods of pair distribution function and multinuclear multidimensional magic angle spinning nuclear magnetic resonance spectroscopy. These demonstrated glass–ZIF atom–atom correlations. Additionally, carbon dioxide uptake and stability tests were also performed to check the increment of the surface area and the stability and durability of the material in different media. This opens up possibilities for creating new composites that include the intrinsic chemical properties of the constituent MOFs and inorganic glasses.

## Introduction

The metal–organic framework (MOF) field has attracted great interest in recent years, thanks to their exciting properties suitable for many applications such as heterogeneous catalysis, drug delivery, or gas storage.^1−3^ MOFs are a class of hybrid materials formed by the self-assembly of an organic linker and an inorganic metal cluster, culminating in open 3D architectures with large surface areas and pores.^4^ However, their microcrystalline powder form presents difficulties for their industrial implementation, due to weak mechanical performance.^5^ To address these issues, the preparation of MOF composites, i.e., MOFs embedded within a more easily processable material matrix such as graphene oxide or polymers, has gained increasing attention.^6^

Recently, glasses formed by melt-quenching zeolitic imidazolate frameworks (ZIFs) have offered promising mechanical properties.^7^ ZIFs are a subgroup of highly thermally stable MOFs, composed of metal cations tetrahedrally coordinated through imidazolate linkers.^8^ The use of ZIF glasses as matrix materials within MOF composites can, for example, stabilize a metastable phase of MIL-53 at room temperature.^9^ However, examples of melt-quenched ZIF glasses remain relatively rare and therefore their use as composite matrices is not widely applicable.^10^

In contrast, inorganic glasses are a broad family of materials with a vast number of applications, ranging from commodities such as architectural or container glasses to speciality glasses widely employed in biotechnology, photonics, nuclear waste management, or solid-state electrolytes.^11^ In general, inorganic glasses avoid crystallization either by having complex chemical compositions, also called “principle of maximum confusion”,^12^ or by kinetically frustrating the crystallization process by cooling the melt beyond its critical cooling rate.^13^ They are relatively cheap and well-studied materials, though they are almost completely unexplored as host materials for crystalline MOFs.^14,15^ No example of successful crystalline MOF–inorganic glass composite formation is known to the authors at the time of writing. Exploring this class of composites, together with the need to improve MOF mechanical properties and reduce their costs, is the motivation for this work.

The enormous number of available inorganic glasses means that the number of possible composite materials is vast, especially when considered relative to those using pure MOF–glass matrices. Phosphate-based glasses in particular are broadly studied thanks to their relatively low melting temperatures and biocompatibility.^16−18^ Additionally, the glass structure and several of its properties, such as the glass transition temperature (*T*_g_), melt fragility, dissolution kinetics, surface hardness, or elastic moduli, can be tailored by adjusting its chemical composition.^19−21^

Motivated by the untapped potential of inorganic glasses as matrices for crystalline MOFs, we fabricated and characterized a new family of composites formed by ZIF-8 and an inorganic glass (IG), 50(Na_2_O)-50(P_2_O_5_). We will refer to these materials generally as “metal–organic framework crystalline-inorganic glass composites (MOF-CIGCs)”. We show several approaches to analyzing pair distribution function (PDF) data, which reveal atomic insight into interface interactions, and we show that these further demonstrated from multinuclear multidimensional magic angle spinning (MAS) nuclear magnetic resonance (NMR) data.

**Figure 1:**
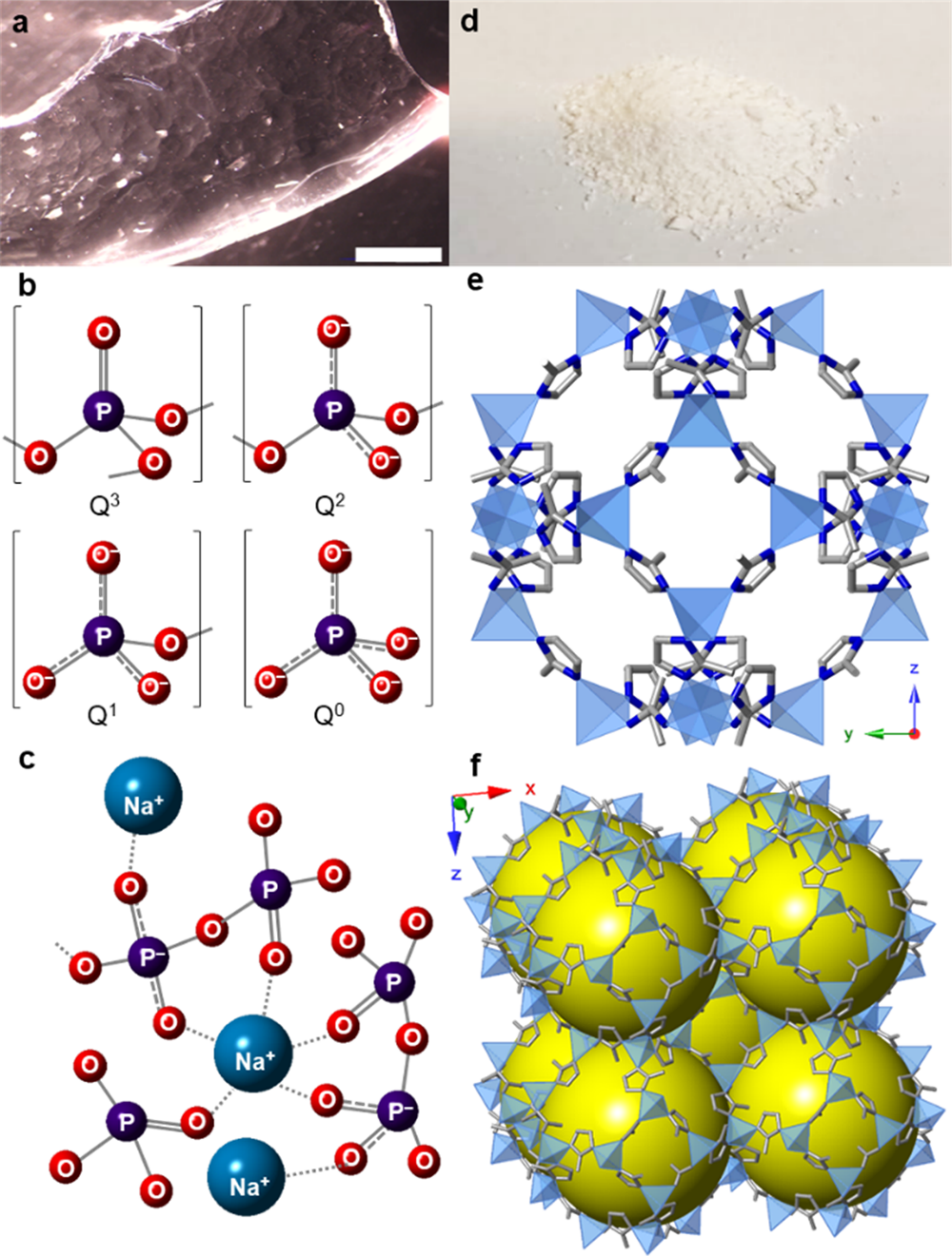
(a) Optical image of a 50(Na_2_O)-50(P_2_O_5_) glass piece (scale bar, 500 μm). (b) Depictions of the PO_4_ tetrahedral units that build phosphate glass structures. *Q*^3^, *Q*^2^, *Q*^1^, and *Q*^0^ correspond to phosphorus pentoxide, meta-, pyro-, and ortho-phosphate, respectively. (c) Schematic depiction of a sodium-phosphate glass structure. (d) Optical image of pristine ZIF-8. (e) ZIF-8 unit cell. Zn tetrahedra are shown in blue, and C and N are depicted as sticks in gray and dark blue, respectively. (f) Schematic depiction of the ZIF-8 structure, showing the pores as yellow spheres.

50(Na_2_O)-50(P_2_O_5_) inorganic glass was chosen for its greater durability and stability in air compared to other sodium phosphate-based glasses (Figure 1a–c). These phosphate glasses are composed of PO_4_ tetrahedral units and described using the *Q^n^* designation (where n denotes the number of bridging oxygens on a given phosphate tetrahedral unit).^22^ ZIF-8, Zn(mIm)_2_ (mIm = 2-methylimidazolate, C_4_H_5_N_2_^–^) was selected as it is the ‘prototypical’ crystalline ZIF, whose properties and structure have been extensively studied (Figure 1) and which is also commercially available under the name Basolite Z1200.^23^

## Results and Discussion

### Optimisation of the Synthesis of the MOF-CIGCs

**Figure 2:**
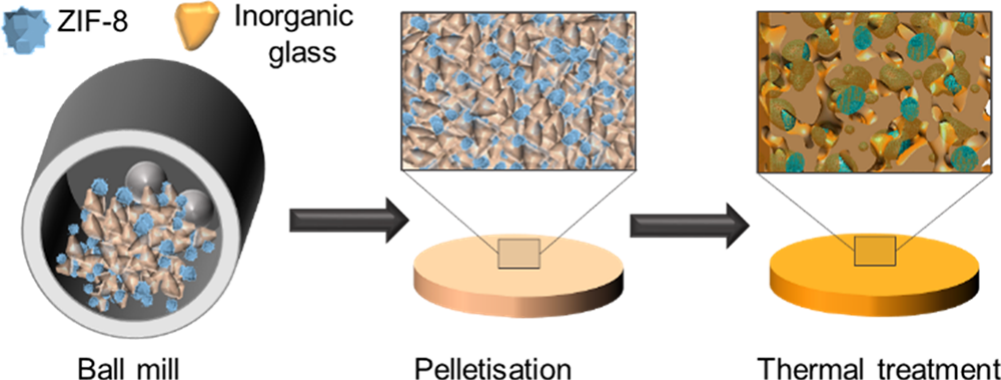
Schematic representation of the synthetic procedure of MOF-CIGC materials where, first, ZIF-8 (blue particles), and the selected inorganic glass (orange particles) were ball milled together. After this, the physical mixture was pelletized at an optimized pressure followed by a thermal treatment at the chosen working temperature.

The synthetic procedure involves ball milling both ZIF-8 and IG together to prepare a physical mixture followed by pelletization and heat treatment at a specific dwell temperature. This is denoted as the working temperature (*T*_w_) to yield a composite material (Figure 2).

ZIF-8 and IG were synthesized according to published reports (see Supporting Information).^24,25^ ZIF-8 was characterized through powder X-ray diffraction (PXRD) to determine the phase purity and thermogravimetric analysis (TGA) to obtain the decomposition temperature (*T*_d_). This is the maximum value of the working temperature (*T*_w_) in the preparation of the composite (Figures S1 and S2). Scanning electron microscopy (SEM) images were collected to analyze the size and morphology of the ZIF crystals (Figure S3). The inorganic glass was characterized by PXRD (Figure S4) and SEM-energy dispersive spectroscopy (SEM-EDS). This confirmed the lack of crystallinity in the sample and the phosphorus and sodium contents, respectively (Table S1). SEM images were used to analyze the size and morphology of the glass (Figure S5). Differential scanning calorimetry (DSC) showed a glass transition temperature (*T*_g_) value of 300 °C, coincident with the literature value (Figures S6 and S7).^26^*T*_g_ is the temperature where the reversible transition between glassy and viscoelastic behavior in glasses occurs. Additionally, the DSC of the material also contains an exothermic peak, at ca. 350 °C. This is ascribed to the temperature of glass recrystallization (*T*_r_), in this case to a known crystalline Na_3_P_3_O_9_ structure (Figure S8).^27^

A physical mixture with a weight percentage incorporation of 20% ZIF-8 and 80% IG, termed (ZIF-8)_0.2_/(IG)_0.8_, was employed to determine the optimal conditions for the preparation of the physical mixtures ((ZIF-8)_x_/(IG)_1-x_) and MOF-CIGC materials [(ZIF-8)_x_(IG)_1-x_], where x is the proportion by weight of the ZIF-8 compound in the composite (see Supporting Information). To facilitate the homogenization of the physical mixture, the IG was initially ball-milled for 30 min at 30 Hz to obtain a particle size of ∼5 μm (Figure S9). However, the glass was observed to adsorb water on its surface due to the hygroscopic nature of these phosphate-based glasses. These water molecules are able to disrupt the glass network upon heating and, consequently, decrease both *T*_g_ and *T*_r_ values of the glass from 297 and 362 °C to 250 and 312 °C, respectively (Figures S10 and S11).

For successful composite formation, both the selected glass and the MOF material should be thermally compatible. More precisely, the *T*_g_ value should be sufficiently below the recrystallization temperature of the glass (*T*_r_) and the *T*_d_ of the MOF.^28^ Above *T*_g_, the viscosity (η) of the glass must decrease enough (log η ∝ 1/*T*) to be able to flow around the MOF particles, resulting in a more cohesive composite with better mechanical properties.

**Figure 3:**
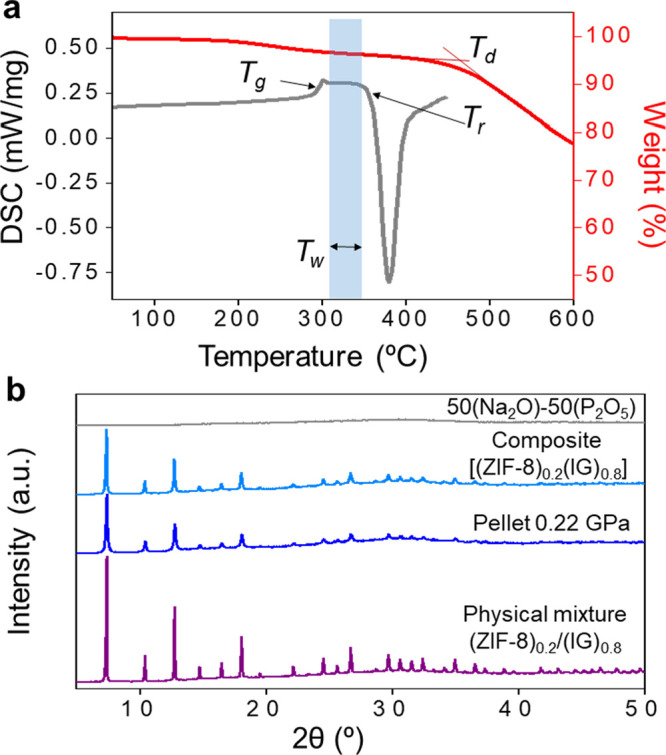
(a) DSC curve of the inorganic glass (gray) and TGA of ZIF-8 (red). *T*_g_, *T*_r_ and *T*_d_ were calculated for inorganic glass and ZIF-8, respectively. Potential *T*_w_ range is depicted in blue. (b) PXRD patterns for (ZIF-8)_0.2_/IG)_0.8_ physical mixture (purple), pelletized mixture (blue), [(ZIF-8)_0.2_(IG)_0.8_] composite prepared at 310 °C (cyan), and inorganic glass (gray).

ZIF-8 and the inorganic glass were mixed in the required amounts by ball-milling to ensure sample homogeneity and then pelletized at different pressure values: 1, 0.74, 0.5, and 0.22 GPa (see Supporting Information, Figure S12). The pellets were thermally treated under a vacuum, at different *T*_w_ values above *T*_g_*,* but without exceeding the *T*_r_ of the inorganic glass (Figure 3a). The optimal conditions were found using 0.22 GPa during the pelletization before isothermal heating at 310 °C (*T*_w_) for 30 min. At this pressure, the Bragg intensities of the crystalline component (ZIF-8) are maintained and a bulk robust pellet is formed (Figure 3b).

### Characterization of the MOF-CIGCs

Using the aforementioned conditions, two additional samples with ZIF-8 weight percentages of 10 and 30% were synthesized. Liquid-state ^1^H NMR spectra (Figure S13) of the acid-digested 30% ZIF-8-containing samples indicated an unaltered ZIF-8 composition. PXRD from the physical mixtures showed that the intensities of the Bragg peaks belonging to ZIF-8 increase with the increasing amount of ZIF in the material, while the diffuse scattering contribution is higher with a larger content of the inorganic glass (Figures S14–S16). PXRD patterns from samples after pelletization showed a reduction of the intensity of the Bragg peaks, indicating a partial loss of crystallinity in the ZIF-8. However, the presence of the inorganic glass in the physical mixture reduces this effect, compared to the effect of pelletization on pristine ZIF-8 (Figure S17). This suggests the glass matrix may protect the ZIF-8 from amorphization under pressure. Surprisingly, the Bragg peak intensities were higher for the composite after the thermal treatment than before, indicating that the ZIF-8 structure recovers crystallinity during heating, similar to an annealing process (Figure S18, Table S3).^29^ Pawley refinements of PXRD from the three compositions of the MOF-CIGC showed a small contraction of the network after pelletization and thermal treatment in each case (Figures S19–S21 and Table S4).

DSC experiments on the three composites and the pure glass were carried out after TGA experiments (Figure S22). First, one cycle until 420 °C was measured for each sample, to observe the recrystallization process (Figure S23). The recrystallization temperature of the ball-milled sample of the pure glass was shifted to lower temperatures due to the higher amount of water and potential defects at the surface of the glass after ball-milling. Surprisingly, both the *T*_g_s and *T*_r_s of the physical mixtures and composites did not change appreciably, in contrast to the pure sample. We ascribe this to ZIF-8 being able to capture water from the surface of the inorganic glass, thus preventing changes in glass structure and its thermal behavior. Alternatively, the ZIF may act as a third component of the glass mixture, stabilizing the glass network, similar to ternary ZnO–Na_2_O–P_2_O_5_ glasses.^30^ The PXRD pattern after the recrystallization process confirmed the formation of a mixture of Na_3_P_3_O_9_ and the existing ZIF-8 (Figure S24).

Different heating/cooling cycles were performed to check the stability of the composites and physical mixtures upon heating as well as *T*_g_s after the first cycle described above. The maximum temperature applied in these DSC experiments was 320 °C to avoid recrystallization of the inorganic glass. During the first upscan of the physical mixtures, a broad endothermic peak between 30 and 140 °C is observed and related to the release of adsorbed water (Figures S25–27).^28^ The presence of a glass transition at *T*_g_ (∼297 °C) confirms the glassy nature of 50(Na_2_O)–50(P_2_O_5_). This *T*_g_ value is maintained after three upscans for all the materials (both physical mixtures and composites), where second and third upscans are almost equivalent in all cases (Figures S25–30). A reduction in the *T*_g_ overshoot on the second-up scans was observed for the three MOF-CIGCs, which may again be attributed to relaxation processes above *T*_g_ (Figures S28–31). Structural relaxation in supercooled liquids is related to the atomic rearrangements during isothermal annealing processes.^31^

**Figure 4:**
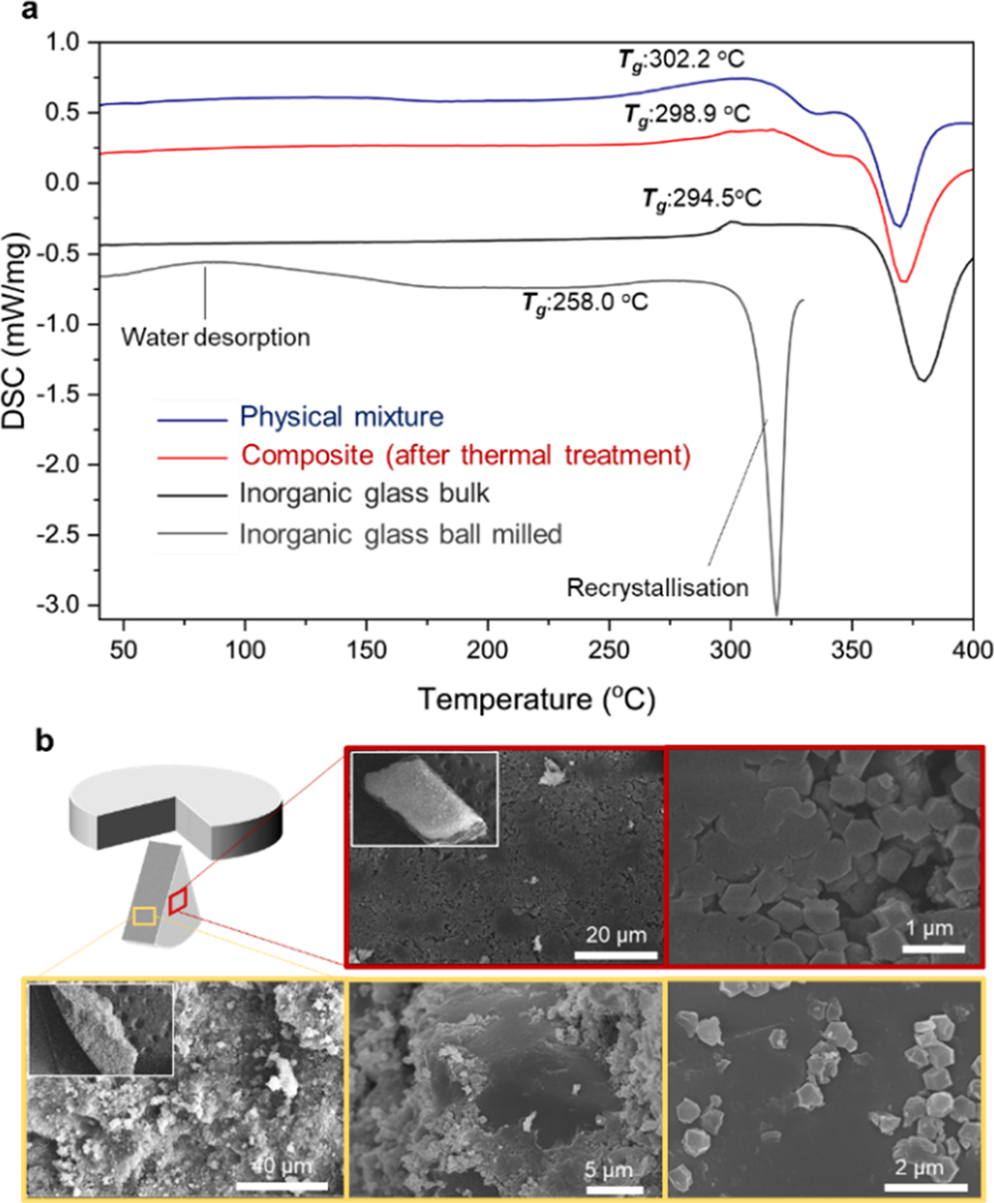
(a) First DSC upscans of [(ZIF-8)_0.2_(IG)_0.8_], its physical mixture ((ZIF-8)_0.2_/(IG)_0.8_), and the inorganic glass as bulk and after ball milling. (b) [(ZIF-8)_0.2_(IG)_0.8_] material, where SEM images were taken from the surface (red edge box) of the pellet (yellow edge box) and from inside the pellet showing ZIF-8 particles embedded in the inorganic glass matrix.

Comparing the DSC curves of the inorganic glass with the composites and physical mixtures, it is clear that the presence of ZIF-8 within the glass matrix appears to delay the recrystallization process relative to that of the ball-milled sample. This is more evident in the case of the composites than in physical mixtures (i.e., prior to pelletization and thermal treatment) (Figure 4a).

SEM and EDS analyses were performed to investigate the crystal-glass microstructure and the chemical composition of the composites. Two pieces of each sample were analyzed, each from markedly different orientations of the pelletized composite (Figures 4b and S32 and 33). First, a horizontal orientation was analyzed to explore the surface of the pellet. This indicated a rough surface containing different domains. Small cuboctahedra can be distinguished as ZIF-8 crystallites that are partially embedded in smooth inorganic glass domains. Moreover, several minor cracks between domains were highlighted by mapping (Figures S34–36). The presence of ZIF-8 is more evident in the case of [(ZIF-8)_0.3_(IG)_0.7_] MOF-CIGC due to the higher proportion of ZIF material in the composite. To ensure proper representation of the bulk sample, pieces from a vertical cross-section of the pellet were examined (Figures 3b and S37–S39). ZIF-8 crystallites were clearly embedded within the inorganic glass matrix. EDS analyses were carried out in five different points of ∼300 μm^2^ per sample to determine the percentage of Na, P, and Zn (Figures S40–S42) showing homogeneous distributions of all three elements.

To further investigate the structure of these materials, synchrotron X-ray total scattering measurements were collected at the I15–1 beamline at the Diamond Light Source (see Supporting Information). The data were processed to account for absorption and various scattering corrections using the GudrunX software to produce total scattering structure factors, *S*(*Q*), of all the materials.^32−34^ The *S*(*Q*) of the 50(Na_2_O)–50(P_2_O_5_) glass is consistent with the observed PXRD pattern (Figure S43). As expected, Bragg peaks are only observed in the *S*(*Q*) from samples that include crystalline ZIF-8 material (Figures S43–44). PDFs were produced by Fourier transform of the *S*(*Q*) data.^35^ Here, we use the *D*(*r*) function in order to accentuate high *r* correlations.^34^ By comparing with the calculated total and partial PDFs of Na_3_P_3_O_9_ (Figure S45), the three major peaks at 1.54, 2.47, and 3.43 Å in the inorganic glass *D*(*r*) are associated with P–O, Na–O, and Na···P correlations, respectively (Figure S46).^36^ The *D*(*r*) pattern of ZIF-8 shows characteristics correlations previously described in the literature (Figure S47).

**Figure 5:**
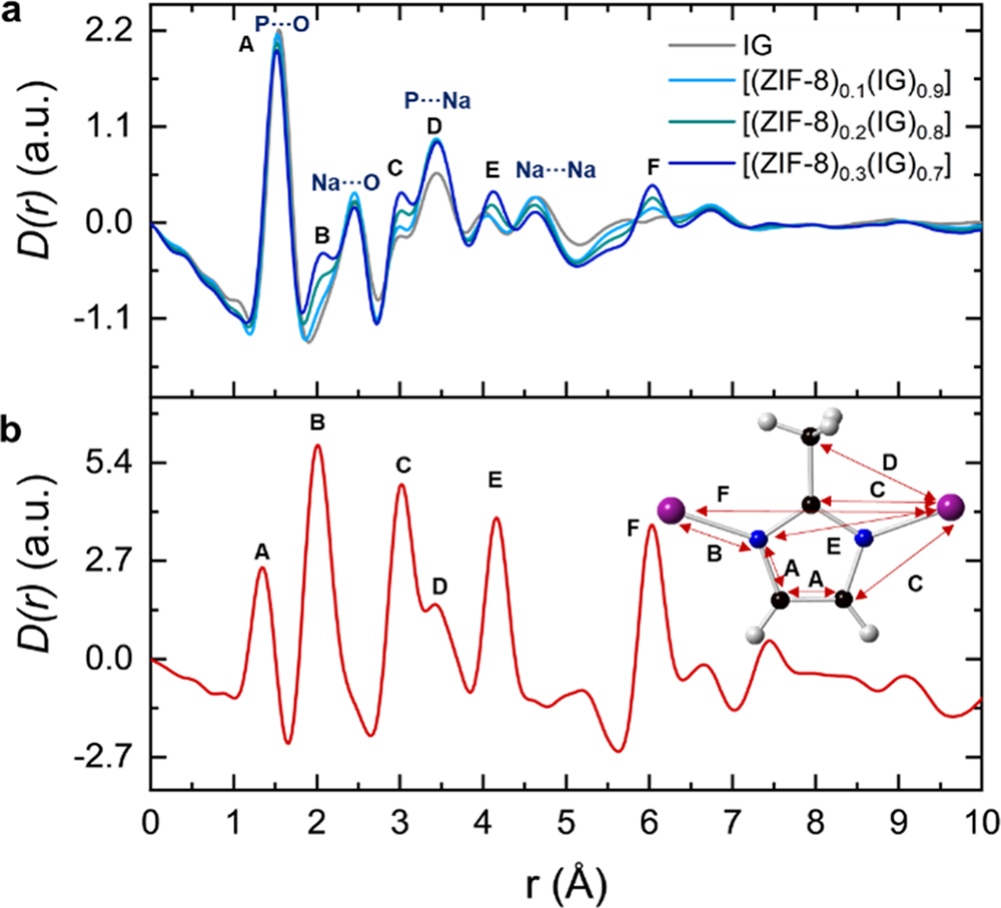
(a) PDF curves of the MOF-CIGCs and the inorganic glass, where correlations from inorganic glass are labeled in blue, and ZIF-8 correlations are assigned as letters. (b) *D*(*r*) function of pristine ZIF-8 with the main peaks assigned to correlations in the picture located at the top-right of the graph. Carbon (black), nitrogen (blue), Zn (purple), and hydrogen (light gray).

PDFs of the MOF-CIGCs and their corresponding physical mixtures show the same main correlations as their constituents, which confirms the structural integrity of each component (Figure S48). The first correlation located at 1.54 Å contains contributions from P–O, C–C, and C–N. The second correlation at 2.07 Å is the Zn–N distance (correlation labelled B), characteristic of the ZnN_4_ coordination environment (Figures 5 and S49). The peak located at 2.46 Å may arise from a Na–O correlation given the higher content of the glass. Zn···C_Im_ (C), Zn–N_Im_ (E), Zn···Zn (F) correlations are at 3.01, 4.08, and 6.05 Å, respectively. The differences in these three peak intensities are proportional to the amount of ZIF-8 in the composite. Peaks at 3.46 and 4.63 Å may be related to P···Na and Na···Na in the glass, respectively. In general, the peaks from the glass are more intense than those from ZIF-8 because there is more IG in each sample and their intensities do not vary greatly between samples. The latter is because, whereas the amount of ZIF-8 changes by a factor of 3 between samples with *x* = 0.1 and 0.3, the amount of IG only changes by (1–0.3)/(1–0.1) = 0.78.

### Study of the Interface between Glass and MOF

Understanding the interface between different components in composites is crucial for predicting the chemical and physical properties of these materials. However, structural studies of the interfacial regions within MOF composites are extremely challenging, given the low volume of interaction and the need for characterization to be carried out at the local atomic scale.

Fourier transform infrared (FTIR) and Raman spectra were collected with the purpose of identifying the possible interaction at the interface. This technique was previously used to identify a potential P···N interaction between an inorganic glass and a ZIF-62 glass.^37^ However, the FTIR spectra measured here had insufficient resolution to distinguish bands other than those typically expected from ZIF-8 and the inorganic glass (Figures S50 and S51). Furthermore, in-depth Raman analysis was impeded by a strong background signal observed for all the composites, making the detection of additional vibrational characteristics and their assignment to interaction at the interface impossible (Figure S52).

Evidence for correlations at the interface between the ZIF-8 and IG components in the composite was provided by multidimensional multinuclear MAS NMR spectroscopy, which is well equipped to probe spatial proximities.^38,39^ This approach is strongly suited to glass-containing systems because NMR is not dependent on the presence of long-range order but on nuclear spin interactions between NMR-active nuclei that are plentiful in the MOF-CIGC targeted here. In particular, this work focuses on the very sensitive spin 1/2 nuclei (high natural abundance, high Larmor frequency) in each of the individual components of the MOF-CIGC components, namely, ^1^H and ^31^P from the ZIF-8 and IG glasses, respectively. NMR spectra of other active nuclei (^13^C, ^15^N, both spin 1/2; ^23^Na quadrupolar nucleus with spin 3/2; and ^67^Zn spin 5/2) were also obtained to identify potential Zn···P, Na···N, and Na···C correlations.

**Figure 6:**
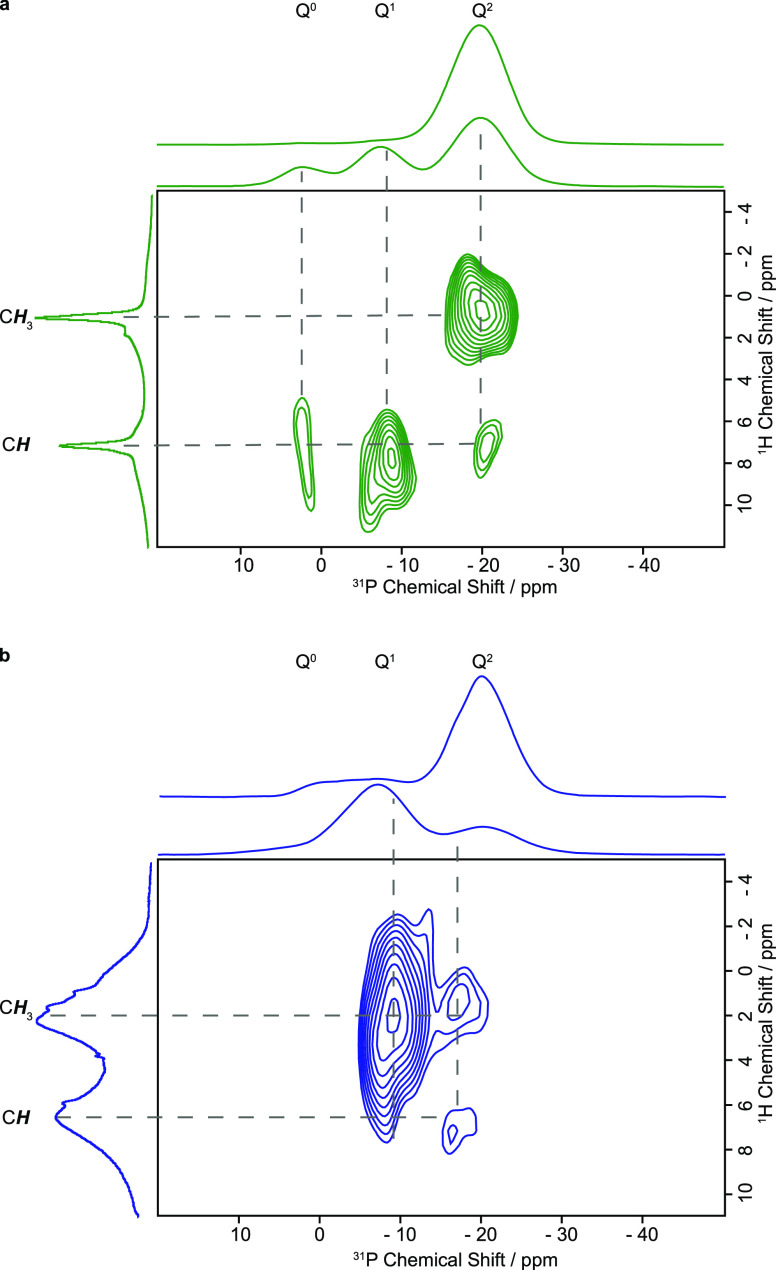
Two-dimensional ^1^H ^31^P HETCOR NMR spectra at 9.4 T under a MAS frequency of 10 kHz of (ZIF-8)_0.3_/(IG)_0.7_ physical mixture and (b) [(ZIF-8)_0.3_(IG)_0.7_] MOF-CIGC, demonstrating the spatial proximities between ZIF-8 and the inorganic glass matrix. A short contact time for CP of 50 μs was used and a recycle delay of 6 s (at least 1.3 times the ^1^H spin lattice relaxation time constant *T*_1_). The vertical spectra depict the ^1^H MAS spectra (Figure S58) while the horizontal spectra display the ^31^P MAS spectra under several experimental conditions (^31^P direct excitation, Figure S53, and ^1^H ^31^P CP, Figures S54–56, on top and bottom, respectively). The spectral assignments are given in the figure and highlight the close proximities between CH and CH_3_ of the ZIFs and *Q^n^* of the phosphate tetrahedron.

The ^31^P directly excited MAS NMR spectra of the (ZIF-8)_0.3_/(IG)_0.7_ physical mixture, the [(ZIF-8)_0.3_(IG)_0.7_] composite and 50(Na_2_O)–50(P_2_O_5_) recorded under quantitative conditions (Figures 6 and S53 for a direct comparison) had a dominant signal at −20 ppm. This is typical of *Q*^2^ metaphosphate tetrahedral units. Minor signals around −10 (*Q*^1^) and 0 ppm (*Q*^0^) are ascribed to a very small degree of depolymerization or the slight off-stoichiometry of the 50:50 Na_2_O:P_2_O_5_ ratio (Table S1). Upon composite formation of [(ZIF-8)_0.3_(IG)_0.7_], these *Q*^1^ and *Q*^0^ signals appear more intense than in the (ZIF-8)_0.3_/(IG)_0.7_ physical mixture and the inorganic glass, 50(Na_2_O)–50(P_2_O_5_) as shown in Figure S53, potentially due to interface interactions as explored below. The ^23^Na MAS NMR spectra of all three materials (Figure S54) are virtually identical with an asymmetrically broadened resonance centered at about −15 ppm and a low-frequency tail capturing a continuous distribution of quadrupolar parameters as typically observed for structural disorder in glassy materials.^40^ These data further reinforce the structural integrity of the inorganic glass upon mixing and thermal treatment captured by PDFs.^41^

In order to probe spatial proximities, ^1^H ^31^P cross-polarization (CP) MAS NMR spectra of the (ZIF-8)_0.3_/(IG)_0.7_ physical mixture and the [(ZIF-8)_0.3_(IG)_0.7_] composite were recorded (Figures 6 and S55–57). In this experiment, a transfer of polarization occurs between the highly polarized spin (i.e., ^1^H) and the weakly polarized spin (^31^P) and establishes a heteronuclear dipolar contact that is related to their internuclear distance, although a CP spectrum is not a quantitative measurement of the site distributions. Strong ^1^H ^31^P CP MAS NMR signals for *Q*^0^, *Q*^1^, and *Q*^2^ are observed in both (ZIF-8)_0.3_/(IG)_0.7_ physical mixture (Figure 6a) and [(ZIF-8)_0.3_(IG)_0.7_] composite (Figure 6b), associated with polarization transfer from the ^1^H in the ZIF-8 to ^31^P of the phosphorus tetrahedra in the inorganic glass. Low intensity ^31^P CP signals are also observed in the inorganic glass and result from the very weak ^1^H signals (Figure S58) in this phase, which likely arises from POH-like moieties in *Q^n^* environments.

To provide site-specific atomic-scale resolution, two-dimensional ^1^H ^31^P heteronuclear correlation (HETCOR) spectroscopy was performed. This correlates the ^1^H signals with the neighboring ^31^P resonances, enabling spatial proximities in the physical mixture and composite to be determined. The resulting HETCOR spectra (Figure 6) both show the presence of sets of correlations between the C*H* (at ∼6–8 ppm, typical for this type of ^1^Hs)/C*H*_3_ (at ∼2 ppm) groups of the ZIF-8 with the *Q*^2^ (−20 ppm)/*Q*^1^ (−10 ppm)/*Q*^0^ (0 ppm) signals of 50(Na_2_O)–50(P_2_O_5_) within the glass–MOF that are not present in POH moieties in the inorganic glass itself (Figures S59 and S60 for a comparison of the HETCOR spectra with those of the physical mixture and MOF-GCIGC). These correlations unequivocally illustrate the presence of close H···P proximities between the ZIF and inorganic glass. Some differences between the HETCOR spectra for the physical mixture and the composite can be discerned, though all broadly point to proton and phosphorus sites being in close proximity. Because it relies on a CP step, the ^1^H ^31^P HETCOR spectra are not quantitative, and therefore the intensity of the signals is not indicative of the strength of the observed interactions.

^13^C and ^15^N CP MAS NMR spectra have previously been extensively used to confirm the retention of chemical composition and provide evidence for imidazolate linker decoordination during (ZIF) amorphization.^42^ Both ^13^C and ^15^N CP MAS NMR spectra of the (ZIF-8)_0.3_/(IG)_0.7_ physical mixture and [(ZIF-8)_0.3_(IG)_0.7_] composite (Figures S61 and 62) resolved the crystallographically different carbon and nitrogen atoms with narrow resonances (for example, a full width at half-maximum of 50 Hz was measured for the CH in [(ZIF-8)_0.3_(IG)_0.7_], Figure S61b) that are commensurate with crystalline ZIF-8 as expected. Two signals at 13.8 and 14.9 ppm are observed in (ZIF-8)_0.3_/(IG)_0.7_ for the methyl group (Figure S61a) while only one is seen in [(ZIF-8)_0.3_(IG)_0.7_] (Figure S61b). This may indicate hydrogen bonding between the ZIF-8 protons and the *Q*^1^, *Q*^2^, and *Q*^3^ terminal oxygens of the IG (by analogy with recent findings on sulfonyl-containing ionic liquid in the pores of ZIF-8) or phase separation before heat treatment.^43^

Recent work on ^67^Zn MAS NMR at very high field has also demonstrated that this low-receptive quadrupolar nucleus is very sensitive to short-range disorder in several ZIFs.^44^ The corresponding ^67^Zn spectrum for crystalline ZIF-8 at 18.8 T shows a single resonance, with second-order broadening from the quadrupolar interaction, for the crystallographically unique Zn tetrahedra that are largely maintained in the (ZIF-8)_0.3_/(IG)_0.7_ physical mixture (Figure S63). Upon thermal treatment and composite formation to [(ZIF-8)_0.3_(IG)_0.7_], the ^67^Zn MAS NMR spectrum further broadens slightly, with a clear change in second-order quadrupolar line shape (Figure S63), perhaps indicating an interaction of the inorganic glass with the Zn sites. It is worth pointing out that this broadening is significantly smaller than that observed for amorphous ZIF-8 (Figure S64), where the line shape is dominated by structural disorder around the Zn site, highlighting the retention of the short-range order in ZIF-8 in the [(ZIF-8)_0.3_(IG)_0.7_] composite.

To provide as detailed a picture as possible of the ZIF–inorganic glass interface, differential analysis of the PDF (dPDF) data was also carried out. This methodology has already been applied in Zr-MOFs to unveil local structural transitions for oxocluster structures.^45,46^ Typically, dPDF consists of subtracting the PDF of the pristine MOF from that of the modified material using a normalization constant to minimize the residual at positions where the PDFs should be identical, such as at the C–C, C–N, and C–O linker correlations.

However, deciphering correlations at the glass–MOF interface is even more challenging given the presence of at least three components: ZIF-8, the inorganic glass, and the interface between this glass and ZIF-8. To attempt to simplify things, we subtracted the *D*(*r*) of the physical mixtures from that of the composite, aiming to minimize the P–O correlation (at *r* = 1.52 Å), which should be unchanged (Figure S65). However, these dPDFs were extremely difficult to analyze due to the complexity of this process.

Another approach to differential PDF analysis instead subtracts the normalized scattering data of the physical mixture from that of the corresponding composite (Figure S66). This difference *S(Q)* is then Fourier transformed to obtain the dPDF. These dPDFs showed similar features for all the composites at long range, in addition to two new correlations at short range for [(ZIF-8)_0.3_(IG)_0.7_] (Figure S67). The presence of two peaks located at 2.50 and 3.38 Å would be consistent with Na···N and Zn···P correlations in zinc phosphate structures (Figure S68).^47^ However, these peaks appear in only one of the samples, which suggests this approach is unreliable for these systems.

Multivariate analysis is a tool that can reduce complex data into its components. It has previously been employed to observe the correlations at the interface of composite materials.^48^ It has however not been widely extended to the characterization of complex hybrid materials. Recently, one of the principal methods of multivariate analysis, known as principal component analysis (PCA), has been employed to study disorder in an amorphous MOF, Fe-BTC.^49^ The components extracted from PDFs using PCA are not necessarily chemically intuitive, given the purely mathematical approach used. However, they *may* correspond to atom–atom correlations, distortions, noise within the data, and compensation resulting from the requirement that the components are orthogonal. To interpret PCA of PDF, the first principal component (PC), i.e., with the largest eigenvalue, accounts for the largest proportion of the total variance in the data and should approximate to the PDF from the largest contributor to the composite PDF. In this case, this would be the IG PDF. The second PC accounts for the next largest proportion of the variance (mainly from the second largest contributor, the ZIF-8 PDF), and so on.

As outlined above, PCA does not constrain the form of the components by component weightings, and the raw PC output can include components and weightings that are linear combinations of the PDFs and abundances of the distinct phases within the sample.^50^ The big advantage is that it treats the data set as a whole. In this case, PCA was applied to the set of four *D*(*r*) functions of all the materials labeled by weight % of ZIF-8 in the mixture: 0% (pure IG), 10, 20, and 30% composites and 100% (pristine ZIF-8). From these, four PCs were extracted, and of these, only three were deemed statistically significant with 77.25, 22.88, and 0.44% of the variance, respectively (Figure S69). PC4 (0.12%) did not contain any correlations above the background noise level and was therefore without any potential physical significance.

The first PC (PC1) largely corresponds to glass correlations, while the second (PC2) matches the PDF from ZIF-8 (Figures S70 and S71). However, the third component (PC3) does not fit with either and exhibits some features at short-*r* value similar to those of a PDF measurement from the empty capillary. There is also a small additional peak at 2.18 Å and a stronger negative peak at 3.44 Å (Figure S72). The weighting of PC1 for each sample decreases in line with the reduction of the amount of glass in the composite across the series, while the PC2 weighting increases with the increase of ZIF-8 in the composite (Figure S73). However, the weightings of PC3 have a lower contribution (in absolute value) for the composites than the pristine materials. The negative weighting to PC3 for the composites suggests that -PC3 may be the more physically relevant function, i.e., the empty capillary may have been oversubtracted in the data normalization, giving rise to a (now) negative peak in PC3 from its Si-O bond at ∼1.6A, and there is potentially some interaction at the interface, with a positive feature at 3.40 Å, which might be related to the interaction P–O–Zn between the ZIF-8 and the glass at the interface (Figure S68b).

In an effort to better understand the results obtained from PCA and dPDF, a third analysis method, namely, multilinear regression (MLR) analysis of *D*(*r*) functions, was employed. This approach fits the composite *D*(*r*) functions using proportions of the pristine ZIF and inorganic glass *D*(*r*) functions, which can be considered as end members of the compositional series, according to eq 1. The residual difference between the experimental and the calculated composite *D*(*r*), denoted as residuals, may reveal any interactions at the interface as they cannot be present in the pristine ZIF-8 and pure IG *D*(*r*) functions.
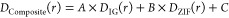
1

The refined weightings for the glass and ZIF-8 PDFs follow the expected trends based on the amount of each material in the composites (Figure S74).

**Figure 7:**
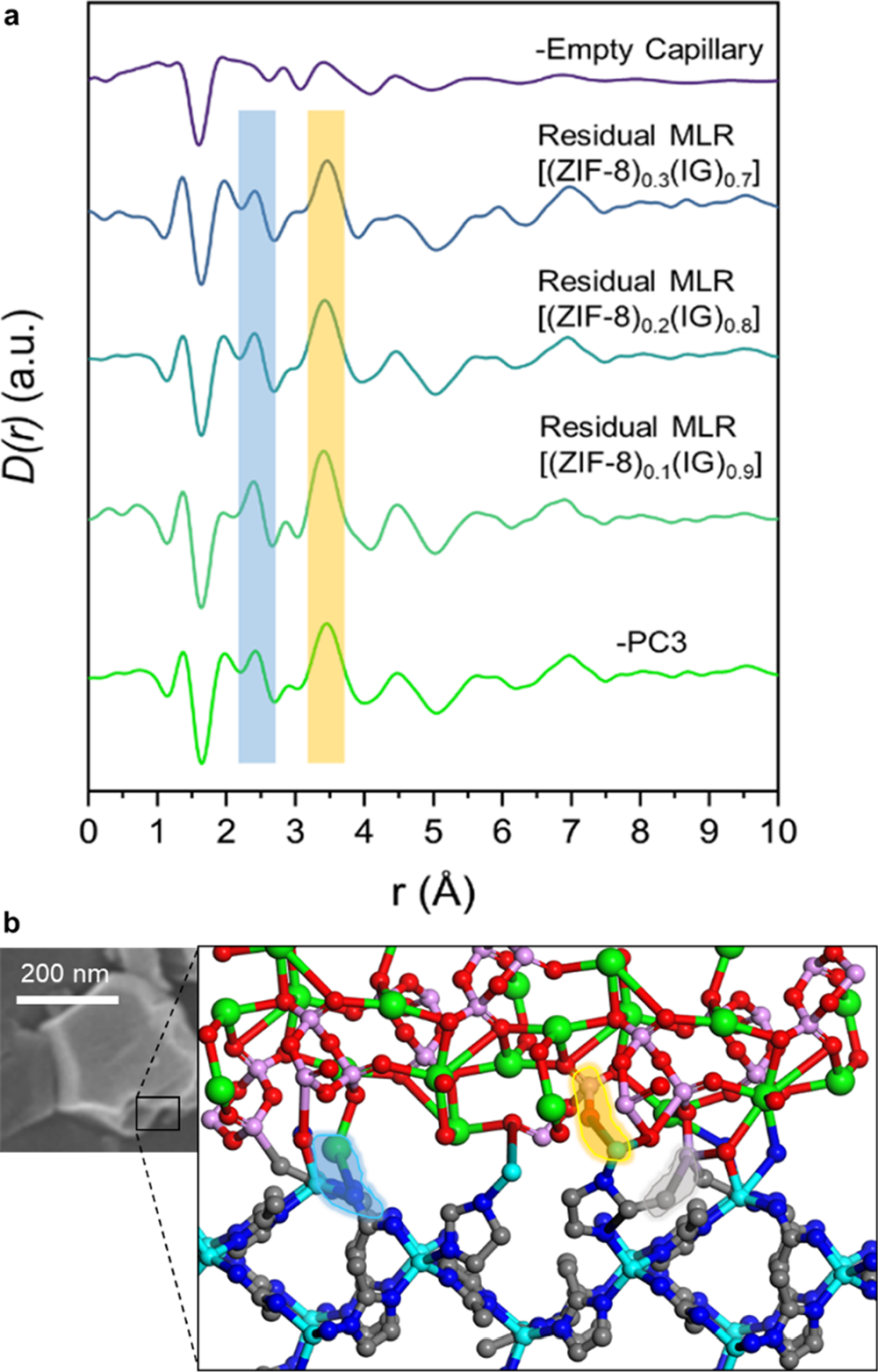
(a) Comparison between scaled borosilicate capillary, residuals of the multilinear regression, and negative PC3 exhibit similar features. New correlations at 2.45 and 3.35 Å at PC3 and residuals are highlighted in blue and yellow, respectively. (b) SEM image and schematic depiction of the interaction at the interface glass-ZIF-8 with the spatial proximities for H···P (gray highlights) and new interactions for Na···N (blue highlights) and Zn···O···P (orange highlights). Zn (cyan), C (gray), N (blue), Na (violet), O (red), P (green). Hydrogens were omitted for clarity.

In all cases, the calculated PDF functions of the composites fit well with good correlation coefficients and *C* values close to zero (Table S6, Figure S75). Very similar to PC3, the residuals show correlations related to the empty capillary and additional peaks at 2.45 and 3.35 Å (Figures 7 and S77). These additional peaks are coincident with the Na···N and Zn···O···P atomic distances compared to the literature (Figure S76), further supporting the ^15^N and ^67^Zn MAS NMR data postulating these interactions. Features at 1.36 and 1.97 Å might be associated with ZIF-8 correlations (C···C and C···N and Zn···N). In fact, samples containing a higher amount of ZIF-8 in the composite have a greater contribution of both peaks. The negative peak at 1.63 Å is probably from a mis-subtracted capillary contribution, corresponding to the Si**–**O bond in silicate glasses. These interactions complement the H···P correlations identified by MAS NMR (Figure 7b). H···P correlations are not observed by X-ray total scattering due to the low contribution of hydrogen atoms at the total PDF. This also suggests that ZIF-8 is often linker terminated,^51^ and that the interaction at the interface has a higher proportion of H···P and Na···N. However, these interactions have a lower contribution to the PDF compared with atom pairs including electron-rich atoms and the resolution in our PDF analysis is insufficient to reliably observe them. These interactions at the interface might have a direct effect on the mechanical properties increasing their strength.

The likely thickness of the interfacial region is obviously of interest. By making some very broad assumptions about the interfacial density, size of ZIF-8 particles, and PC weightings obtained from PCA analysis (see SI), we estimate that the ZIF-8–inorganic glass interface is a maximum of 1 nm thick. This result is also in agreement with the MAS NMR data as the transfer of the polarization needed to capture ^1^H ^31^P 2D correlation would typically be within 1 nm. However, improved PDF (data with lower backgrounds, higher resolution, and collected from a larger set of samples) would be needed to obtain a more accurate value.

To evaluate the mechanical properties of the composite and, in particular, to confirm the presence of a cohesive interface, compression testing was performed under increasing compressive loads. Stress–strain diagrams (Figures S79–S81) confirm the greater ability of composites containing a higher amount of the more flexible ZIF-8 component to accommodate applied stress, which is evident from the earlier onset of sample cracking, seen in the shift toward higher strain. In other words, the more compliant nature of ZIF-8 prevents composite failure at low applied stresses.

At lower ZIF proportions in the composite, the sample’s mechanical response is more dominated by the partially sintered inorganic glass than by the combined response of both materials. This makes local brittle fractures more likely. At higher ZIF concentrations, however, the softer material (ZIF) can contribute to better stress distribution within the pellet, as expected from soft matrices in composite materials. Some hysteresis is evident at lower applied stress in the composite, particularly in the [(ZIF-8)_0.1_(IG)_0.9_]. This would be consistent with further microscopic densification in the pelletized sample, associated with local particle rearrangements according to the uniaxially applied stress. Above a particular maximum loading, the stress cannot be dissipated by rearrangement or stored as elastic energy, which induces crack formation within the pellet. From this point on, further loading leads to a gradually increased crack growth in each subsequent cycle.

### Gas Uptake and Stability Test

CO_2_ adsorption isotherms of the composites were collected to study whether the addition of ZIF-8 into the glass matrix results in a higher porosity. The results confirm that porosity from ZIF-8 could be imbued within the glasses (Table S9 and Figure S83).

Surprisingly, given that one might expect composite samples to possess a lower accessible porosity compared to physical mixtures (given the dense nature of the glass preventing the ingress of guest molecules), the differences between the physical mixtures and the composites were not substantial (Figure S84). This trend is likely due to the presence of microscale voids in the composites between domains (Figures 6 and S32–39). Some hysteresis processes are also visible in both composites and physical mixtures, probably because of the presence of narrow pore-size distribution from the partially amorphized ZIF-8 and the inorganic glass matrix. This behavior might happen due to slower diffusion of the carbon dioxide molecules within the framework than the experimental timescales.

Despite some of their high biocompatibility, phosphate glasses, especially binary ones, exhibit poor stabilities in ambient temperature and humidity because they are very hygroscopic. A stability test in air and phosphate buffer saline (PBS) solution monitored by PXRD demonstrated that the composite materials alone were more stable than the ball-milled inorganic glass (Figure S85). The presence of ZIF-8 in the composite prevents the recrystallization of the inorganic glass and increases stability against dissolution in PBS (Figures S86–S88). Moreover, stability tests in different polar solvents have also been explored. The composite [(ZIF-8)_0.3_(IG)_0.7_] showed good stability after 48 h immersion, according to the differences in mass and PXRD before and after the immersion (Table S10, Figure S89).

## Conclusions

These results describe a family of MOF–inorganic glass composites, which we call MOF-CIGCs, obtained by pelletization of ZIF-8 and 50(Na_2_O)-50(P_2_O_5_), followed by heating above the glass transition temperature of the latter. Unlike previous efforts, the ZIF-8 structure was maintained without loss of crystallinity upon heating. According to DSC, the addition of ZIF-8 to the glass also delays recrystallization of the inorganic glass upon heating, linked to its ability to remove (recrystallization-promoting) adsorbed surface water from the glass itself.

The nature of the interface between the ZIF and the inorganic glass is best obtained from considering both the residues of the MLR analysis of the X-ray PDFs data (among other analysis methodologies) and multinuclear multidimensional NMR spectra capturing spatial proximities. These MOF IG interactions are namely Zn···P, N···Na, and H···P. This MLR approach might be more suitable for small data series with a small number of components, whereas PCA might be more suitable for analyzing more complex composites. The contribution to the PDFs from interfacial correlations is, however, inevitably small and is at the limit of the accuracy of current experiments, and the same is true for the MAS NMR data.

The next step is clearly to determine the role of the interface in directing the composite′s physical properties. Key to this is the deduced size of the interfacial regions of the MOF-CIGCs. Our analyses of the PDF data that the interface is probably on the order of 1 nm thick, albeit with a large uncertainty and based on features in the data that are the limit of the current measurements. Improvements in the experimental PDF method are underway and will hopefully provide more accurate results in the near future.

Correlated ^1^H ^31^P NMR spectra between nuclear spins are uniquely present in each of the MOF-CIGC components and provide a powerful approach to probing neighboring environments, which are also postulated from NMR data on other nuclei. The demonstration of a MOF IG interaction in our case predicts a better mechanical performance compared to ZIFs, often obtained as microcrystalline powders. Interaction between individual components in MOF-composite materials has been previously computationally predicted,^52^ through this work proves that the experimental elucidation of these interactions is also possible.

The transference of the chemically desirable properties of ZIF-8 to the glass is seen in the surprisingly high CO_2_ sorption values for the composite samples. Improved glass stability in air and PBS is a further key result, which bodes well for future studies of glass stabilizers. Hence, this new family of materials opens new and exciting avenues to prepare new MOF-CIGCs for diverse applications such as photocatalysis (Ti-MOFs) or even biomedical applications such as bone regeneration.

## Materials and Methods

### PXRD

PXRD data were collected on a Bruker D8 DAVINCI diffractometer equipped with a position-sensitive LynxEye detector with a Bragg–Brentano parafocusing geometry. Cu Kα1 (λ = 1.5406 Å) radiation was used through a 0.012-mm Ni filter. The samples were compacted into 5 mm disks on a low-background silicon substrate and rotated during data collection in the 2θ range of 2–50° at ambient temperature.

### TGA

TGA curves were conducted using a TA Instruments Q-650 series. Approximately 5–10 mg of powdered samples were placed in open 90 μL alumina crucibles. The samples were left to equilibrate for 5 min at 30 °C under an argon flow of 100 μL/min before the thermal treatment. A thermal heating using ramp of 10 °C/min was applied between 30 and 800 °C. Data were analyzed using TA Universal Analysis software.

### DSC

DSC curves were recorded on a NETSCH DSC 214 Polyma instrument. Approximately 5–10 mg of powdered samples were placed in sealed 70 μL aluminum crucibles with a hole punctured in the lid to prevent pressure build-up. An empty aluminum pan was used as a reference. Background corrections were performed using the same heating cycle on an empty aluminum crucible. All data analysis was performed using the Netzsch Proteus software package.

### CHN Microanalysis

CHN combustion analysis experiments were performed using a CE440 Elemental Analyzer, EAI Exeter Analytical Inc. ∼1.3–1.5 mg of sample was used for each run. Measurements were collected two times per sample.

### SEM and EDS Analysis

SEM images were collected with a high-resolution scanning electron microscope FEI Nova Nano SEM 450, with an accelerating voltage of 15 kV for image acquisition and 20 kV for EDS collection. All samples were prepared by dispersing the material onto a double-sided adhesive conductive carbon tape that was attached to a flat aluminum sample holder and was coated with a platinum layer of 15 nm using an Emtech K575 sputter coater.

### Total X-ray Scattering – PDF

X-ray total scattering data were collected at beamline I15-1, Diamond Light Source, UK (EE200338) on pristine glass, pristine ZIF-8, composites, and physical mixtures. All samples were ground and loaded into borosilicate glass capillaries (0.78 mm inner diameter) to a height of 3.6 cm. The capillaries were sealed with plasticine before being mounted onto the beamline. Total scattering data were collected at room temperature for the background (i.e., empty instrument), empty borosilicate capillary, and for all samples in a *Q* range of 0.2 – 26.0 Å^–1^ (λ = 0.189578 Å, 65.40 keV). The total scattering data were processed to account for absorption corrections and various scattering corrections including background scattering, multiple scattering, container scattering, and Compton scattering, in a *Q* range of 0.35 – 20.0 Å^–1^. Sample densities required for the data processing were obtained from helium pycnometer measurements. Subsequent Fourier transformations of the processed total scattering data resulted in a real-space PDF *G*(*r*) for each material. In this work, we use the *D*(*r*) form of the PDF to accentuate high *r* correlations. All processing of the total scattering data was performed using GudRunX following well-documented procedures.^32−34^

### FTIR

IR spectra were collected on powder samples by using a Bruker Tensor 27 FTIR spectrometer in transmission mode between 550 and 4000 cm^–1^. A background was subtracted from all spectra prior to analysis.

### Raman Spectroscopy

Raman measurements were performed using a confocal Raman microscope (Renishaw InVia) equipped with a suitable edge filter for the elastically scattered intensities and a 50x LD objective lens using 785 nm laser excitation to suppress fluorescence. Prior to sample characterization, the grating and detector were calibrated against a single crystal silicon reference sample. Spectra were collected in the range of 100–1250 cm^–1^ with ∼1.2 cm^–1^ resolution. Each measurement consisted of up to 60 individual accumulations at ∼1 s accumulation time to maximize the signal-to-noise ratio without oversaturating the detector.

### Liquid-State NMR Spectroscopy

Liquid-state ^1^H NMR experiments were carried out on a 9.4 T Bruker Avance III HD spectrometer equipped with a 5-mm BBFO probe. NMR samples were prepared by digesting 8 mg of the compound in 100 μL of 35 wt % DCl in D_2_O and dissolving the mixture in 500 μL of DMSO-*d*_6_. Chemical shifts are referenced to the 1H signal of the residual protons in DMSO-*d*_6_ at 2.5 ppm. The experiments were recorded with a recycle delay of 10 s and 32 scans.

### MAS NMR Spectroscopy

All ^1^H, ^13^C, ^15^N, ^23^Na, and ^31^P experiments were performed on a Bruker 400 MHz (9.4 T) Avance III HD NMR spectrometer equipped with a 4 mm triple-resonance HXY MAS probe in double resonance mode tuned to ^1^H at υ_0_(^1^H) = 400.1 MHz and ^31^P at υ_0_(^31^P) = 162.0 MHz or ^13^C at υ_0_(^13^C) = 100.6 MHz or ^15^N at υ_0_(^15^N) = 40.6 MHz or ^23^Na at υ_0_(^23^Na) = 105.8 MHz. Samples were packed in 4 mm ZrO_2_ rotors and spun under MAS at υ_r_ = 10 kHz for ^1^H, ^13^C, ^23^Na and ^31^P and at υ_r_ = 8 kHz for ^15^N. ^1^H pulses with radio frequency (rf) amplitudes of 56, 43.5, and 52.6 kHz were used in the ^1^H ^13^C, ^1^H ^15^N, and ^1^H ^31^P CP experiments. SPINAL-64 heteronuclear decoupling during ^13^C/^15^N/^31^P detection was carried out with ^1^Hrf amplitudes of 67 kHz for ^13^C experiments, 47 kHz for ^15^N experiments, and 62 kHz for ^31^P experiments.^53^^23^Na and ^31^P pulses for the directly excited spectra were obtained using 90° pulse lengths of 5.8 and 5.5 μs using rf field amplitude of 43 and 45.4 kHz, respectively. The Hartmann–Hahn matched conditions for CP were achieved using a ^13^C rf amplitude of 42 kHz ramped from 70 to 100% to obtain maximum signal at a ^1^H rf field amplitude of 56 kHz, a ^15^N rf field amplitude of 20 kHz ramped similarly to obtain maximum signal at a ^1^H rf field amplitude of 32 kHz.^54^ The contact times for all CP-based experiments are indicated in the figure captions and range between 50 μs and 2 ms. Frequency-switched Lee–Goldberg (FSLG) homonuclear decoupling at an rf amplitude of 62 kHz, and an LG offset of 0 Hz was used during the ^1^H evolution time in the two-dimensional (2D) ^1^H–^31^P heteronuclear correlation (HETCOR) spectra. Experimentally determined ^1^H scaling factors λ_exp_ for FSLG were measured by comparing the ^1^H spectra under MAS and the ^1^H projection of the 2D CP HETCOR for all samples and were found to be λ_exp_ = 0.7; these were then used to recover the full ^1^H chemical shifts in the 2D CP HETCOR from the scaled-down chemical shifts that resulted from decoupling. ^13^C/^15^N/^31^P CP and quantitative ^1^H MAS NMR spectra were respectively obtained with recycle delays of 1.3 (unless otherwise stated) and 5 times the ^1^H spin-lattice relaxation times T_1_ (measured via a standard saturation recovery experiment), while the ^23^Na and ^31^P MAS NMR spectra were obtained under quantitative relaxation assessed from variable recycle delay array experiments (typically 5 and 30 s, respectively). All ^67^Zn MAS NMR experiments were recorded on a Bruker 800 MHz (18.8 T) Avance Neo spectrometer equipped with a 3.2 mm HX probe tube to υ_0_(^67^Zn) = 50.09 MHz. The samples were packed in 3.2 mm ZrO_2_ rotors and collected under MAS at υ_r_ = 10 kHz. Spectra were obtained with a rotor-synchronized (3 rotor periods for crystalline ZIF-8/physical mixture/composite and 1 rotor period for amorphous ZIF-8) full echo pulse lengths of 5.5 and 11 μs, respectively, at a rf field amplitude of 15 kHz. A 2-ms double-frequency sweep from ± 800 to ± 200 kHz at an rf field amplitude of 3.5 kHz allowing inversion of the population of the satellite transitions leading to an experimental gain in sensitivity of approximately 1.3 was used.^55^ Experiments were recorded using recycle delays of either 1 or 0.1 s for crystalline ZIF-8/physical mixture/composite and amorphous ZIF-8, respectively. ^1^H, ^13^C, ^15^N, ^31^P, and ^67^Zn spectra were externally referenced to adamantane at 1.8 ppm, the tertiary carbon of adamantane at 29.45 ppm,^56^ glycine at 33.44 ppm,^57^ 85% H_3_PO_4_ in H_2_O at 0 ppm, and 1 M Zn(NO_3_)_2_ in D_2_O at 0 ppm, respectively. ^1^H chemical shifts are given with an accuracy in ±1 ppm which is typical of the slow MAS conditions used. All data were processed with Topspin software using standard procedures.

### Compression Test

The compression testing was performed using a universal mechanical testing machine (Zwick/Roell, Ulm, Germany), equipped with a flat steel base and a tiltable flat punch for self-alignment according to the sample tilt. Measurements were performed with a constant crosshead speed of 0.2 mm/min until a defined load was reached. Maximum loads were increased in 500N steps after each measurement to investigate the effects of previous loading on the mechanical response. To partially correct for the frame compliance of the testing machine, the elongation of the system was measured under the same loading conditions without any sample and subtracted from the data collected during sample measurement to derive the actual sample response.

### Gas Adsorption

Porosity measurements were performed on a Micromeritics ASAP 2020 surface area and porosity analyzer. Samples of ∼100 mg were degassed by heating under vacuum at 100 °C for 12 h, prior to analysis using carbon dioxide gas at 273 K. Gas uptake was determined using the Micromeritics MicroActive software.

### Gas Pycnometry (Density)

Pycnometric measurements were conducted with a Micromeritics Accupyc 1340 helium pycnometer. The typical mass used for each test was around 100 mg, and the reported value was the mean and standard deviation from 10 measurements.

## Supporting Information Available

Synthesis and characterization details, SEM images, PXRD, synchrotron X-ray total scattering data analysis, gas adsorption isotherms, stability tests, FTIR, MAS NMR, Raman, DSC and TGAs (PDF)
