# Supplementary material for: Interfacial Bonding between a Crystalline Metal–Organic Framework and an Inorganic Glass
Source: J Am Chem Soc. 2023 Oct 11;145(42):22913–24. doi: 10.1021/jacs.3c04248 (PMC10603780; doi:10.1021/jacs.3c04248)
Supplement: Supplementary file 1 — ja3c04248_si_001.pdf [file ja3c04248_si_001.pdf]

Supplementary information

Interfacial bonding between a crystalline metal-organic framework and an inorganic glass composite

Celia Castillo-Blas[a], Ashleigh M. Chester[a], Ronan P. Cosquer[b], Adam F. Sapnik[a], Lucia Corti[b,c], Roman Sajzew[d], Bruno Poletto-Rodrigues[d], Georgina P. Robertson[a], Daniel J.M. Irving[e], Lauren N. McHugh[b], Lothar Wondraczek[d], Frédéric Blanc[b,c,f*], David A. Keen[g] and Thomas D. Bennett[a*]

[a] Department of Materials Science and Metallurgy, University of Cambridge, Cambridge, CB3 0FS, United Kingdom.

[b] Department of Chemistry, University of Liverpool, Crown Street, Liverpool L69 7ZD, United Kingdom.

[c] Leverhulme Research Centre for Functional Materials Design, Materials Innovation Factory, University of Liverpool, Liverpool, L7 3NY, United Kingdom.

[d] Otto Schott Institute of Materials Research, University of Jena, Fraunhoferstrasse 6, 07743 Jena, Germany.

[e] Diamond Light source Ltd., Diamond House, Harwell Campus, Didcot, Oxfordshire, OX11 0QX, United Kingdom.

[f] Stephenson Institute for Renewable Energy, University of Liverpool, Crown Street, Liverpool L69 7ZF, United Kingdom.

[g] ISIS Facility, Rutherford Appleton Laboratory, Harwell Campus, Didcot, Oxfordshire, OX11 0QX, United Kingdom.

Email: frederic.blanc@liverpool.ac.uk, tdb35@cam.ac.uk

Table of contents

**1.**	**Synthesis and characterisation of ZIF-8**	3
**2.**	**Synthesis and characterisation of 50(Na_2O)-50(P_2O_5)**	5
**3.**	**Optimisation of the synthesis**	9
**4.**	**Liquid-state Nuclear Magnetic Resonance**	11
**5.**	**Powder X-ray diffraction**	12
**6.**	**Thermal characterisation**	17
**7.**	**Scanning electron microscopy study**	22
**8.**	**Pair distribution function study**	30
**9.**	**FTIR**	34
**10.**	**Raman Spectroscopy**	37
**11.**	**Nuclear Magnetic Resonance**	38
**11.**	**Differential pair distribution function study**	47
**12.**	**Principal component analysis**	49
**13.**	**Multiple linear regression**	52
**14.**	**Estimation of the interfacial thickness**	56
**15.**	**Compression tests**	58
**16.**	**Carbon dioxide isotherms**	60
**17.**	**Stability test**	62
**18.**	**References**	66

1. Synthesis and characterisation of ZIF-8

Synthesis of ZIF-8. ZIF-8 was prepared following a previously reported methodology.[1] A solution of 0.3 g (1.367 mmol) of zinc acetate in 5 mL of deionised water was added into a solution of 1.12 g (14.495 mmol) of 2-methylimidazole in 5 mL of deionised water. The mixture was homogenised by sonication for one minute. Then the mixture was left at room temperature for 24 hours. White nanocrystals were collected by centrifugation at 4000 rpm for 10 minutes and washed several times with methanol. Finally, the product was dried under dynamic vacuum at 120 °C for 16 hours. Yield (135 mg, 43.8%). CHN analysis calculated ($ZnC_8H_{10}N_4$) /found: C 42.22%/ 42.26%, H 4.43%/ 4.37%, N 24.62%/ 24.39%.

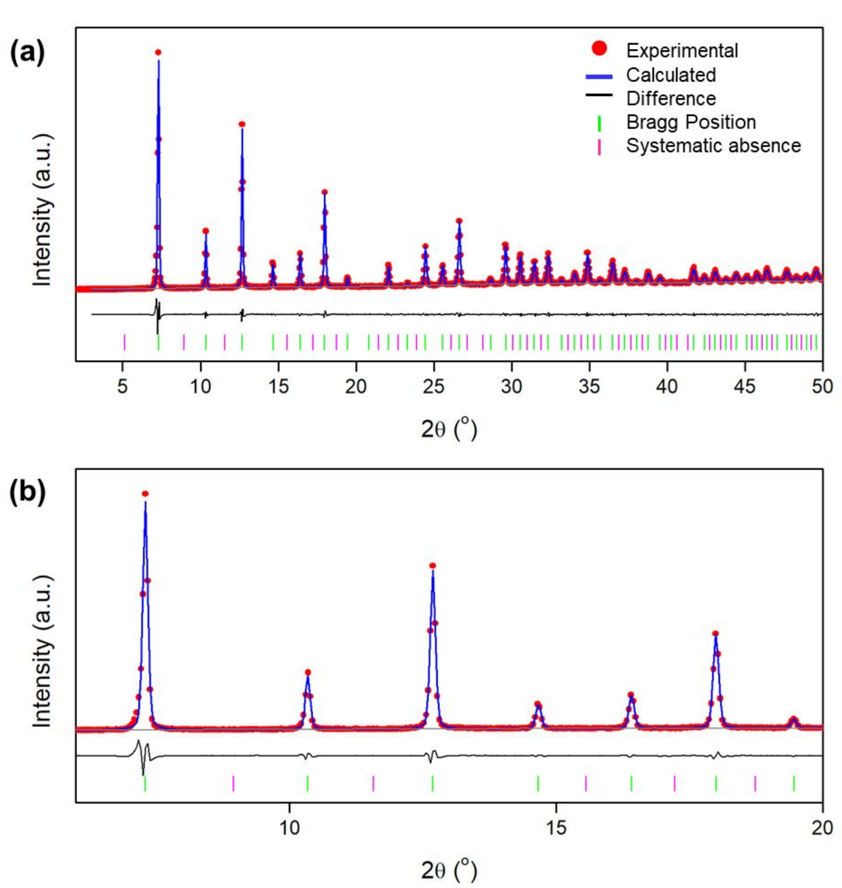

Figure S1. Experimental (red dots), calculated (blue line), difference plot plot [(I_{obs}−I_{calc})] (black line) and Bragg positions (green ticks), systematic absences (pink ticks) for the Pawley refinement of experimental diffraction data with the obtained cell: R_{wp} = 6.86%, R_p = 4.48%, a = 17.3725 (12) Å, space group: *I-43m* **(a)**; zoom-in between 3-20° 2θ **(b)**. Original lattice parameter found in the literature, a = 16.8509(3) Å.[2] Difference between the published lattice parameters and the experimentsl ones here may arise from the activation process, which we performed under dynamic vacuum at 120 °C for 16 hours.

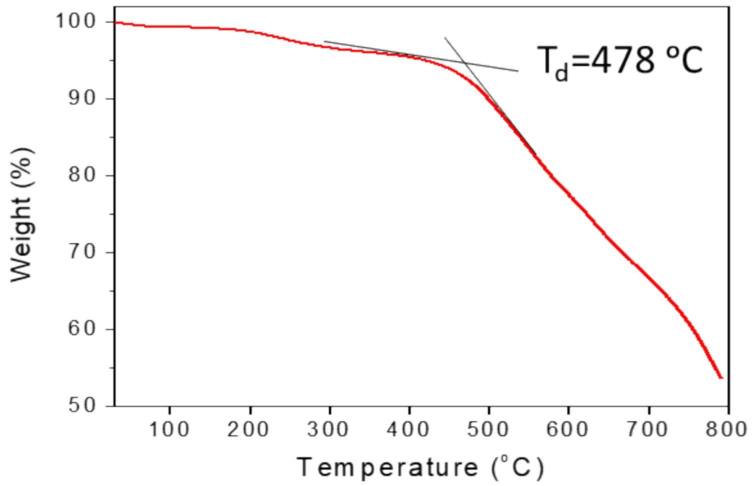

Figure S2. TGA of an evacuated sample of ZIF-8, performed under an argon flow with a 10 ºC/min heating ramp. T_d was calculated using TA Universal Analysis software.

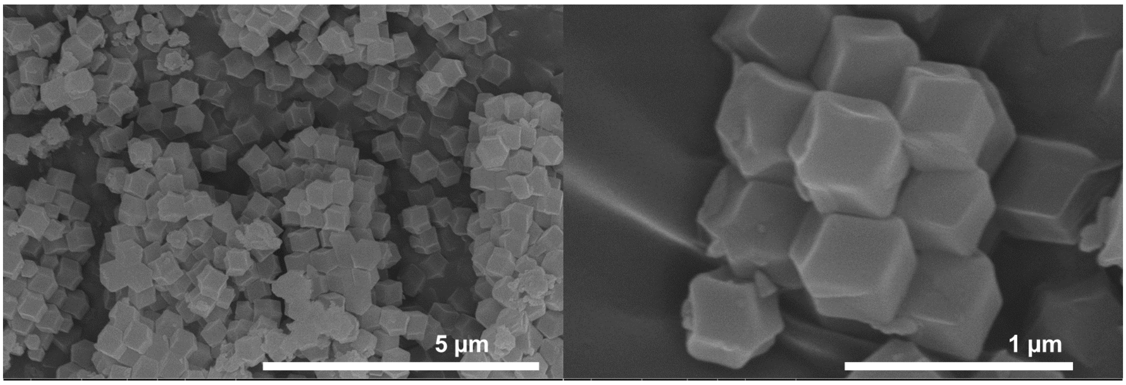

Figure S3. SEM images of evacuated pure ZIF-8 particles.

2. Synthesis and characterisation of 50(Na_2O)-50(P_2O_5)

Following a previously described methodology,[3] the raw materials Na_2CO_3 and $(NH_4)_2HPO_4$ were mixed in a ratio 1:1 to prepare a 10 g batch. Then this mixture was melted at 900 ºC for 1 hour in an alumina crucible and finally quenched to form homogeneous glassy sample. After quenching, the glass was annealed at 270 ºC for 30 minutes and allowed to cool to room temperature. Glass piece was pulverised in a Retsch PM 100 grinder at 350 rpm with 1 min intervals for half an hour using ZrO_2 balls (with roughly equal sample and ball volume). A bulk piece was saved from each composition to later be annealed for control measurements, such as elemental analysis and mechanical measurements.

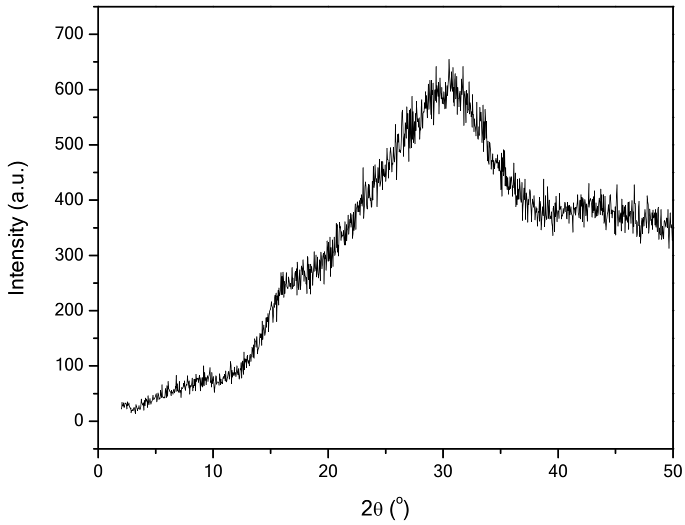

Figure S4. PXRD of 50(Na_2O)-50(P_2O_5) glass showing a typical amorphous pattern.

Table S1. EDS analysis summary for 50(Na_2O)-50(P_2O_5) glass.

**Nominal: 50(Na_2O)-50(P_2O_5) / Measured: 47(Na_2O)-53(P_2O_5)**			
	Na atomic %	P atomic %	O atomic %
**Nominal**	20.00	20.00	60.00
**Measured**	18.59 ± 0.15	19.83 ± 0.17	61.59 ± 0.14

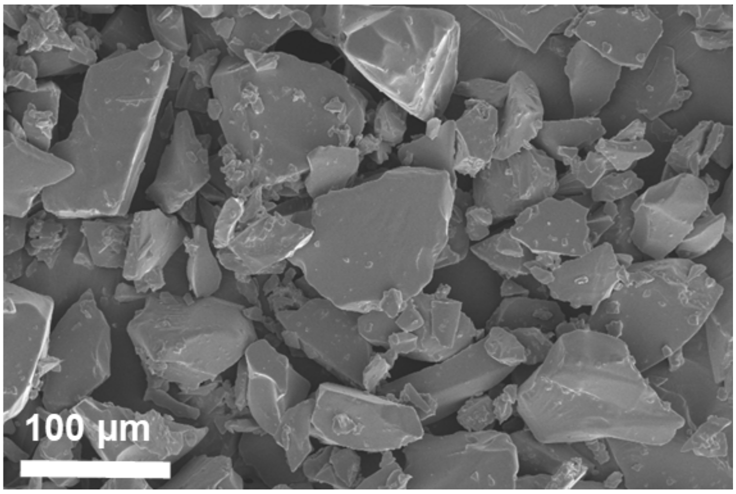

Figure S5. SEM image of 50(Na_2O)-50(P_2O_5) glass sample prior to any ball milling treatment.

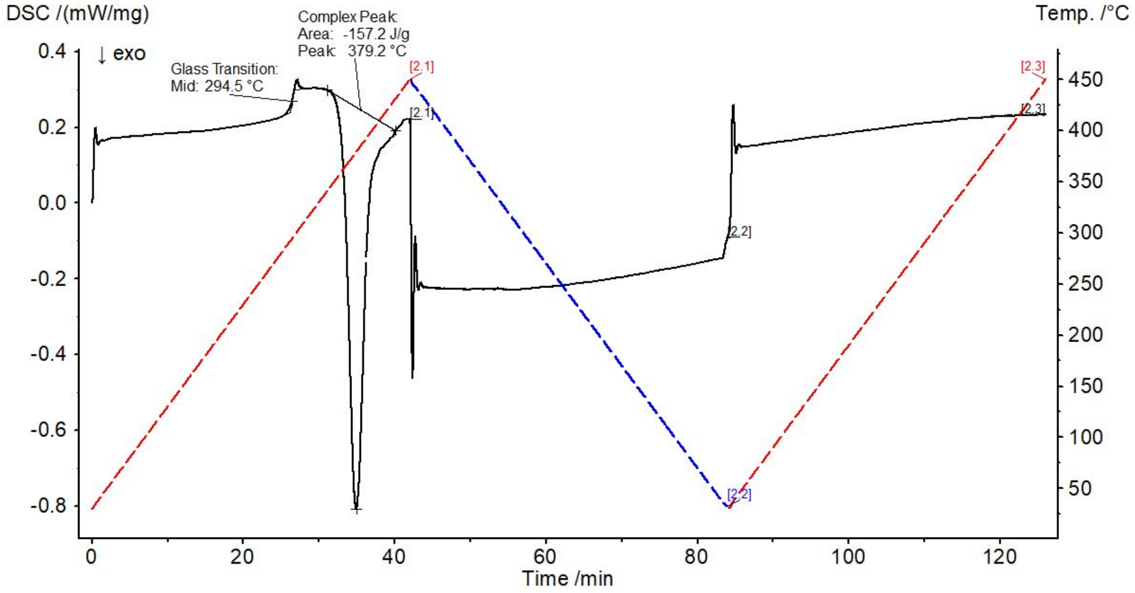

Figure S6. DSC of 50(Na_2O)50(P_2O_5) glass performed at a maximum temperature of 450 ^{o}C under an argon atmosphere with a heating/cooling rate of 10 ºC/min. The exothermic process at 350 ºC is clearly labelled.

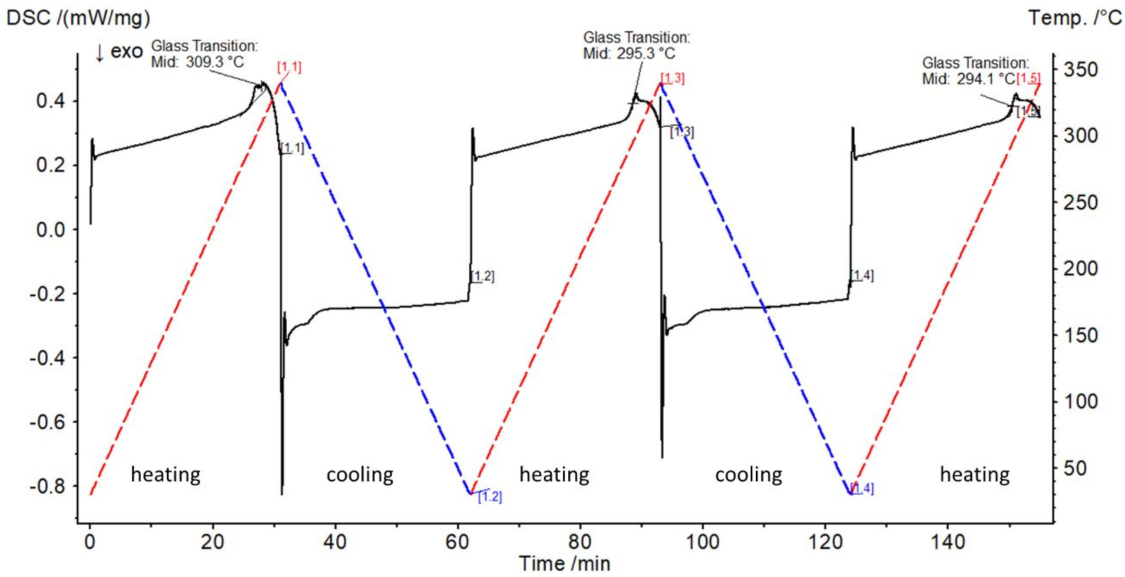

Figure S7. DSC heating/cooling cycles of 50(Na_2O)-50(P_2O_5) glass performed at a maximum temperature of 340 ºC under an argon atmosphere with a heating/cooling rate of 10 ºC/min.

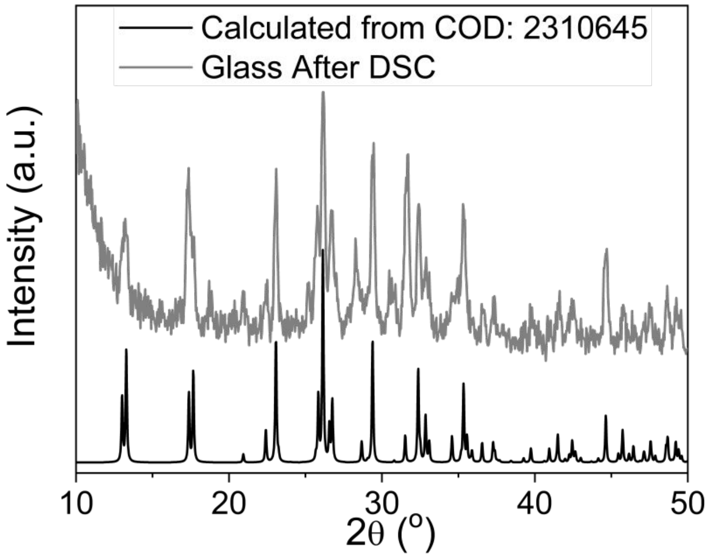

Figure S8. Identification of the $Na_3P_3O_9$ crystalline phase (COD: 2310645) by XRD of the glass after 450 ºC under Ar.

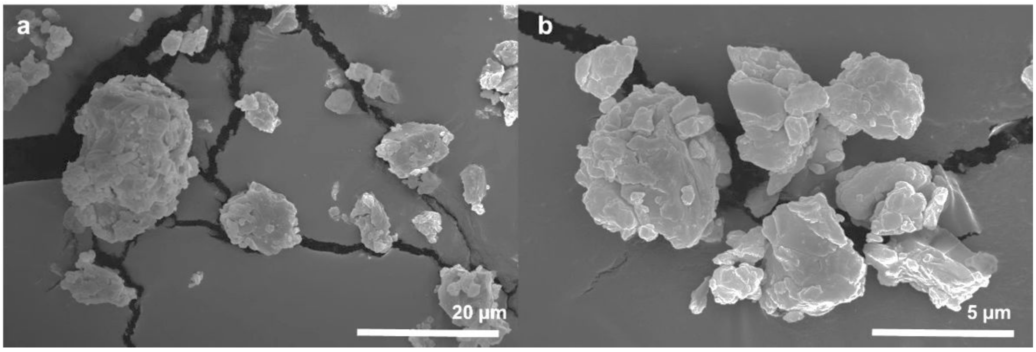

Figure S9. SEM images of $50(Na_2O)50(P_2O_5)$ glass after ball milling 30 min at 30 Hz (using two 5mm stainless steel balls).

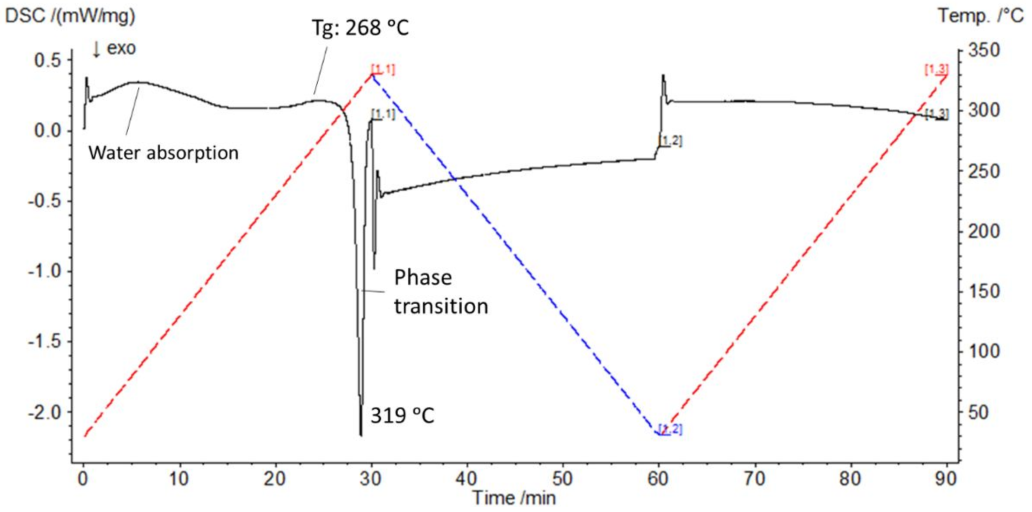

Figure S10. DSC of $50(Na_2O)50(P_2O_5)$ glass after 30 min ball-milling at 30 Hz Experiment performed at a maximum temperature of 450 ºC under an argon atmosphere with a heating/cooling rate of 10 ºC/min.

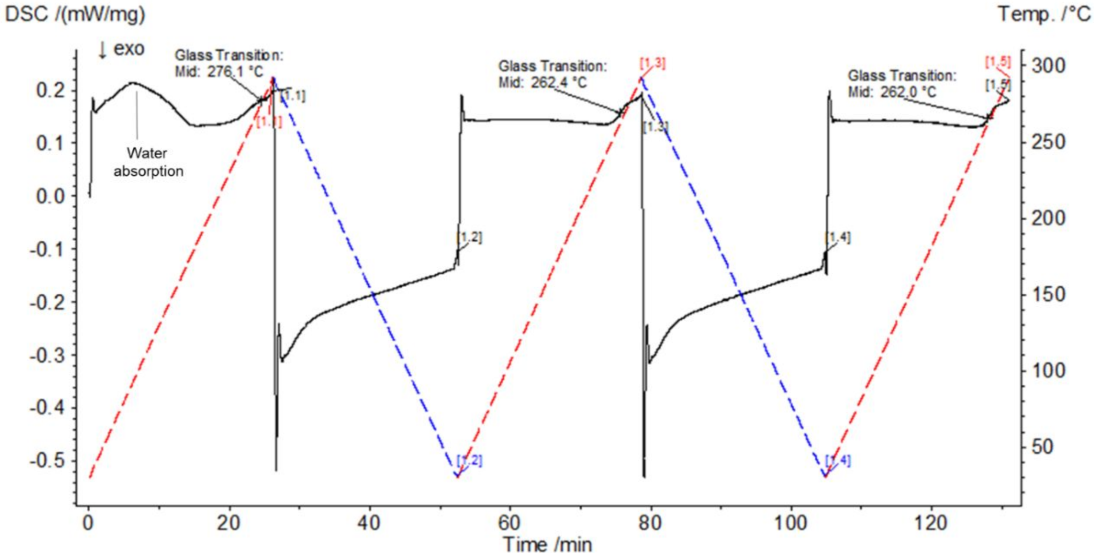

Figure S11. DSC of $50(Na_2O)50(P_2O_5)$ glass after 30 min ball-milling at 30 Hz. Experiment performed at a maximum temperature of 290 ºC under an argon atmosphere with a heating/cooling rate of 10 ºC/min.

3. Optimisation of the synthesis

General methodology. The previously activated ZIF-8 and ball milled glass were added into a 10 mL stainless steel jar in the appropriate weight ratios, to a total mass of 200 mg. The powders were mixed through ball milling with two 5 mm diameter stainless steel ball for 5 minutes at 20 Hz in a Retsch MM400 grinder mill. 150 mg samples of the ball-milled powder mixture (physical mixture) were pelletised at different pressures: 1, 0.74, 0.5 and 0.22 GPa, respectively, in order to optimise the synthesis. These pellets were placed in a Thermo Fisher vacuum furnace and heated to 310 ºC and held there for 30 minutes under dynamic vacuum.

- Optimisation of the pressure for the pellet formation

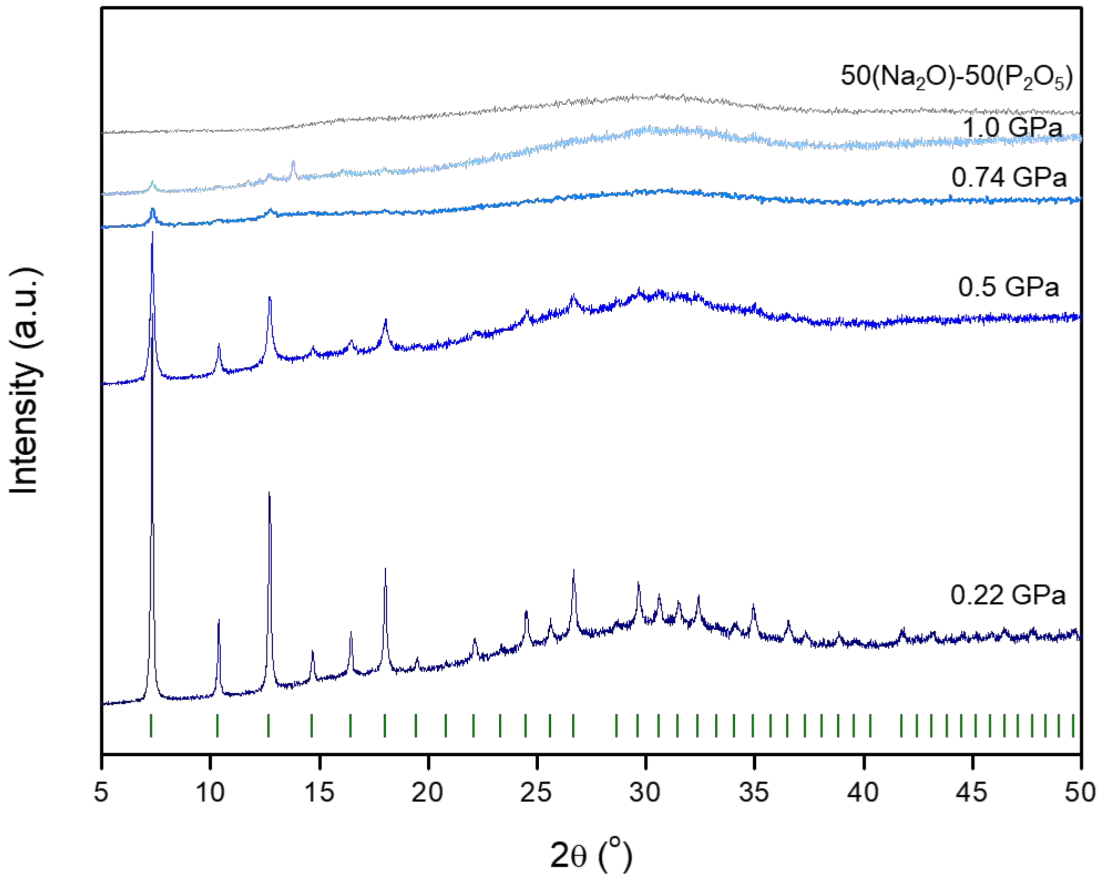

Figure S12. PXRD comparison of the MOF-CIGCs pelletised at different pressure values obtained after a thermal treatment at 310 ºC for 30 min. Bragg positions of the ZIF-8 structure were depicted as green bars at the bottom.

Table S2. CHN analyses calculated and found for the different compositions of $[(ZIF\text{-}8)_x(IG)_{1-x}]$ composites.

Composite	Formula	%C		%H		%N	
		Calculated	Found	Calculated	Found	Calculated	Found
$[(ZIF\text{-}8)_{0.3}(IG)_{0.7}]$	$C_{1.20}H_{1.50}N_{0.60}Na_{1.00}O_{3.00}P_{1.00}Zn_{0.15}$	10.49	11.09	1.11	1.17	6.18	6.47
$[(ZIF\text{-}8)_{0.2}(IG)_{0.8}]$	$C_{0.80}H_{1.00}N_{0.40}Na_{1}O_{3}P_{1}Zn_{0.10}$	7.70	7.26	0.81	0.70	4.49	4.67
$[(ZIF\text{-}8)_{0.1}(IG)_{0.9}]$	$C_{0.40}H_{0.50}N_{0.20}Na_{1.00}O_{3.00}P_{1.00}Zn_{0.05}$	4.24	4.36	0.44	0.31	2.47	2.36

4. Liquid-state Nuclear Magnetic Resonance

Liquid-state ^{1}H NMR spectra of the (ZIF-8)$_{0.3}$(IG)$_{0.7}$ physical mixture and the [(ZIF-8)$_{0.3}$(IG)$_{0.7}$] composite, digested in acidic media, reveal signals at 2.40 ppm and 7.24 ppm which are assigned to the C$\boldsymbol{H}_3$ and C$\boldsymbol{H}$ protons in mIm (2-mehtylimidazolate), respectively. The additional signal at 8.13 ppm is assigned to residual H_3O^+, as already discussed in the literature.[4] A similar signal, although at a slightly higher chemical shift of ~8.28 ppm, is also observed for the 50(Na_2O)-50(P_2O_5) inorganic glass. Acidic protons (-N***H***) are believed to undergo rapid exchange with $(H,D)_3O^+$, similarly to what has been observed for ZIF-8 digested using the same experimental procedure.[4] The relative area of the signals assigned to the C$\boldsymbol{H}_3$ and C***H*** protons is 2.9 : 2.0 for (ZIF-8)$_{0.3}$(IG)$_{0.7}$ and 3.0 : 2.0 for [(ZIF-8)$_{0.3}$(IG)$_{0.7}$], in excellent agreement with the 3:2 theoretical ratio.

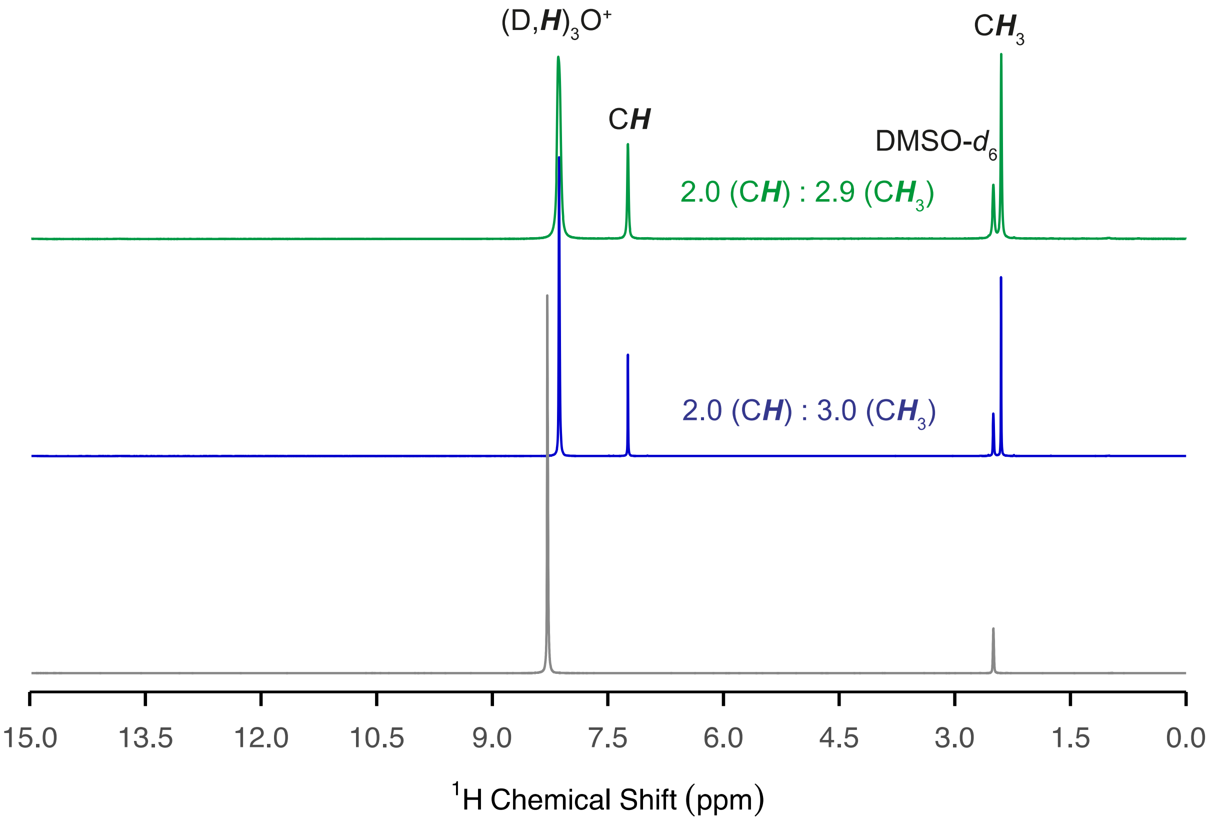

Figure S13. ^{1}H liquid-state NMR spectra of the (ZIF-8)$_{0.3}$(IG)$_{0.7}$ physical mixture (green), [(ZIF-8)$_{0.3}$(IG)$_{0.7}$] composite (blue) and 50(Na_2O)-50(P_2O_5) inorganic glass (grey) after digestion recorded at 9.4 T using a recycle delay of 10 s. The spectral assignment (black text) and the relative area of the C$\boldsymbol{H}_3$ and C***H*** signals observed for (ZIF-8)$_{0.3}$(IG)$_{0.7}$ and [(ZIF-8)$_{0.3}$(IG)$_{0.7}$] (coloured text) are highlighted. The residual protonated signal of DMSO-d_6 is also given in the figure.

5. Powder X-ray diffraction

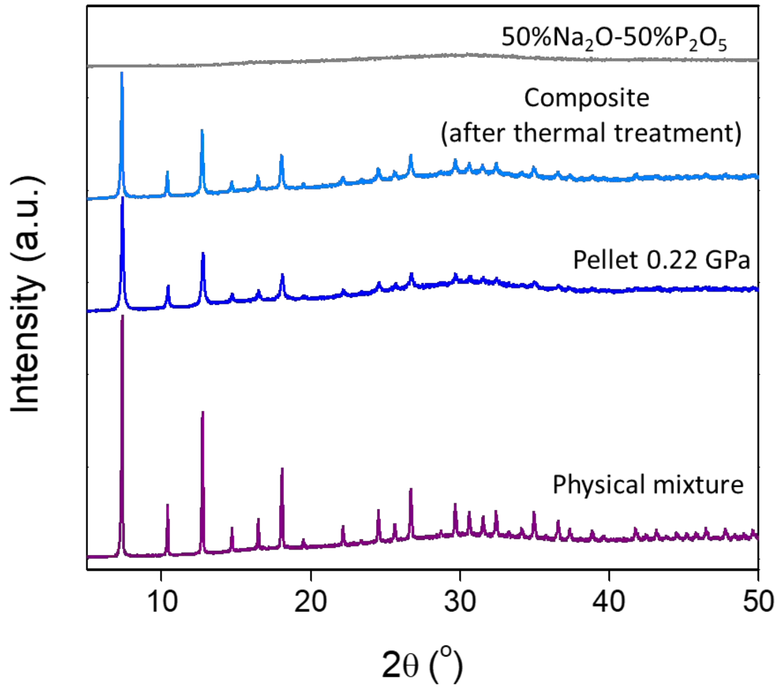

Figure S14. PXRD comparison of the material prepared with weight ratios 80/20: physical mixture (purple), after the pelletisation at 0.22 GPa for 1 min (blue), after the thermal treatment at 310 ºC for 30 min (light blue) and the inorganic glass (grey).

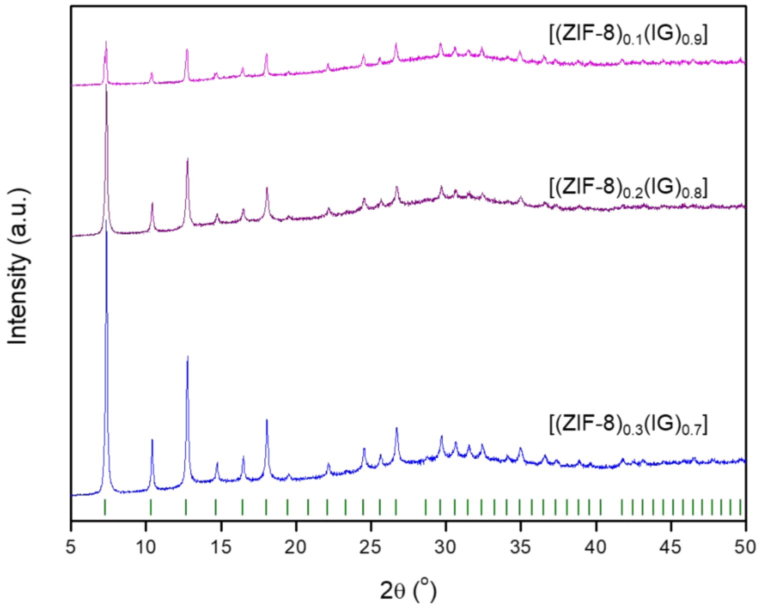

Figure S15. PXRD comparison of the compositional series of physical mixtures pelletised at 0.22 GPa obtained after a thermal treatment at 310 ºC for 30 min. Bragg positions of the ZIF-8 structure were depicted as green bars at the bottom.

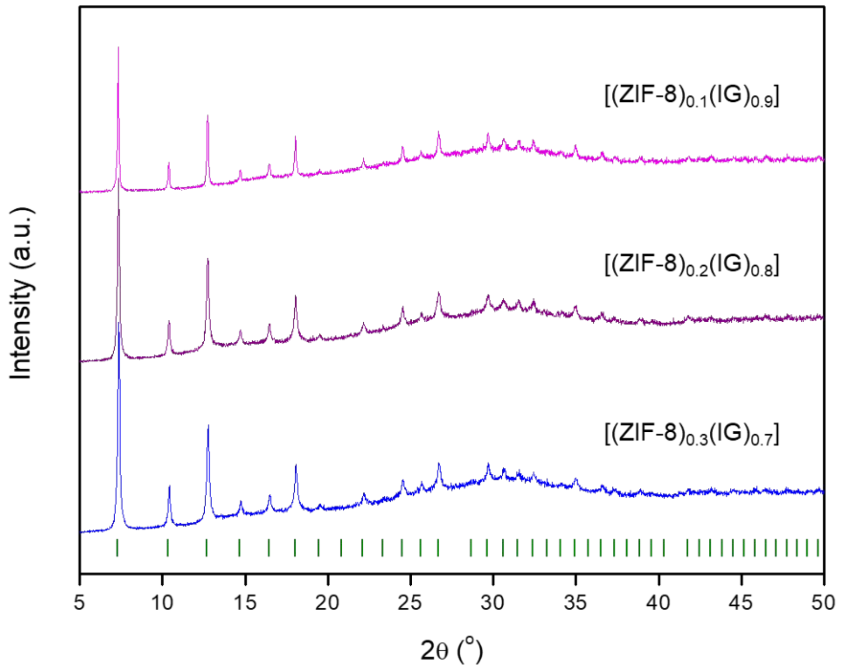

Figure S16. PXRD comparison of the compositional series of MOF-CIGCs pelletised at 0.22 GPa obtained after a thermal treatment at 310 ºC for 30 min. Bragg positions of the ZIF-8 structure were depicted as green bars at the bottom.

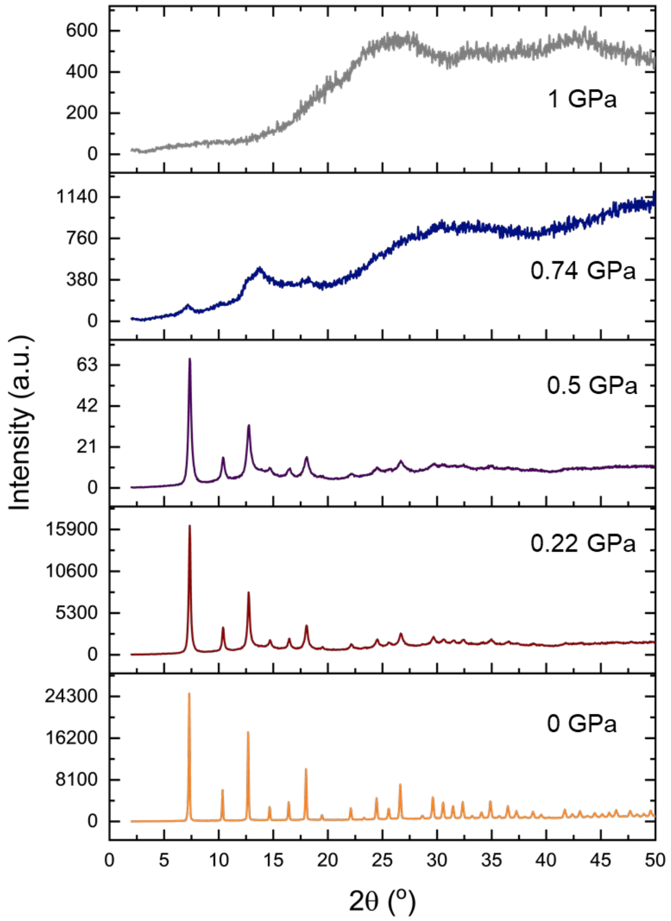

Figure S17. PXRD comparison of ZIF-8 pelletised at different pressure values.

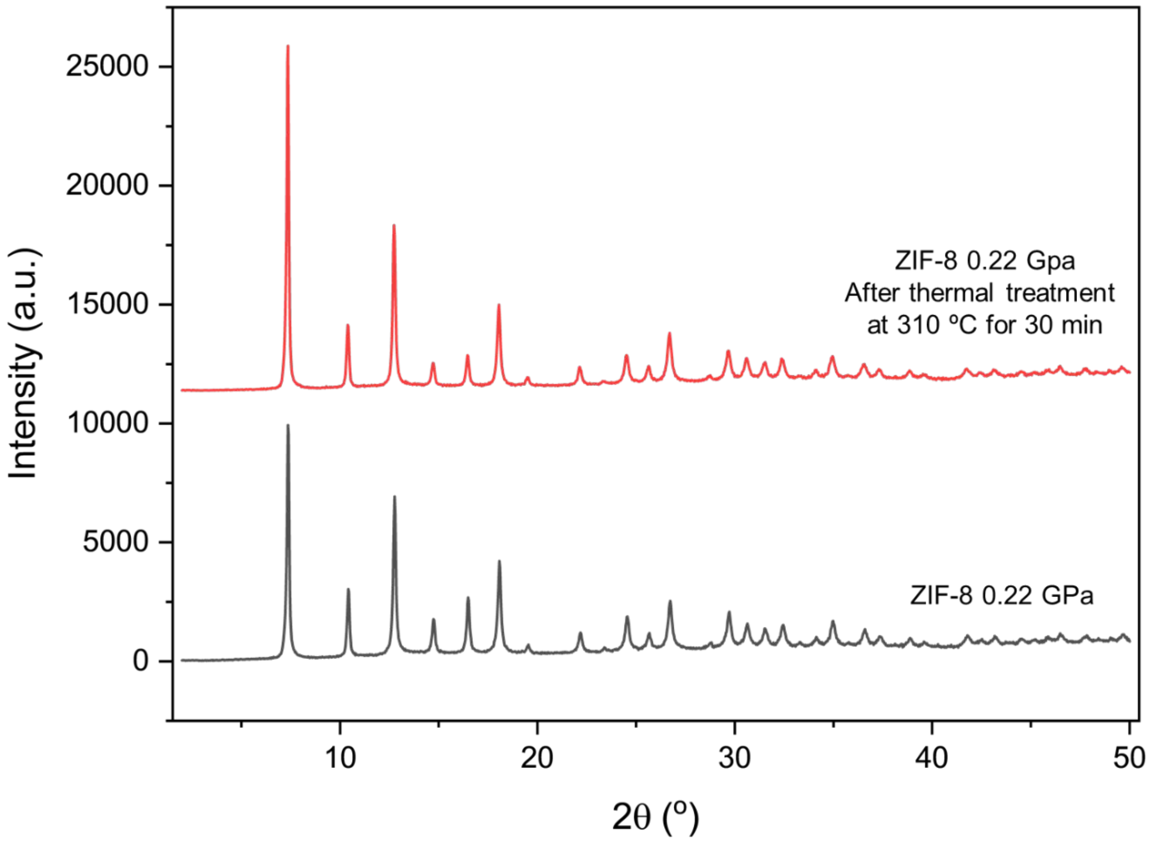

Figure S18. PXRD comparison of ZIF-8 pelletised at 0.22 GPa before (grey) and after a thermal treatment at 310 ºC for 30 min under vacuum (red).

Table S3. Peak parameters comparison for the first peak (110) of ZIF-8 pristine and the [(ZIF-8)$_{0.2}$(IG)$_{0.8}$] composite at 0.22 GPa of pressure before and after a thermal treatment at 310 ºC for 30 minutes.

**Sample**	**ZIF-8 0.22 GPa**	**ZIF-8 0.22 GPa after heating**	**[(ZIF-8)$_{0.2}$(IG)$_{0.8}$] pellet**	**[(ZIF-8)$_{0.2}$(IG)$_{0.8}$] pellet after heating**
Peak position (2θ)	7.36507	7.35933	7.35852	7.34985
Height (a.u.)	9874.2	13596.1	5441.03	5798.64
Area (a.u.)	1927.76	2483.35	1248.56	896.69

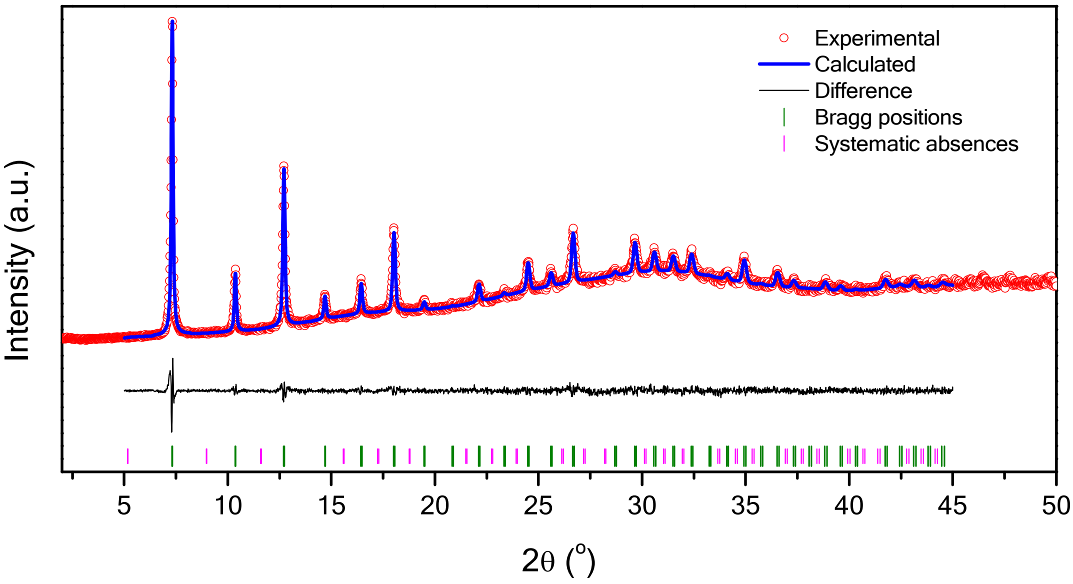

Figure S19. Pawley refinement of [$(ZIF\text{-}8)_{0.3}(IG)_{0.7}$] composite (after pelletisation and thermal treatment). Initial parameters were obtained from the CIF file previously reported for crystalline ZIF-8.[2]

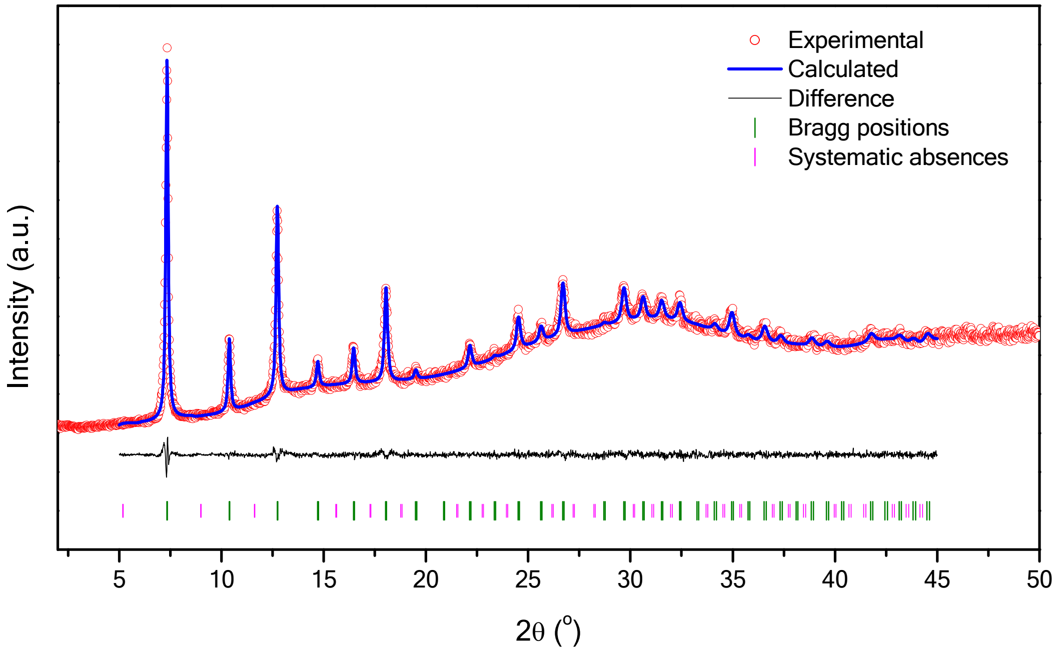

Figure S20. Pawley refinement of [$(ZIF\text{-}8)_{0.2}(IG)_{0.8}$] composite (after pelletisation and thermal treatment). Initial parameters were obtained from the CIF file previously reported for crystalline ZIF-8.[2]

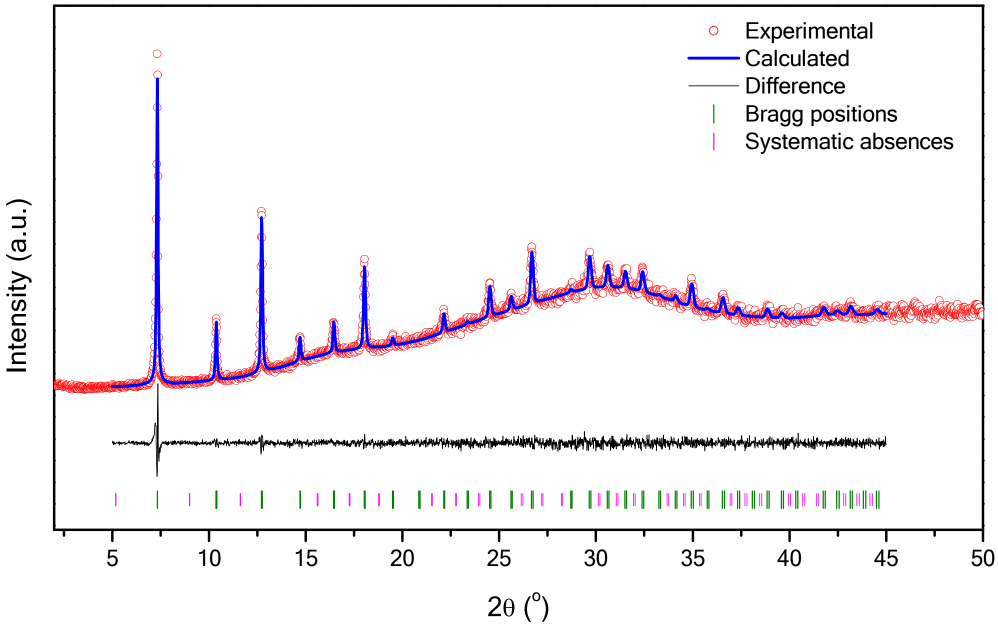

Figure S21. Pawley refinement of [(ZIF-8)$_{0.1}$(IG)$_{0.9}$] composite (after pelletisation and thermal treatment). Initial parameters were obtained from the CIF file previously reported for crystalline ZIF-8.[2]

Table S4. Pawley refinement details

Composite	R_{wp}	Space group	Zero	Lattice parameter / Å	Profile parameters	Lattice parameter reported from[2]	Lattice parameter found in the ZIF-8 pristine
[(ZIF-8)$_{0.3}$(IG)$_{0.7}$]	4.27 %	*I-43m*	0.0091(21)	17.004(24)	U=0.24(5)<br>V=-0.0092(13)<br>W= 0.0051(7)	16.8509(3) Å	17.3525(12) Å
[(ZIF-8)$_{0.2}$(IG)$_{0.8}$]	3.70 %	*I-43m*	-0.0046 (12)	17.0198(18)	U=0.37(12)<br>V=-0.08(2)<br>W= 0.007(1)		
[(ZIF-8)$_{0.1}$(IG)$_{0.9}$]	4.28 %	*I-43m*	0.0015(2)	17.010(24)	U=0.16(5)<br>V=-0.023(12)<br>W= 0.0041(7)		

6. Thermal characterisation

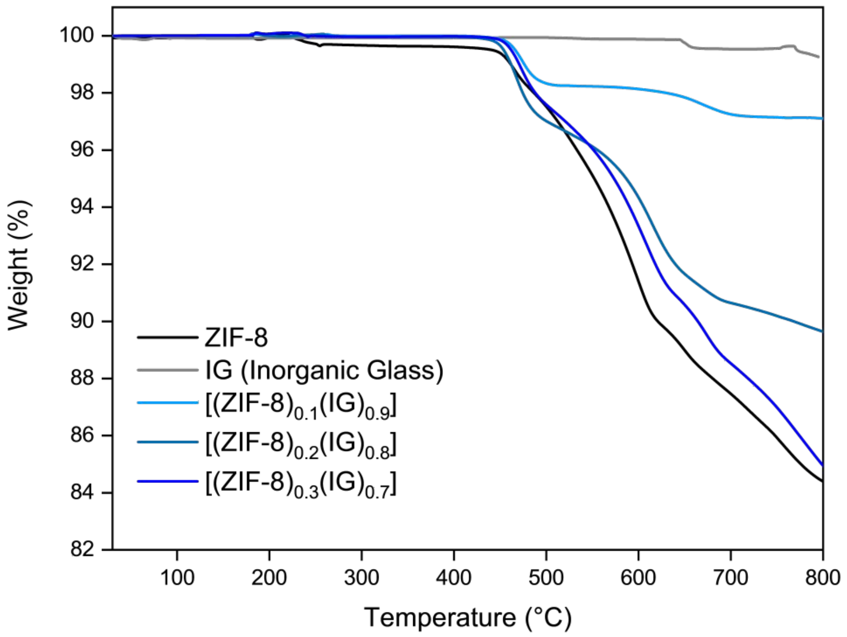

Figure S22. TGA of the synthesised MOF-CIGCs, ZIF-8 and the inorganic glass.

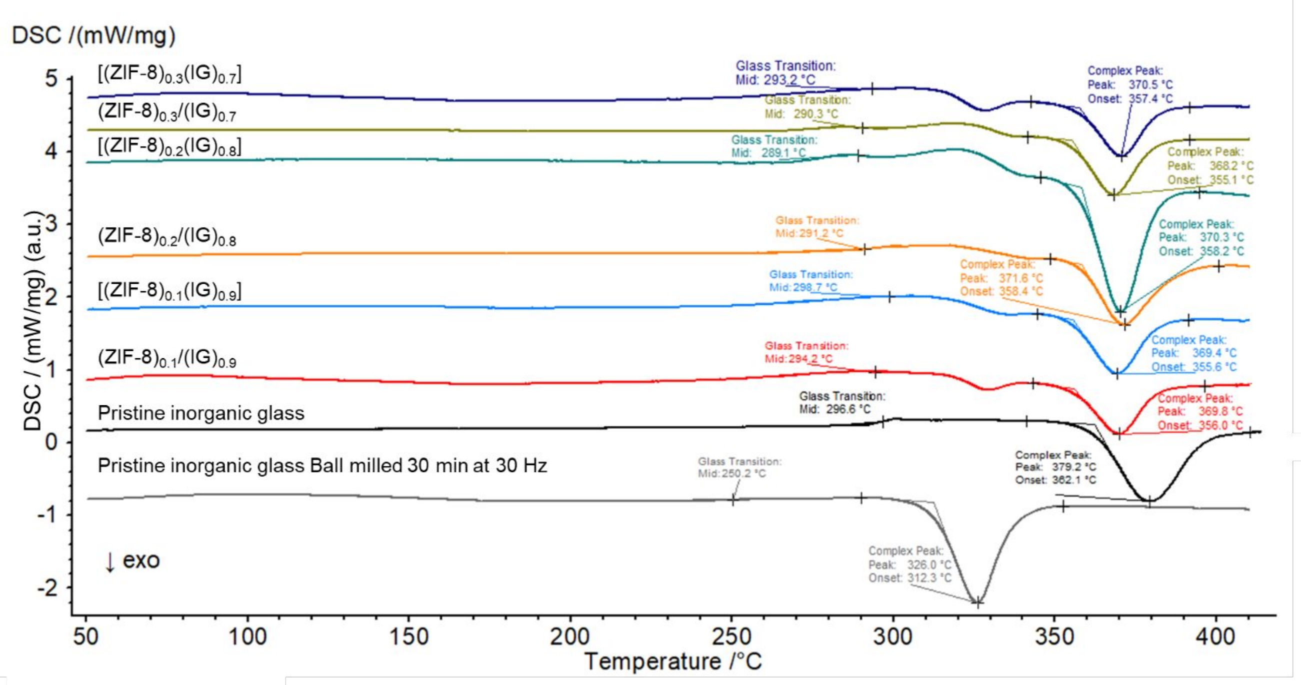

Figure S23. DSC curves for all the physical mixtures, composites, inorganic glass pristine and inorganic glass ball milled 30 min at 30 Hz performed at a maximum temperature of 420 ºC under an argon atmosphere with a heating/cooling rate of 10 ºC/min.

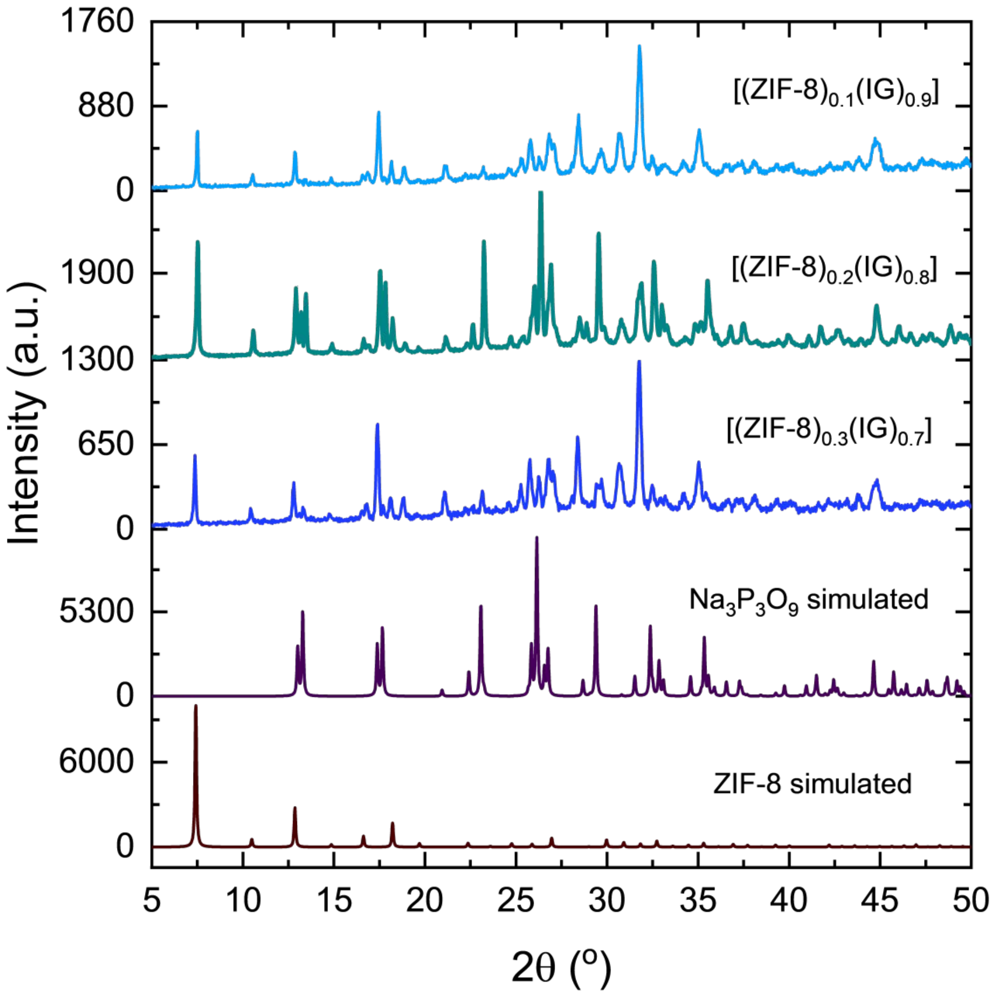

Figure S24. Normalised PXRD patterns of simulated ZIF-8 (maroon) and $Na_3P_3O_9$ (violet) materials and $[(ZIF\text{-}8)_{0.3}(IG)_{0.7}]$ (blue), $[(ZIF\text{-}8)_{0.2}(IG)_{0.8}]$ (turquoise) and $[(ZIF\text{-}8)_{0.1}(IG)_{0.9}]$ (cyan) composites after DSC experiment at 420 ºC under argon flow and a 10 ºC/min heating ramp.

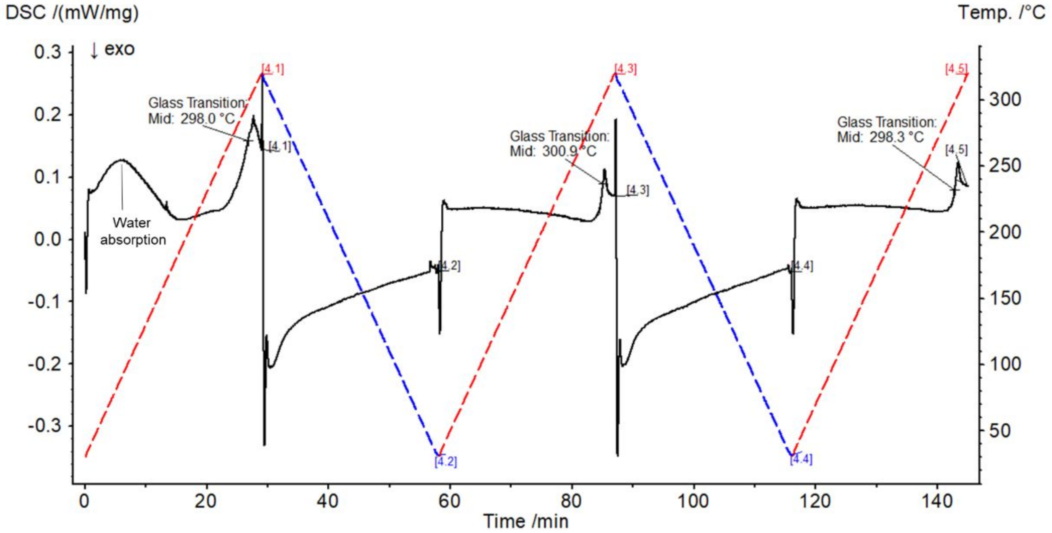

Figure S25. DSC of $(ZIF\text{-}8)_{0.1}/(IG)_{0.9}$ physical mixture performed at a maximum temperature of 320 ºC under an argon atmosphere with a heating/cooling rate of 10 ºC/min.

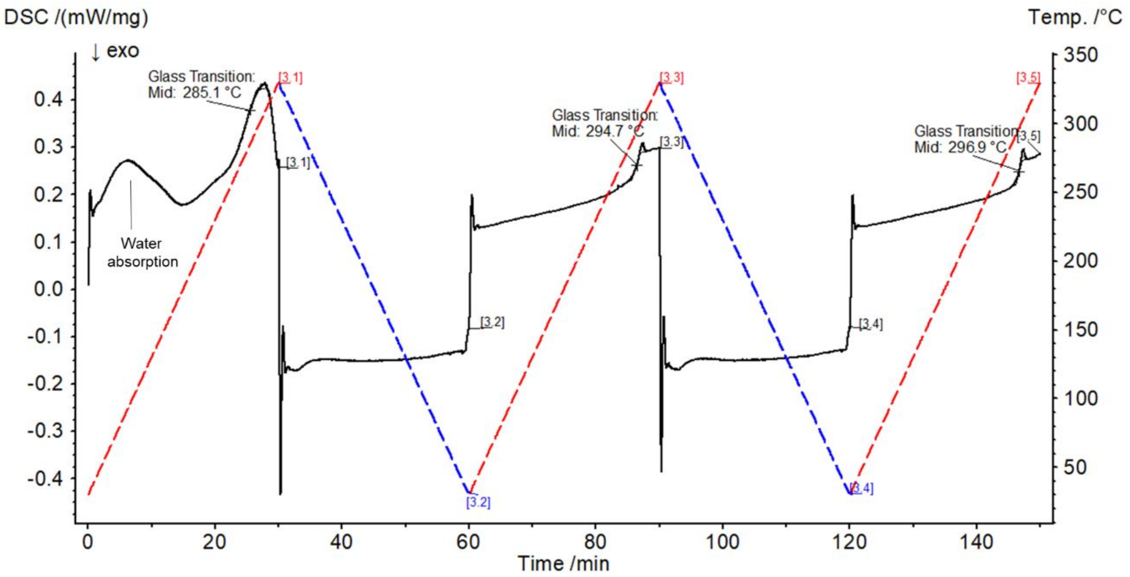

Figure S26. DSC of $(ZIF\text{-}8)_{0.2}/(IG)_{0.8}$ physical mixture performed at a maximum temperature of 320 ºC under an argon atmosphere with a heating/cooling rate of 10 ºC/min.

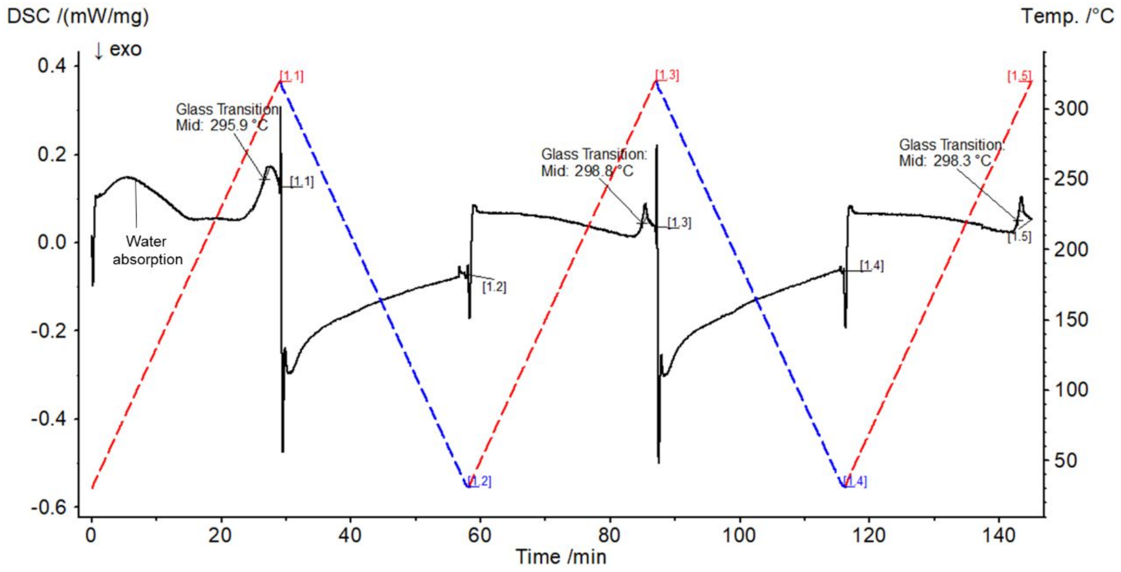

Figure S27. DSC of $(ZIF\text{-}8)_{0.3}/(IG)_{0.7}$ physical mixture performed at a maximum temperature of 320 ºC under an argon atmosphere with a heating/cooling rate of 10 ºC/min.

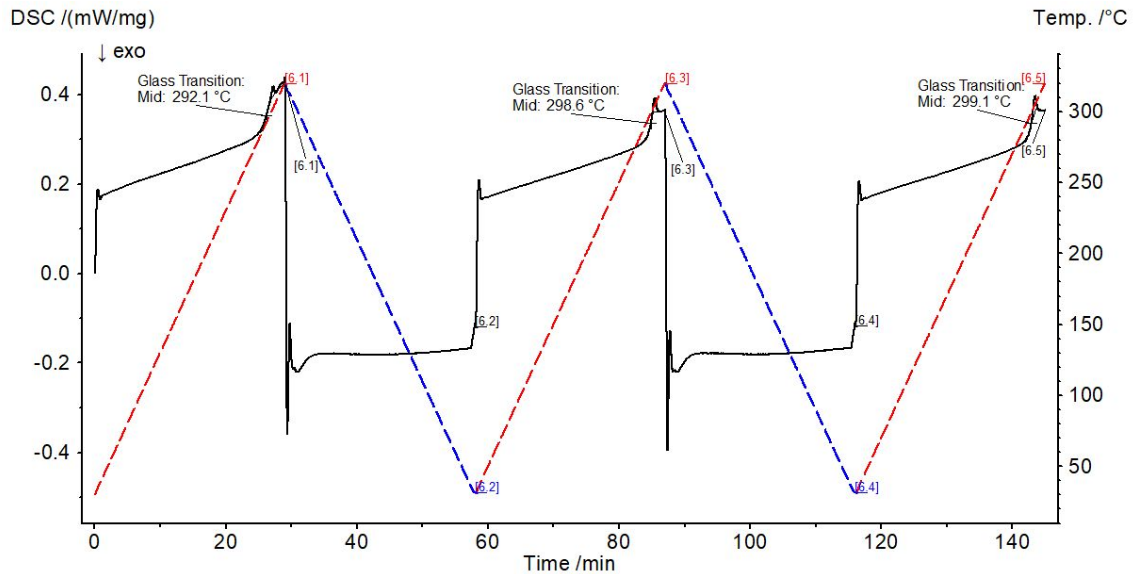

Figure 28. DSC of [(ZIF-8)$_{0.1}$(IG)$_{0.9}$] composite performed at a maximum temperature of 320 ºC under an argon atmosphere with a heating/cooling rate of 10 ºC/min.

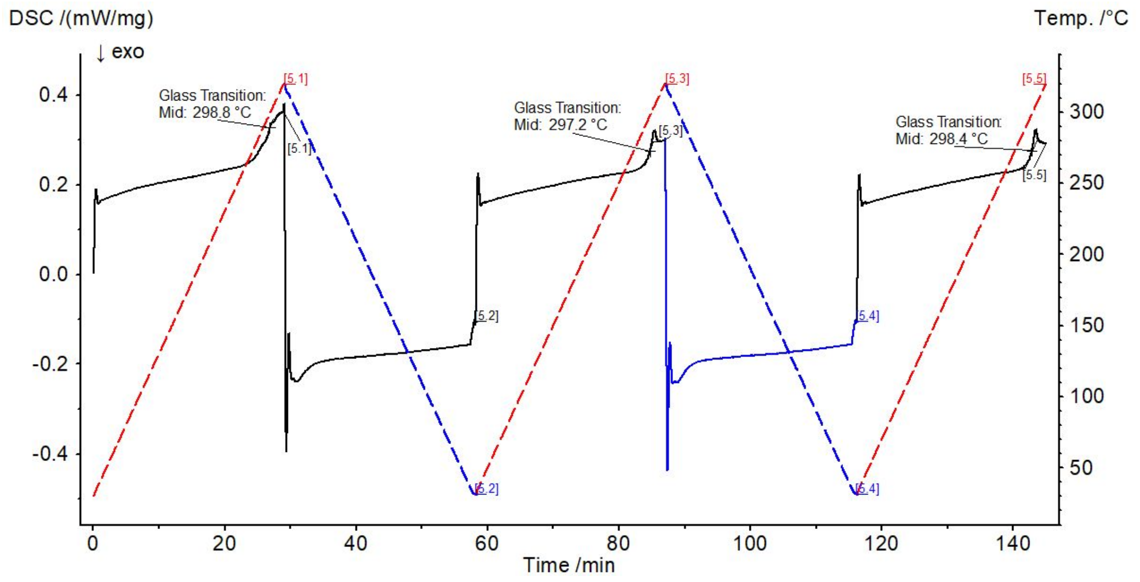

Figure S29. DSC of [(ZIF-8)$_{0.2}$(IG)$_{0.8}$] composite performed at a maximum temperature of 320 ºC under an argon atmosphere with a heating/cooling rate of 10 ºC/min.

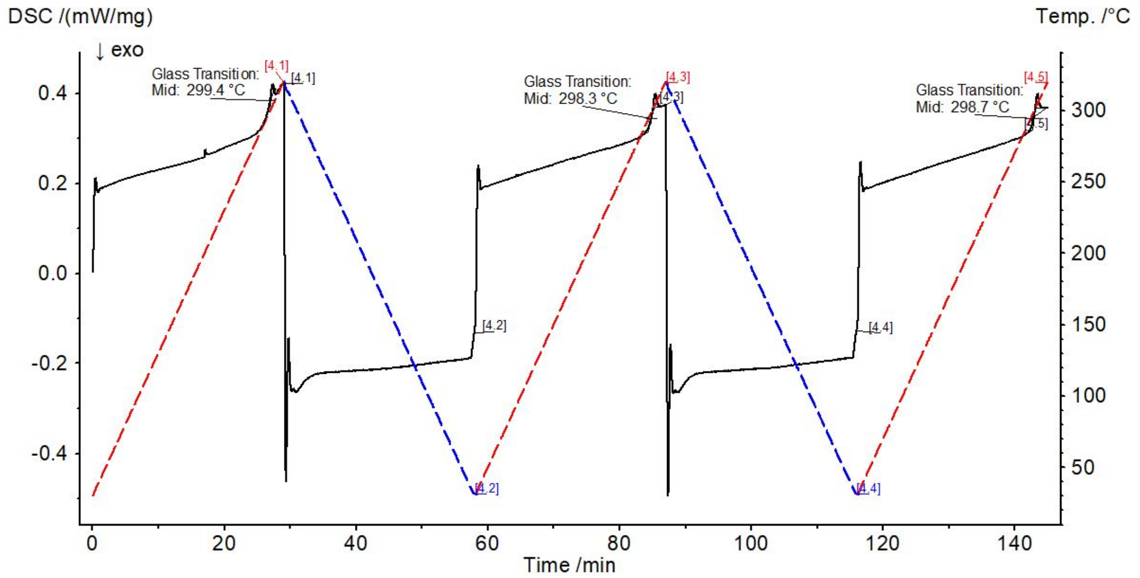

Figure S30. DSC of [(ZIF-8)$_{0.3}$(IG)$_{0.7}$] composite performed at a maximum temperature of 320 ºC under an argon atmosphere with a heating/cooling rate of 10 ºC/min.

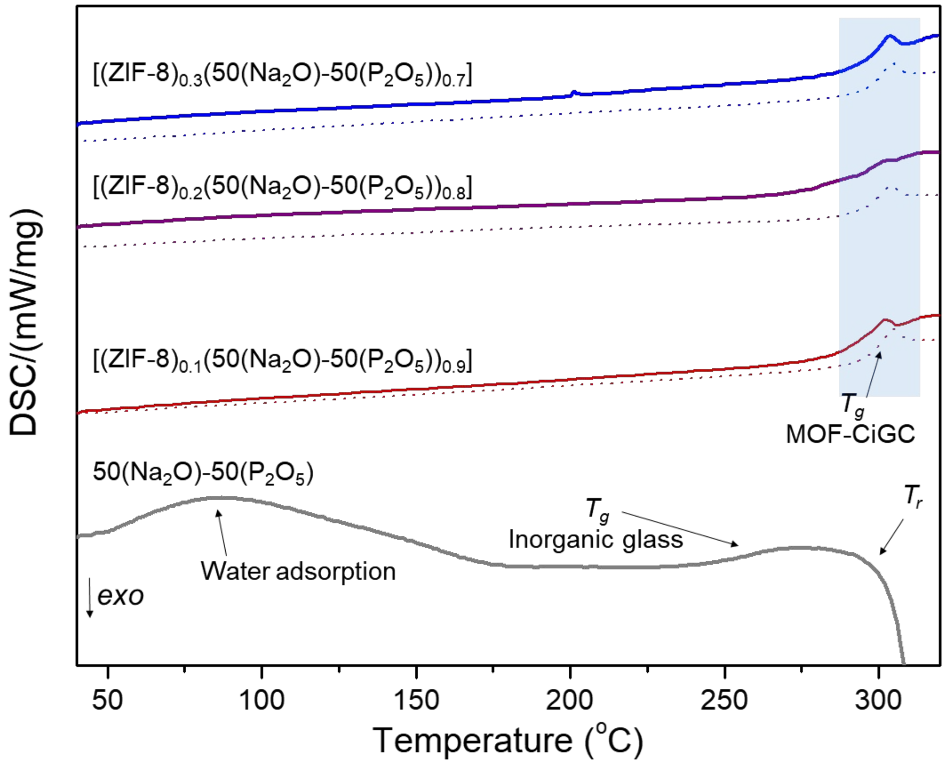

Figure S31. Comparison of the DSC profiles between composites and the inorganic glass ball milled (grey). 2nd upscans for composites are depicted using dotted lines.

7. Scanning electron microscopy study

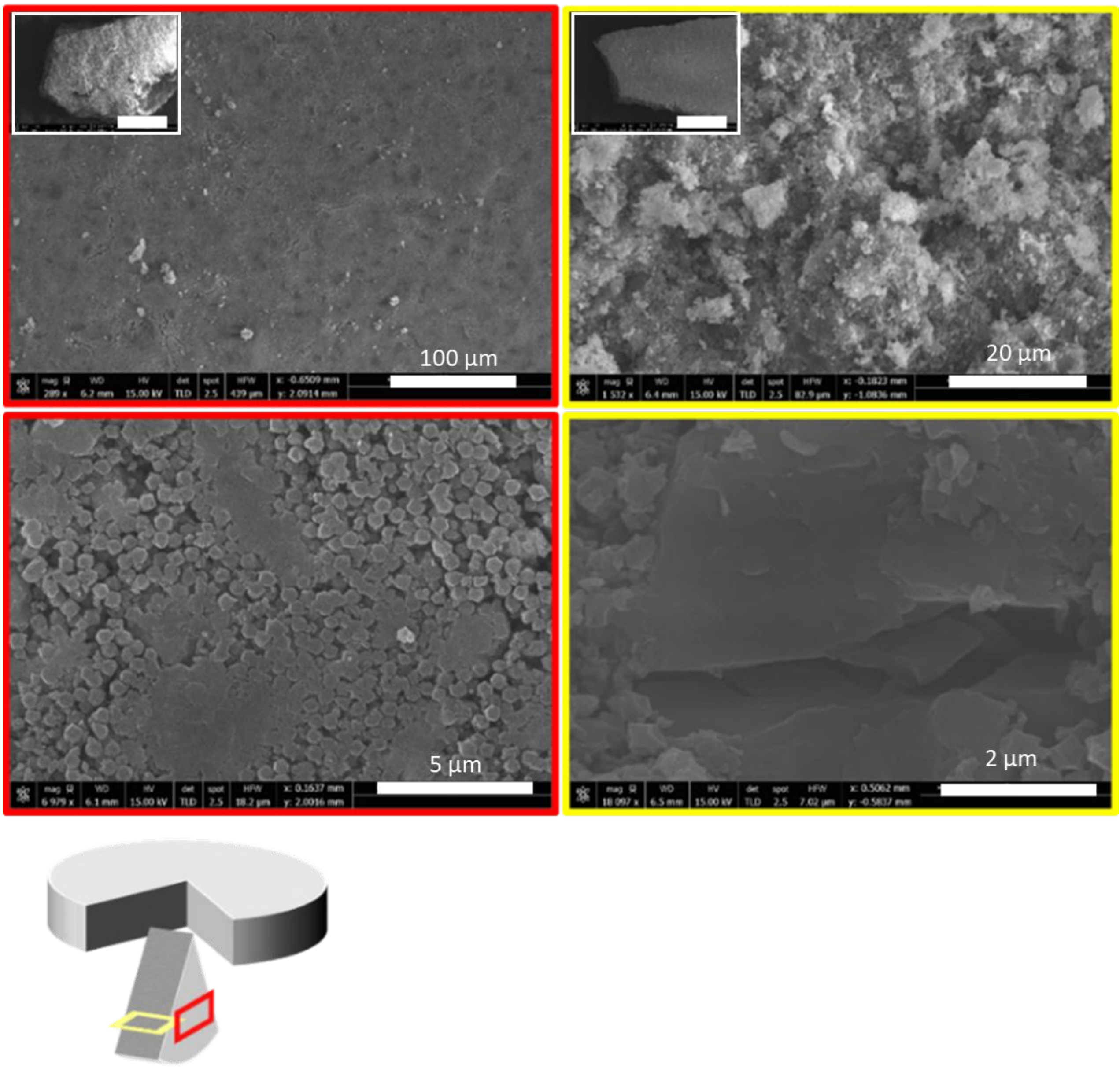

Figure S32. Scheme of a pellet piece of [$(ZIF\text{-}8)_{0.3}(IG)_{0.7}$] composite where SEM images with red edge were taken from the surface of the pellet. Yellow-edge images from inside the pellet showing ZIF-8 crystallites are embedded by the inorganic glass matrix. White bars in the insets have a length of 1 mm.

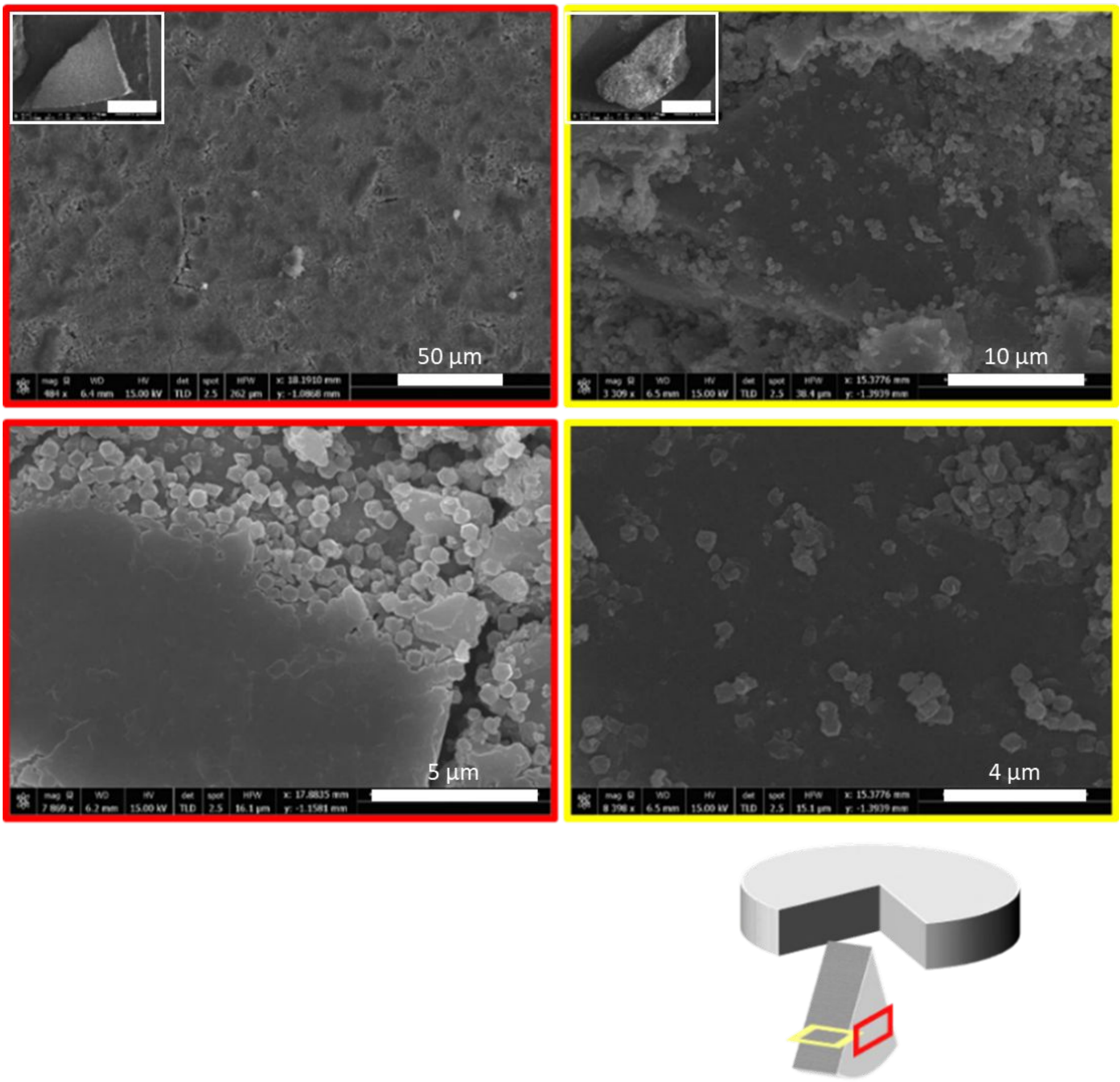

Figure S33. Scheme of a pellet piece of [(ZIF-8)$_{0.1}$(IG)$_{0.9}$] composite where SEM images with red edge were taken from the surface of the pellet. Yellow-edge images from inside the pellet showing ZIF-8 crystallites are embedded by the inorganic glass matrix. White bars in the insets have a length of 1 mm.

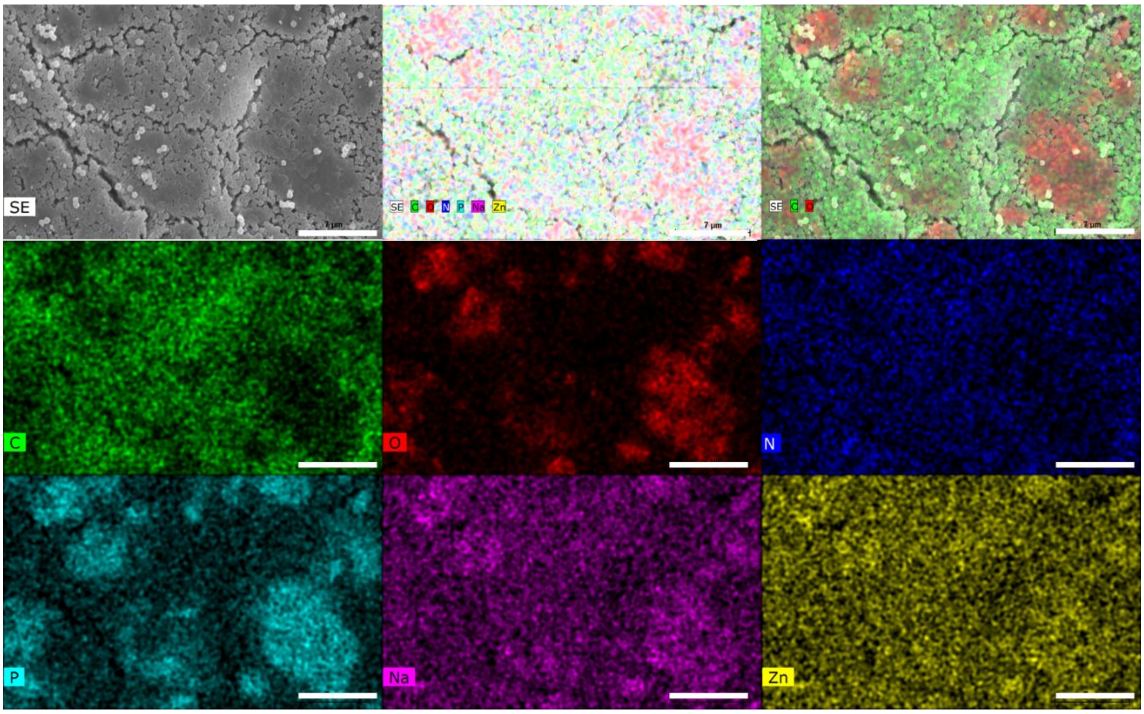

Figure S34. Mapping of surface of the composite pellet [(ZIF-8)$_{0.3}$(IG)$_{0.7}$]. First raw left to right: Image of the analysed area, all individual elements mappings, carbon and oxygen map. Carbon is related with the amount of ZIF-8 and oxygen with the amount of glass. Second and third raw individual map per element: C (green), O (red), N (blue), P (cyan), Na (pink), Zn (yellow). Scale 7 μm (white bar).

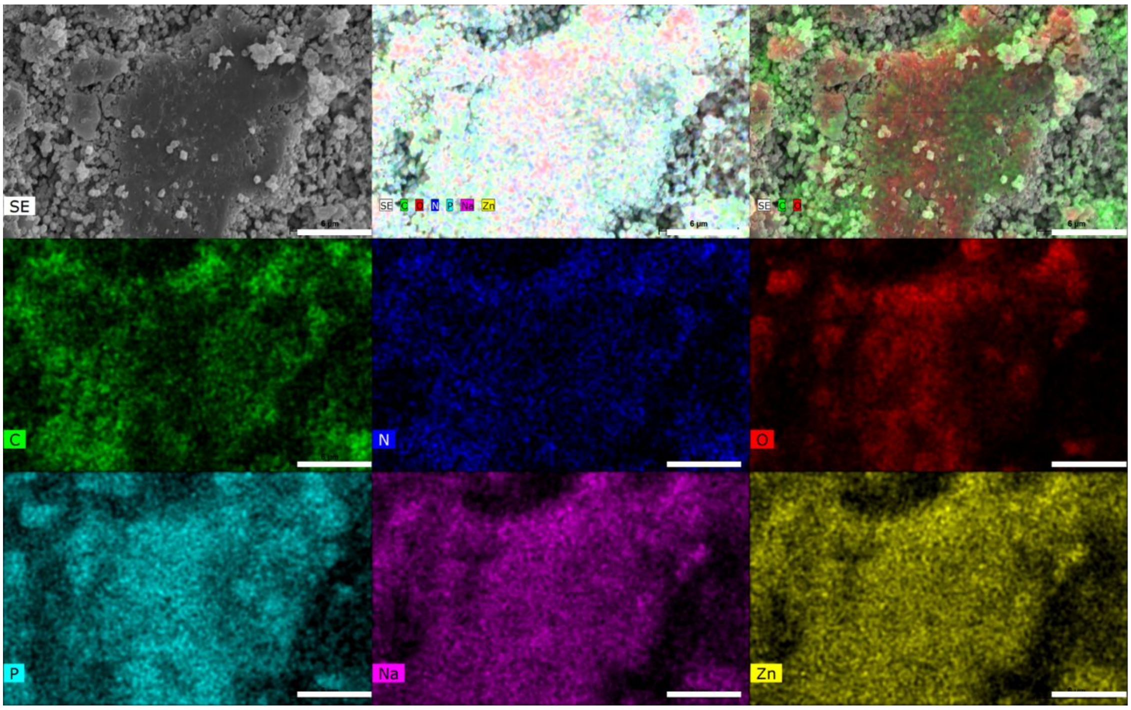

Figure S35. Mapping of surface of the composite pellet [(ZIF-8)$_{0.2}$(IG)$_{0.8}$]. First raw left to right: Image of the analysed area, all individual elements mappings, carbon and oxygen map. Carbon is related with the amount of ZIF-8 and oxygen with the amount of glass. Second and third raw individual map per element: C (green), O (red), N (blue), P (cyan), Na (pink), Zn (yellow). Scale 7 μm (white bar).

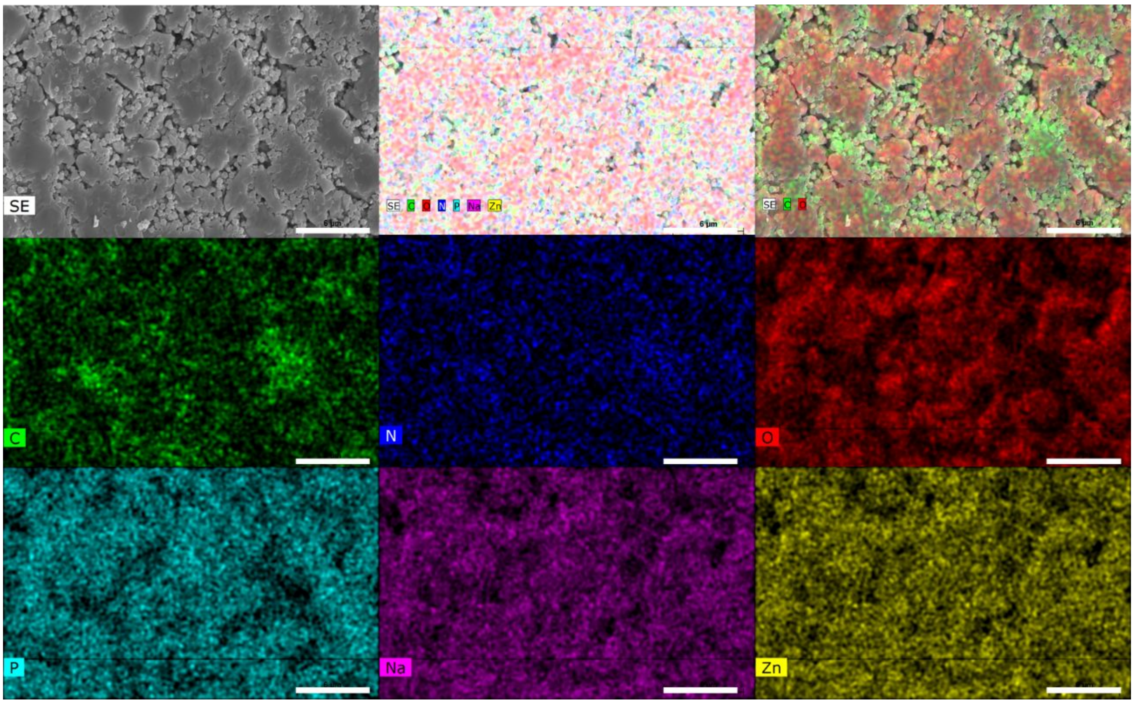

Figure S36. Mapping of surface of the composite pellet [$(ZIF\text{-}8)_{0.1}(IG)_{0.9}$]. First raw left to right: Image of the analysed area, all individual elements mappings, carbon and oxygen map. Carbon is related with the amount of ZIF-8 and oxygen with the amount of glass. Second and third raw individual map per element: C (green), O (red), N (blue), P (cyan), Na (pink), Zn (yellow). Scale 7 µm (white bar).

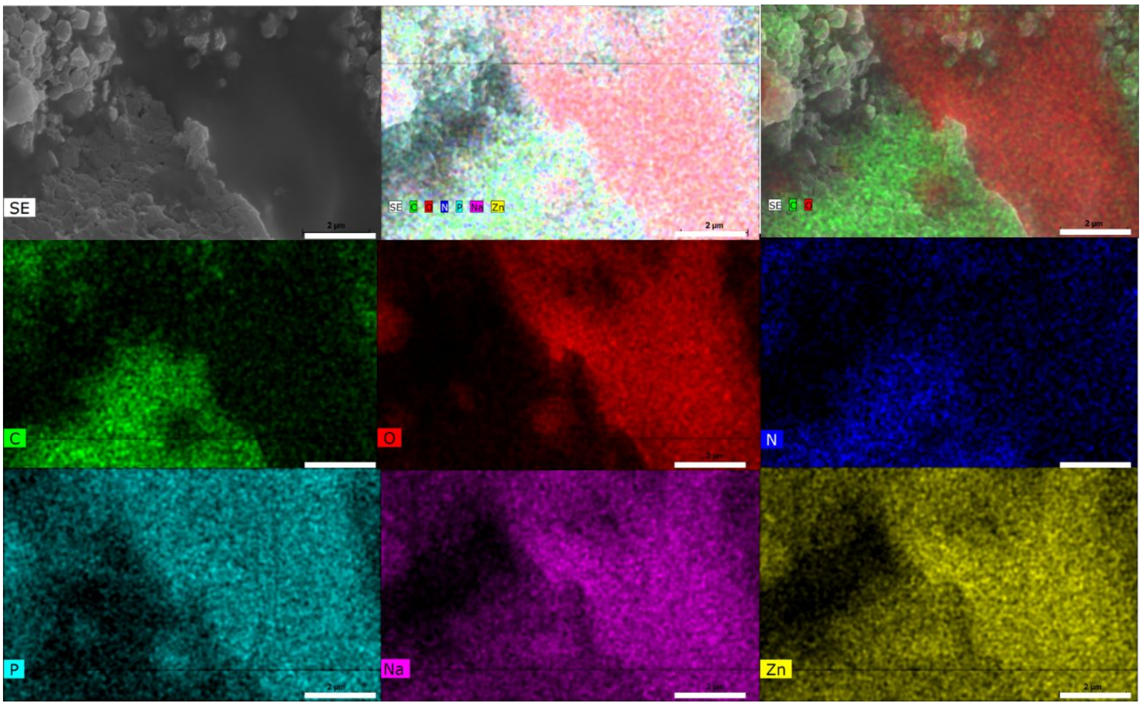

Figure S37. Mapping of inside of the composite pellet [$(ZIF\text{-}8)_{0.3}(IG)_{0.7}$]. First raw left to right: Image of the analysed area, all individual elements mappings, carbon and oxygen map. Carbon is related with the amount of ZIF-8 and oxygen with the amount of glass. Second and third raw individual map per element: C (green), O (red), N (blue), P (cyan), Na (pink), Zn (yellow). Scale 7 µm (white bar).

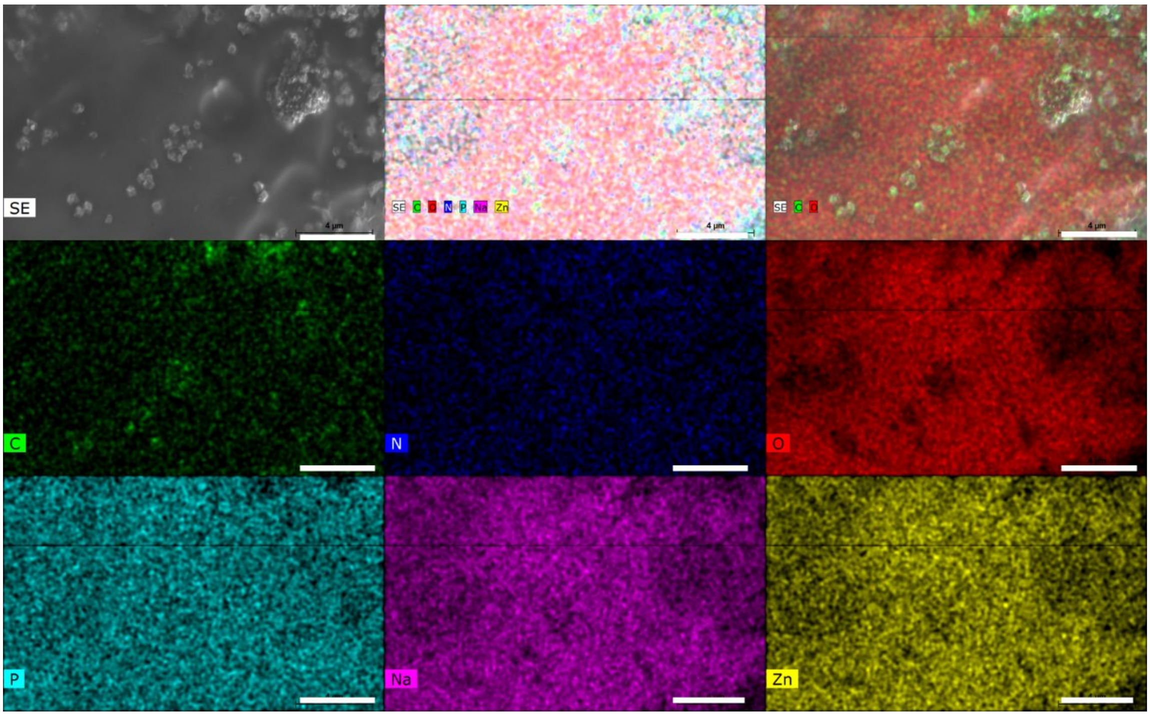

Figure S38. Mapping of inside of the composite pellet [$(ZIF\text{-}8)_{0.2}(IG)_{0.8}$]. First raw left to right: Image of the analysed area, all individual elements mappings, carbon and oxygen map. Carbon is related with the amount of ZIF-8 and oxygen with the amount of glass. Second and third raw individual map per element: C (green), O (red), N (blue), P (cyan), Na (pink), Zn (yellow). Scale 7 µm (white bar).

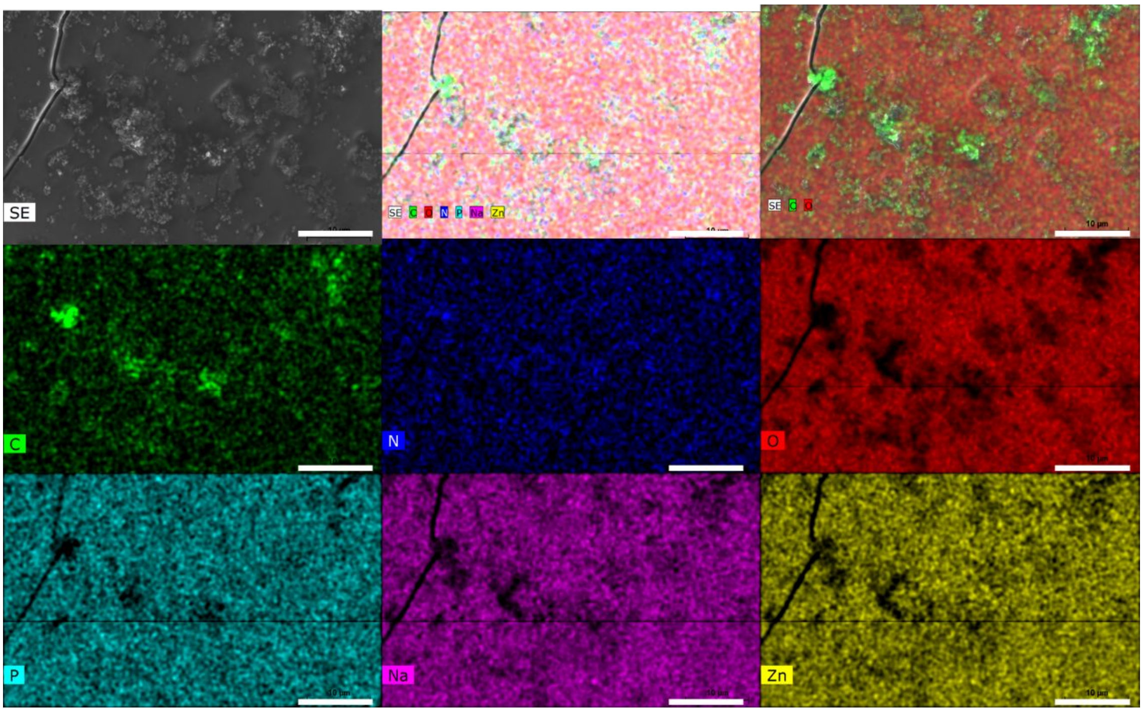

Figure S39. Mapping of inside of the composite pellet [$(ZIF\text{-}8)_{0.1}(IG)_{0.9}$]. First raw left to right: Image of the analysed area, all individual elements mappings, carbon and oxygen map. Carbon is related with the amount of ZIF-8 and oxygen with the amount of glass. Second and third raw individual map per element: C (green), O (red), N (blue), P (cyan), Na (pink), Zn (yellow). Scale 7 µm (white bar).

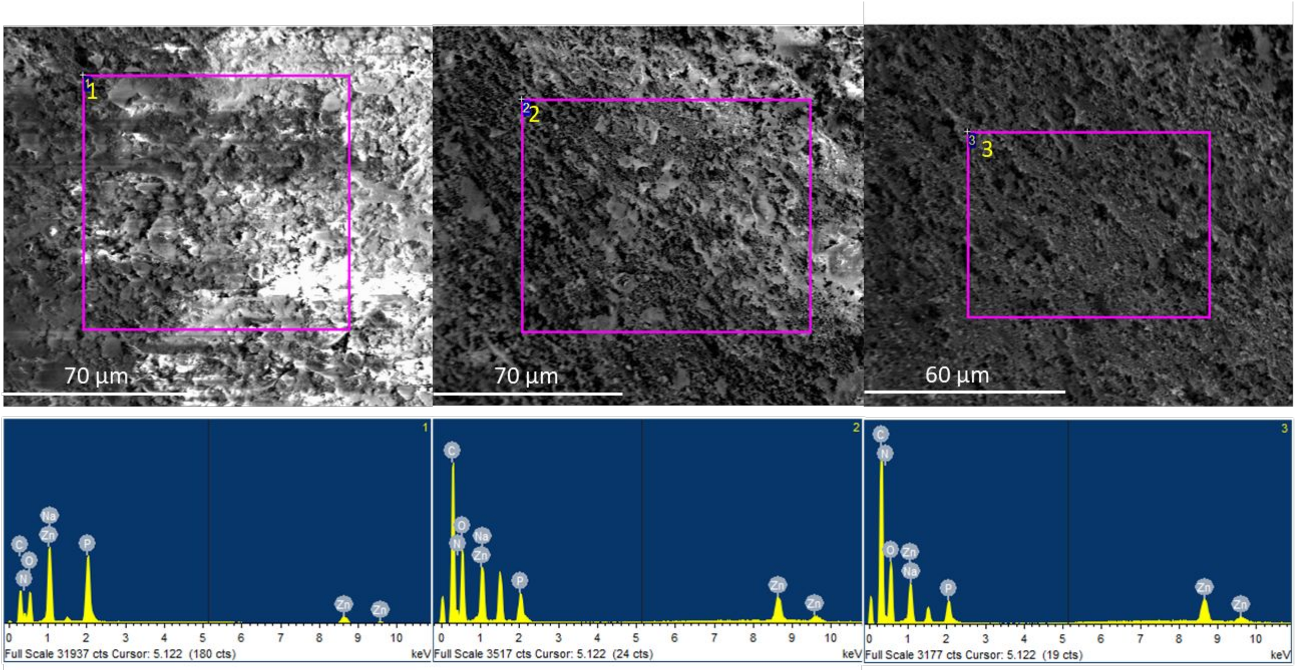

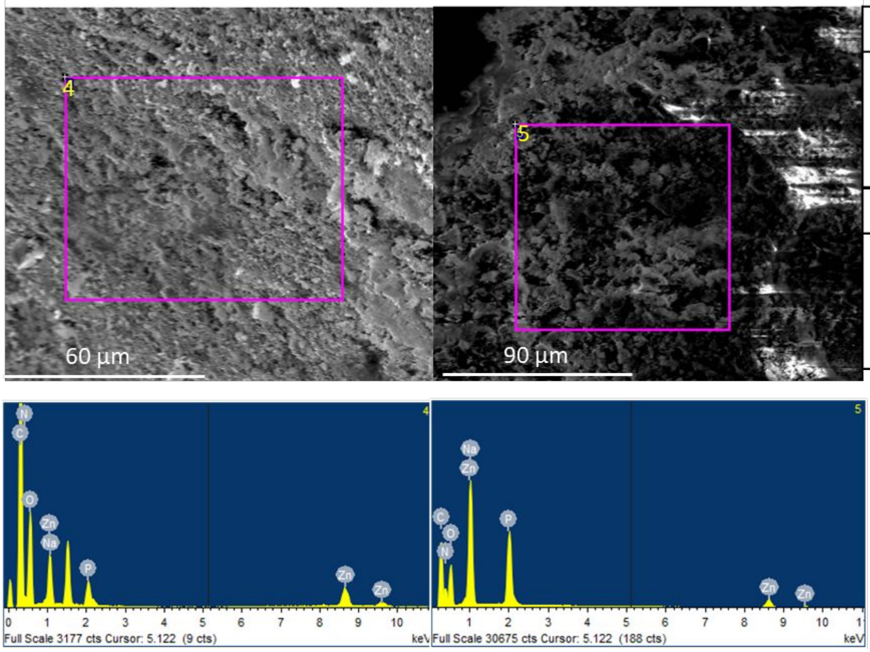

Atom	Atomic %	Error
P	5.58	1.84
Na	5.10	2.32
Zn	7.02	3.08
**Atom**	**Weight %**	**Error**
P	7.02	2.41
Na	8.62	3.77
Zn	24.49	9.79

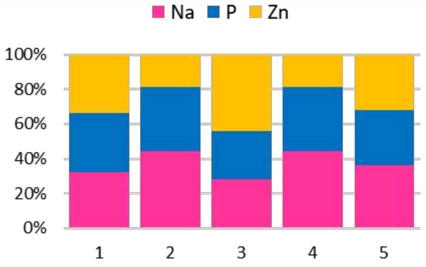

Figure S40. EDS-analyses of [$(ZIF\text{-}8)_{0.3}(IG)_{0.7}$] composite. Five different areas of the material depicted in pink were analysed with their corresponding spectrum. %Atomic and Weight of Na, P and Zn results were summarised in tables and graph (bottom right).

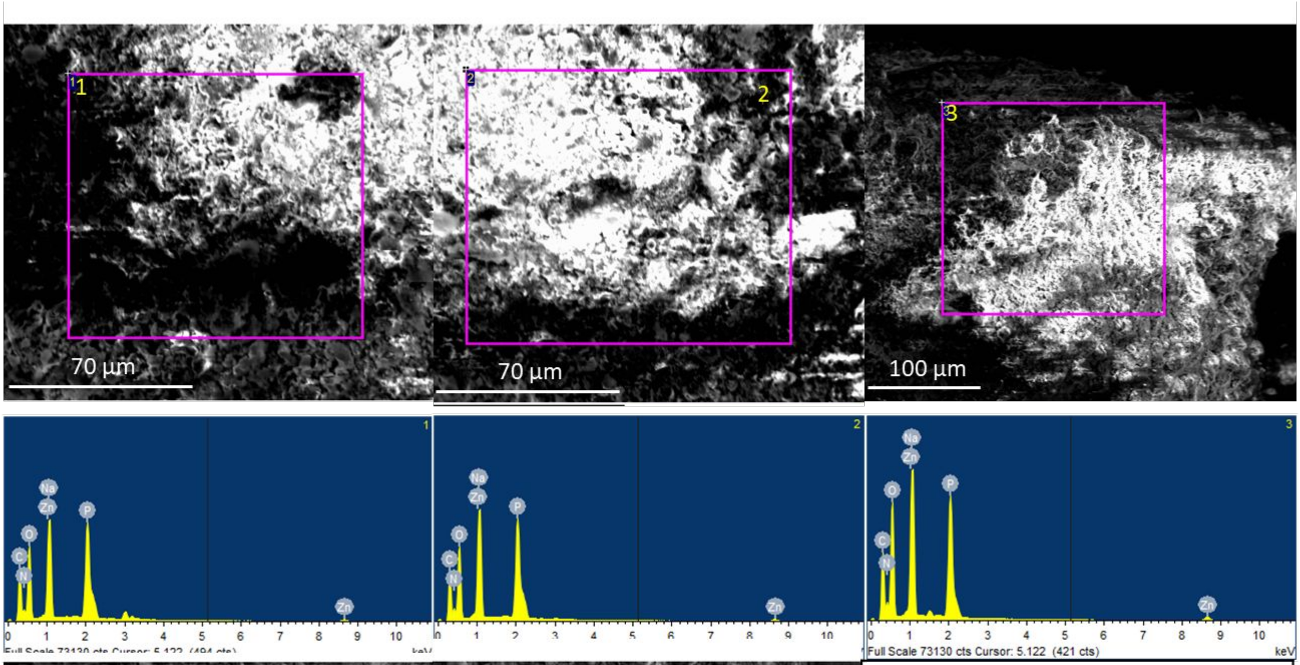

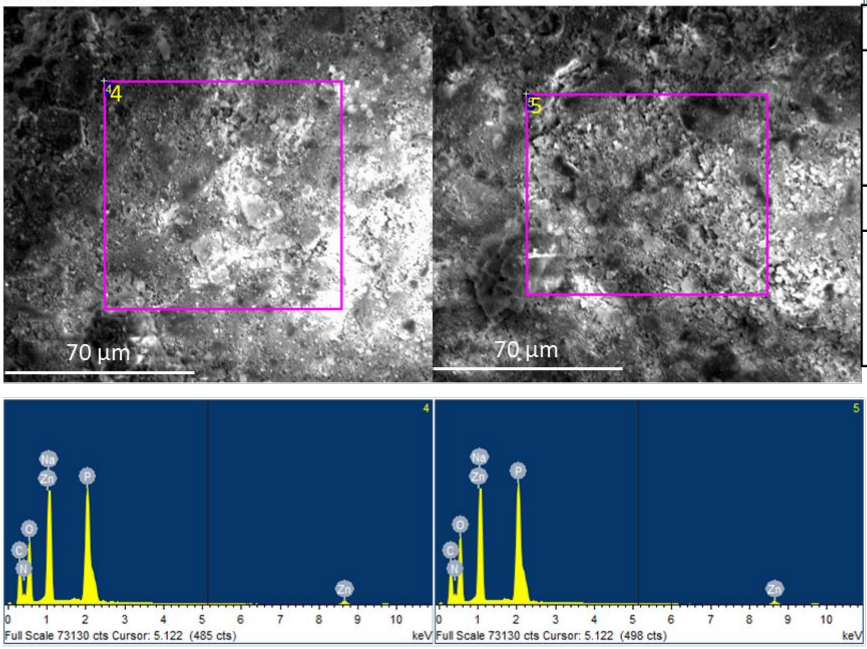

Atom	Atomic %	Error
P	11.30	0.72
Na	13.95	1.79
Zn	7.74	2.12
**Atom**	**Weight %**	**Error**
P	8.36	0.81
Na	7.67	1.24
Zn	2.03	0.63

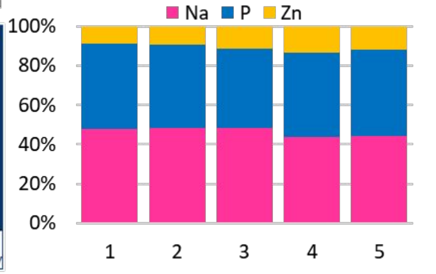

Figure S41. EDS-analyses of $[(ZIF\text{-}8)_{0.2}(IG)_{0.8}]$ composite. Five different areas of the material depicted in pink were analysed with their corresponding spectrum. %Atomic and Weight of Na, P and Zn results were summarised in tables and graph (bottom right).

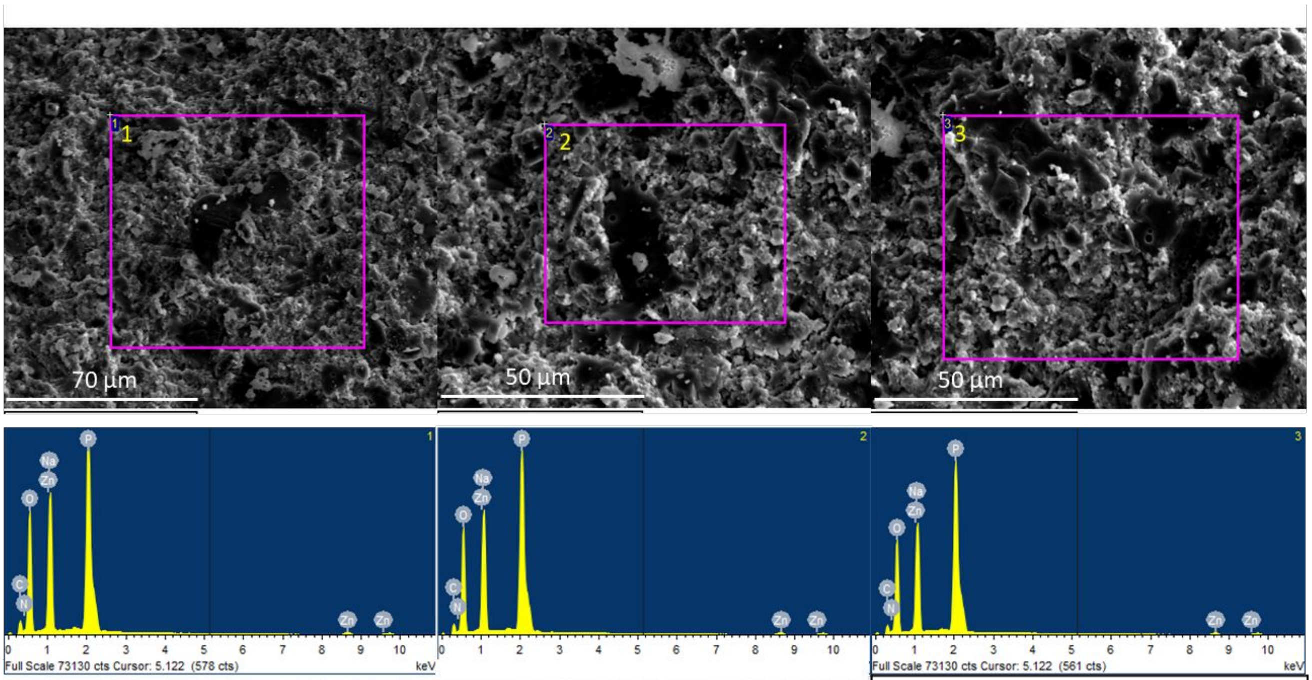

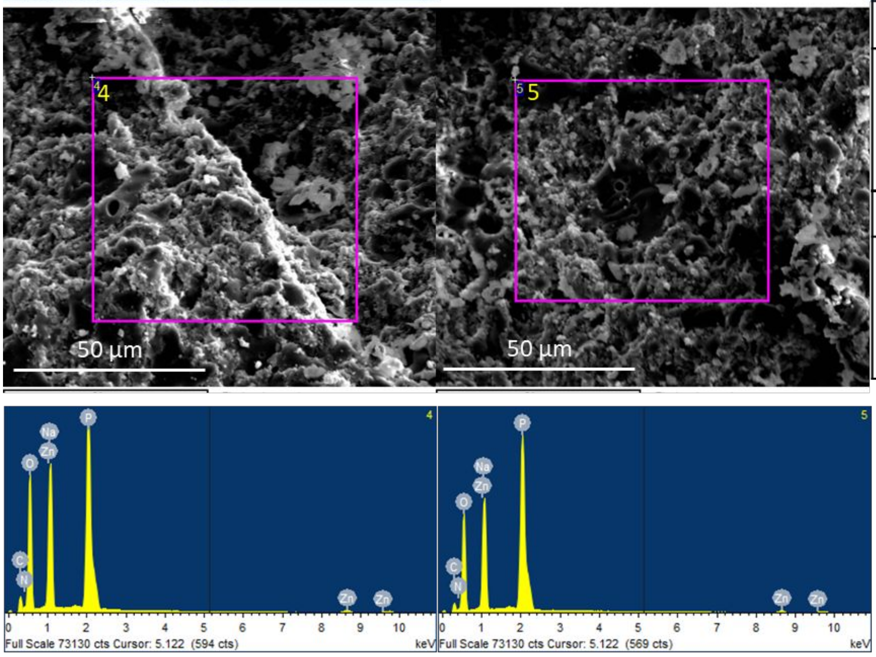

Atom	Atomic %	Error
P	14.15	0.06
Na	14.24	0.16
Zn	2.11	0.07

Atom	Weight %	Error
P	16.29	0.08
Na	22.10	0.24
Zn	6.91	0.22

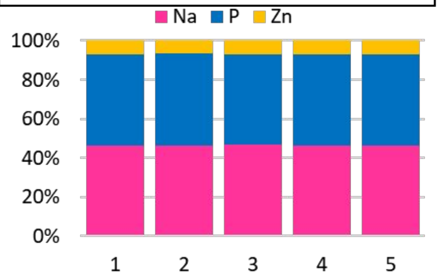

Figure S42. EDS-analyses of [$(ZIF\text{-}8)_{0.1}(IG)_{0.9}$] composite. Five different areas of the material depicted in pink were analysed with their corresponding spectrum. %Atomic and Weight of Na, P and Zn results were summarised in tables and graph (bottom right).

8. Pair distribution function study

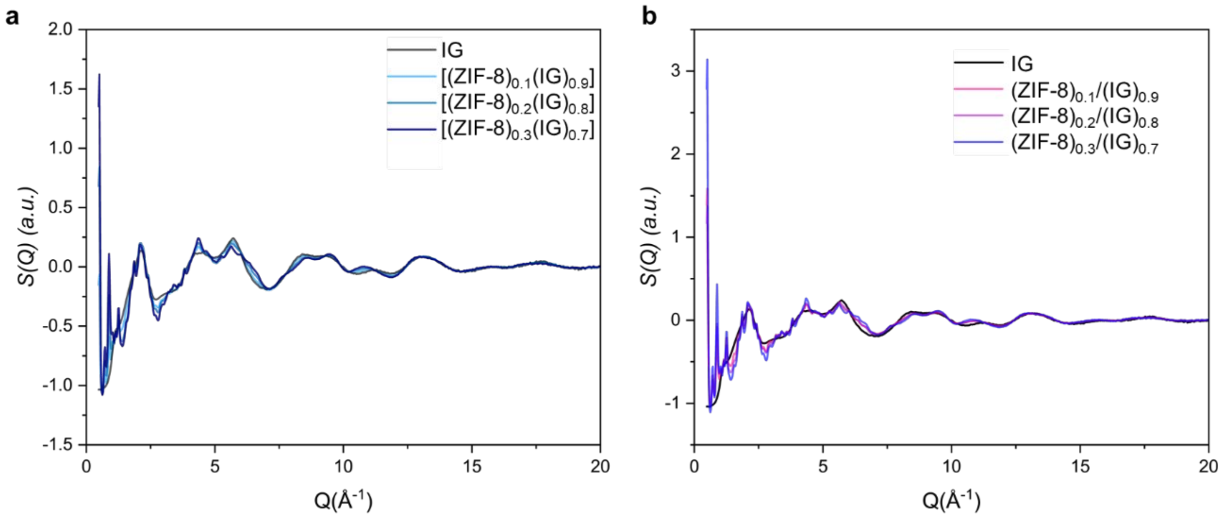

Figure S43. a. Structure factor *S(Q)* of 50(Na_2O)-50(P_2O_5) glass and the composites; **b**. Structure factor *S(Q)* of 50(Na_2O)50(P_2O_5) glass and the physical mixtures.

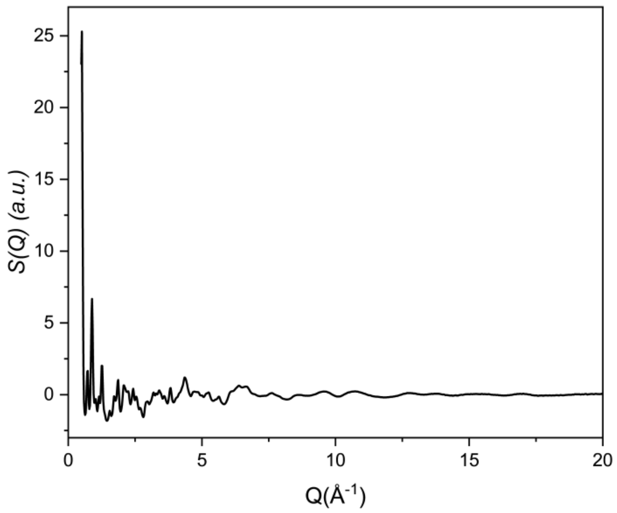

Figure S44. Structure factor *S(Q)* of ZIF-8 pristine.

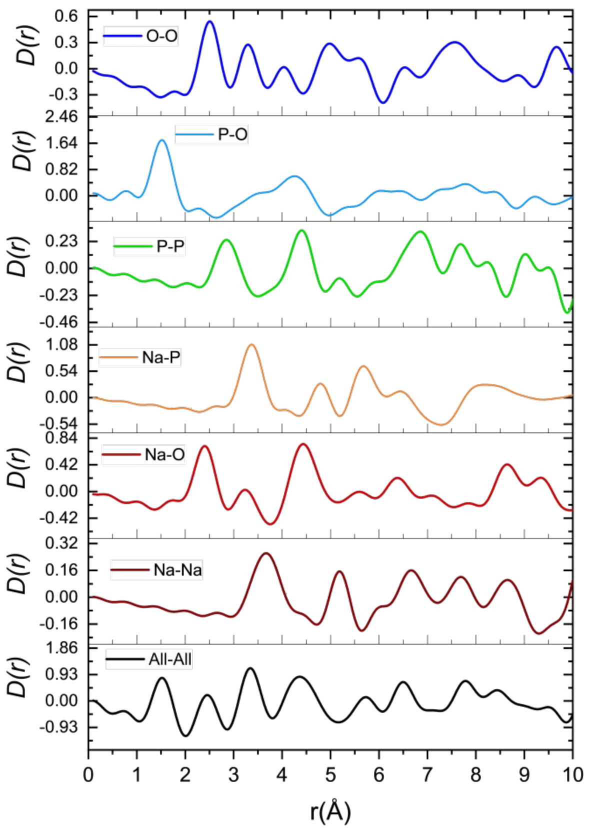

Figure S45. Calculated total and partial X-ray pair distribution function *g(r)* of the phase obtained after the recrystallisation process with PDFGUI.[5]

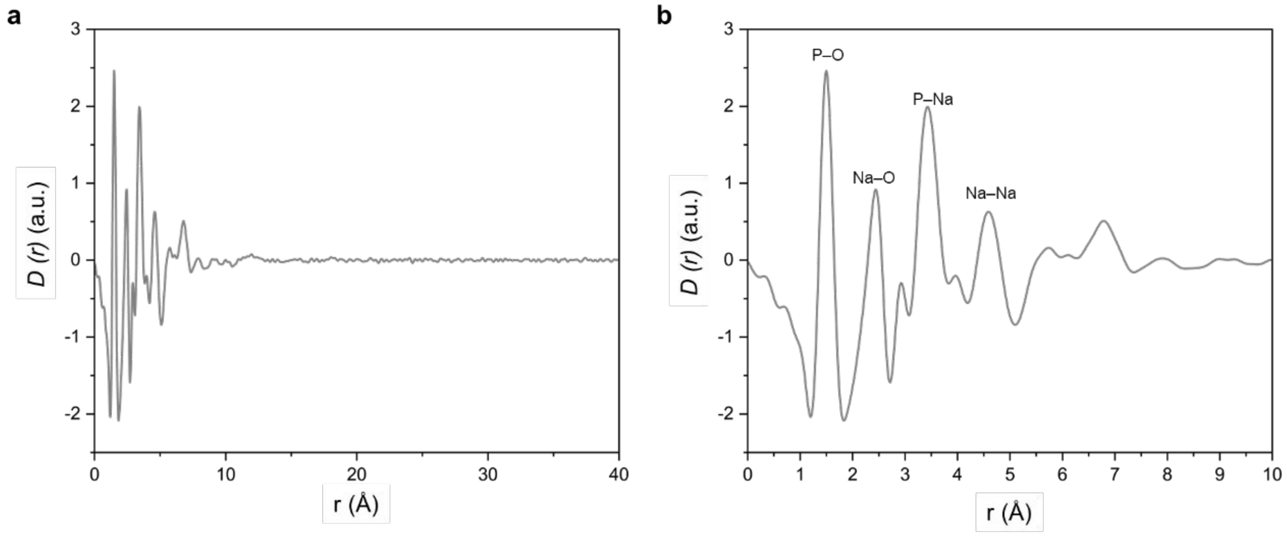

Figure S46. a. X-ray pair distribution function *D(r)* of the 50(Na_2O)50(P_2O_5) glass (IG). **b**. *D(r)* plotted from 1-10 Å to show short-range order and principal contributions.

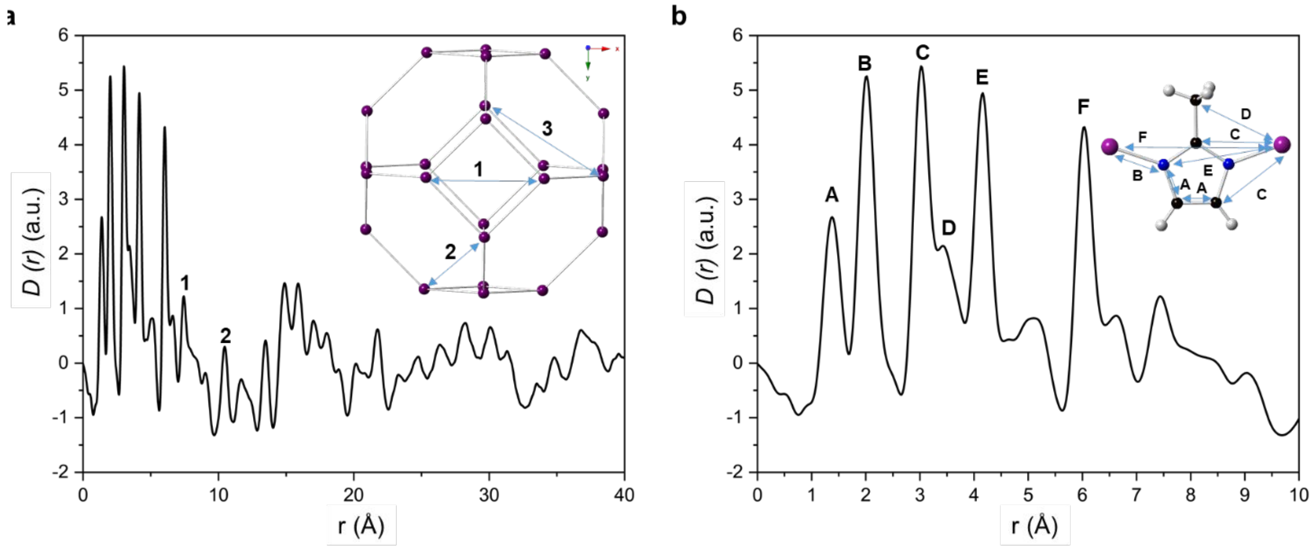

Figure S47. a. X-ray pair distribution function *D(r)* of ZIF-8. **b**. *D(r)* plotted from 1-10 Å to show short-range order and principal contributions. Zinc (purple), nitrogen (blue), carbon (black), hydrogen (white).

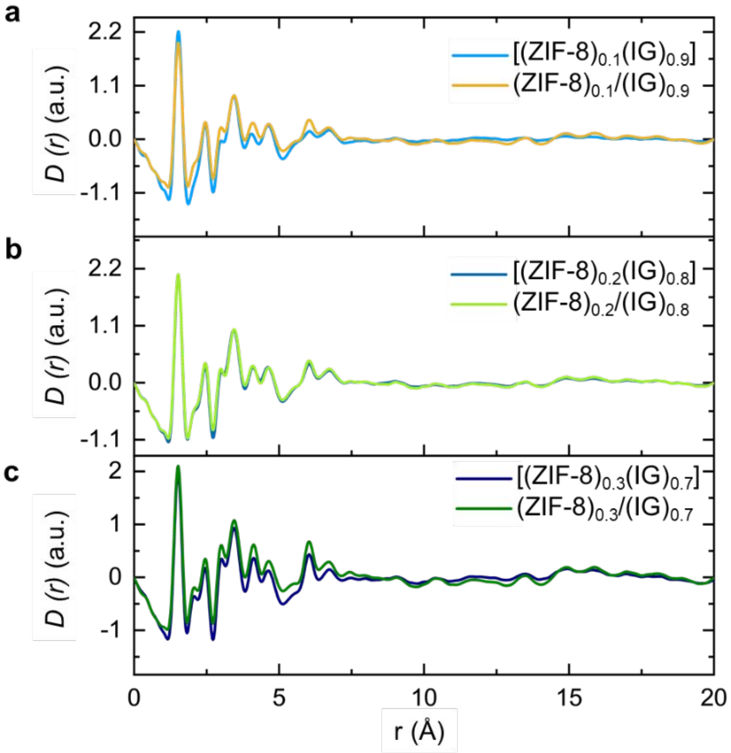

Figure S48. a. X-ray pair distribution function *D(r)* of [$(ZIF\text{-}8)_{0.1}(IG)_{0.9}$] MOF-CIGC (cyan) and its physical mixture (orange); **b**. *D(r)* of [$(ZIF\text{-}8)_{0.2}(IG)_{0.8}$] MOF-CIGC (turquoise) and its physical mixture (light green). **c**. *D(r)* of [$(ZIF\text{-}8)_{0.2}(IG)_{0.8}$] MOF-CIGC (navy) and its physical mixture (dark green). Slight differences appear between physical mixtures and their correspondent composites.

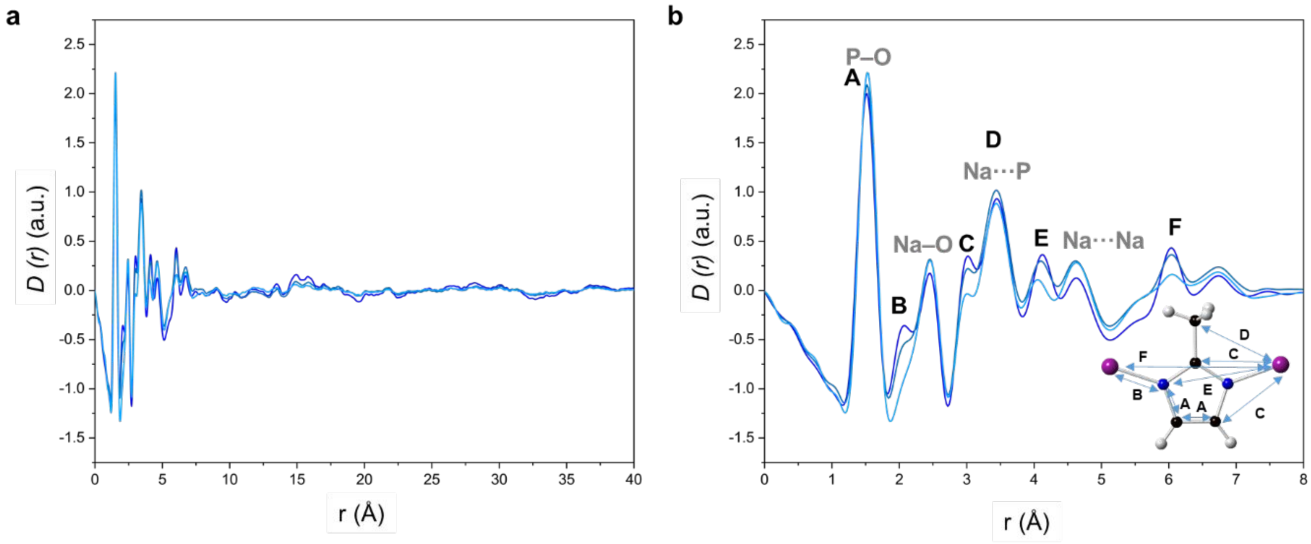

Figure S49. a. X-ray pair distribution function *D(r)* of composites. **b**. *D(r)* plotted from 1-10 Å to show short-range order and principal contributions. $[(ZIF\text{-}8)_{0.3}(IG)_{0.7}]$ (navy), $[(ZIF\text{-}8)_{0.2}(IG)_{0.8}]$ (turquoise) and $[(ZIF\text{-}8)_{0.1}(IG)_{0.9}]$ (light blue).

9. FTIR

ATR-FTIR spectrum of ZIF-8 (**Fig. S49.A**) exhibits typical bands explained in the literature.[6,7] The band at 2956 cm^{-1} is attributed to the C-H stretching mode of the methyl group in the imidazole. The stretching modes of the C-N-C present adsorption bands at 1363 and 1089 cm^{-1}. Typical band for C=N stretch is located at 1580. Bands at 1145, 990 and 760 cm^{-1}, respectively are related with C-N stretching.

Inorganic glass spectrum shows the evidence of water in a broad band at 3500 cm^{-1}. Peaks stretching modes were assigned according the literature.[8] An intense band at 1257 is related with asymmetric (PO_2) stretch. Symmetric stretch of (PO_2) appears as a shoulder at 1154 cm^{-1}. Band located at 1085 cm^{-1} is assigned as (PO_3^{-2}) asymmetric stretching. Intense band at 861 cm^{-1} is allocated as (P-O-P) asymmetric stretch. Two broad bands at 767 and 712 cm^{-1} were assigned as symmetric (P-O-P) stretch.

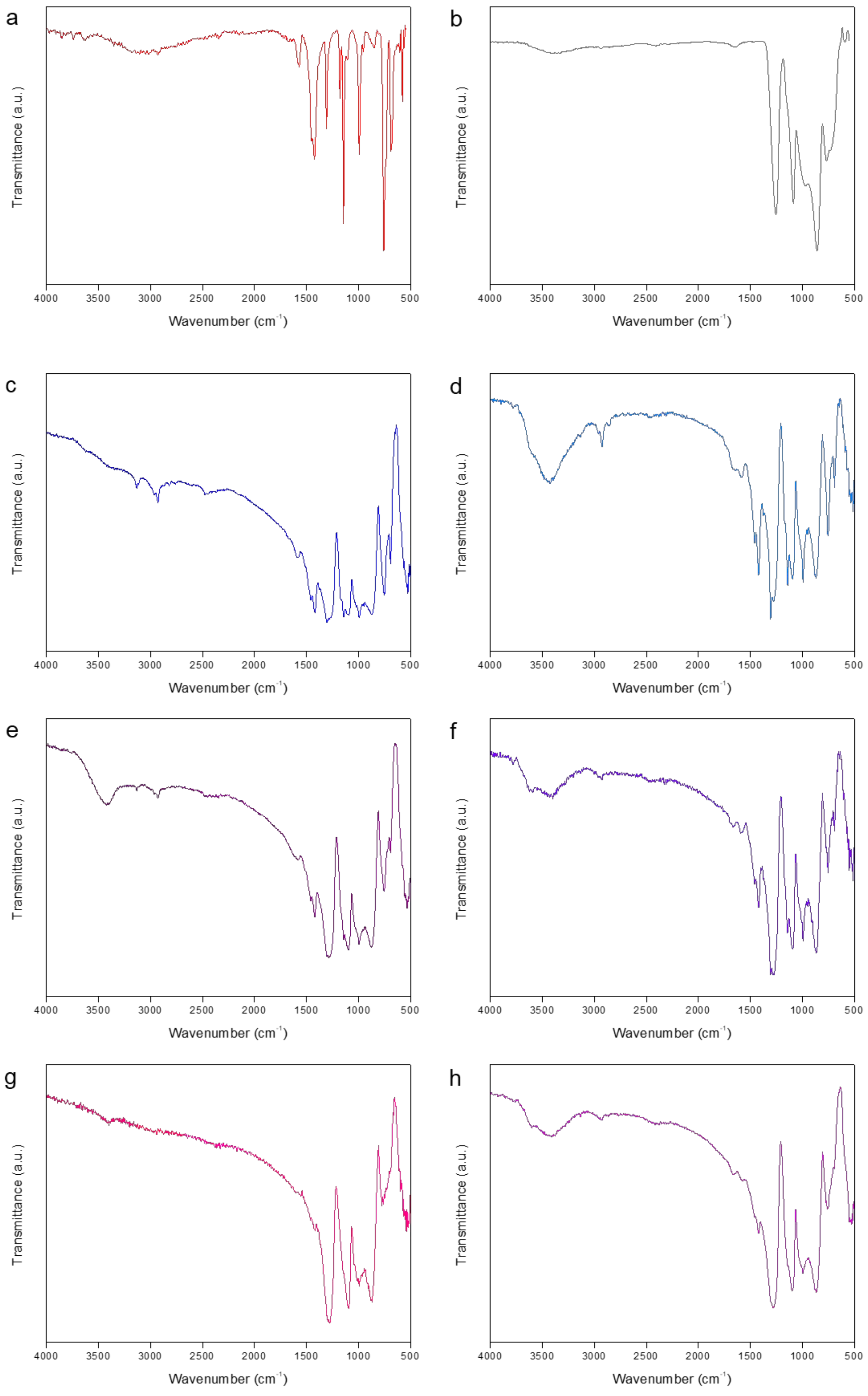

Figure S50. FTIR spectra for **a**. ZIF-8, **b**. inorganic glass (IG), **c**. $[(ZIF\text{-}8)_{0.3}(IG)_{0.7}]$ composite, **d.** $(ZIF\text{-}8)_{0.3}/(IG)_{0.7}$ physical mixture, **e**. $[(ZIF\text{-}8)_{0.2}(IG)_{0.8}]$ composite, **f**. $(ZIF\text{-}8)_{0.2}/(IG)_{0.8}$ physical mixture, **g**. $[(ZIF\text{-}8)_{0.1}(IG)_{0.9}]$ composite, **h**. $(ZIF\text{-}8)_{0.1}/(IG)_{0.9}$ physical mixture.

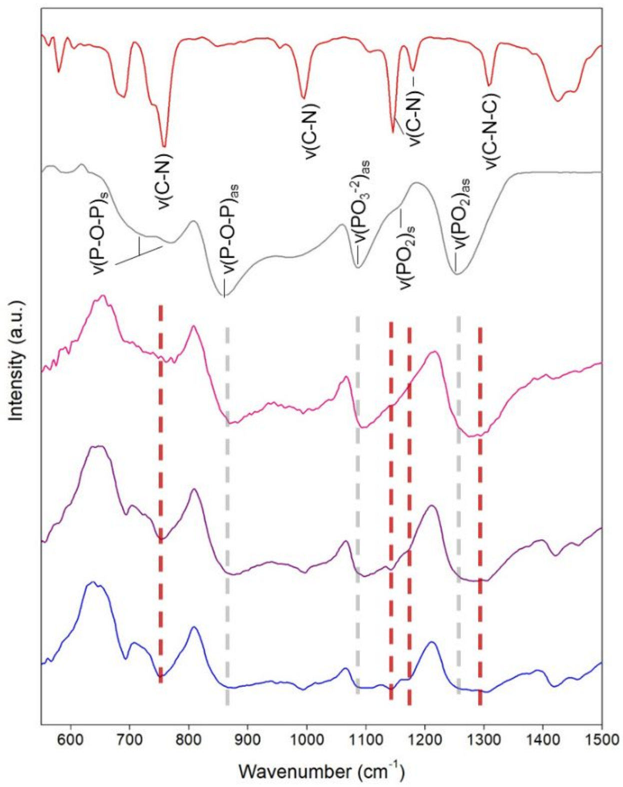

Figure S51. FTIR spectra comparison with the main stretching bands of the ZIF-8 (red), inorganic glass (grey), [(ZIF-8)$_{0.1}$(IG)$_{0.9}$] (pink), [(ZIF-8)$_{0.2}$(IG)$_{0.8}$] purple) and [(ZIF-8)$_{0.3}$(IG)$_{0.7}$] (blue).

10. Raman Spectroscopy

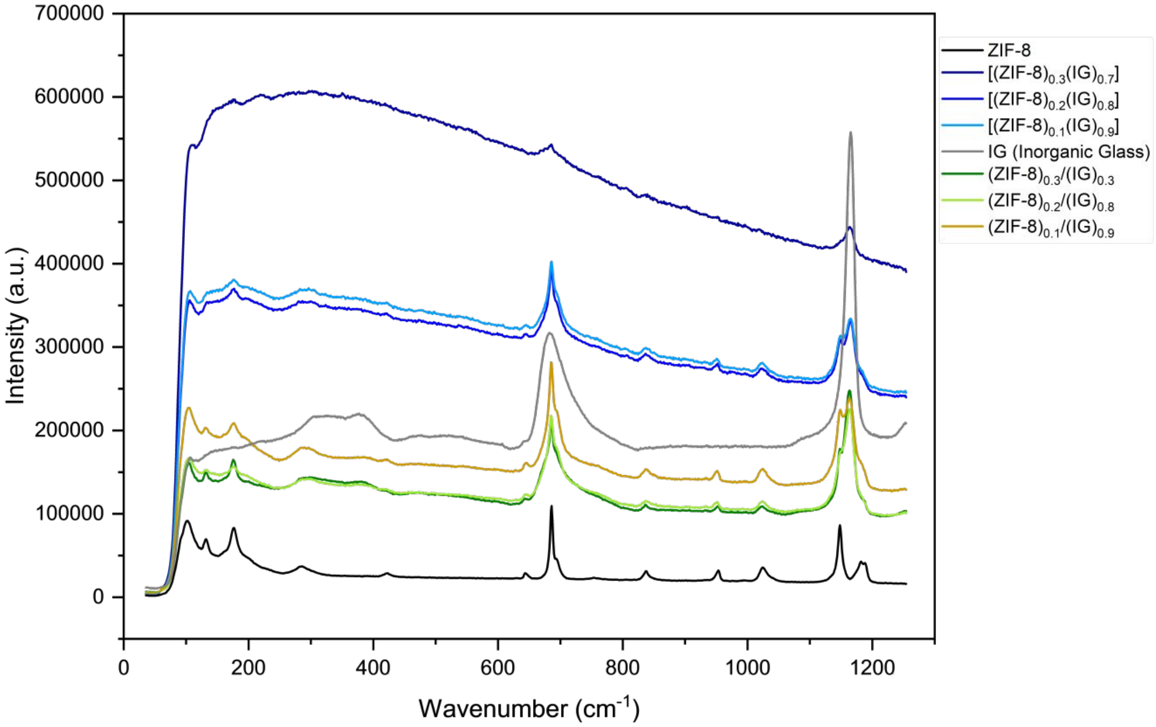

Figure S52. Raman spectrums for the inorganic glass (IG) (grey) ZIF-8 (black), [(ZIF-8)$_{0.3}$(IG)$_{0.7}$] composite (navy), [(ZIF-8)$_{0.2}$(IG)$_{0.8}$] composite (blue), [(ZIF-8)$_{0.1}$(IG)$_{0.9}$] composite (cyan), (ZIF-8)$_{0.3}$/(IG)$_{0.7}$ physical mixture (dark green), (ZIF-8)$_{0.2}$/(IG)$_{0.8}$ physical mixture (light green) and (ZIF-8)$_{0.1}$/(IG)$_{0.9}$ physical mixture (yellow). All composites showed a pronounced background signal and does not allow to identify potential new bands.

11. Nuclear Magnetic Resonance

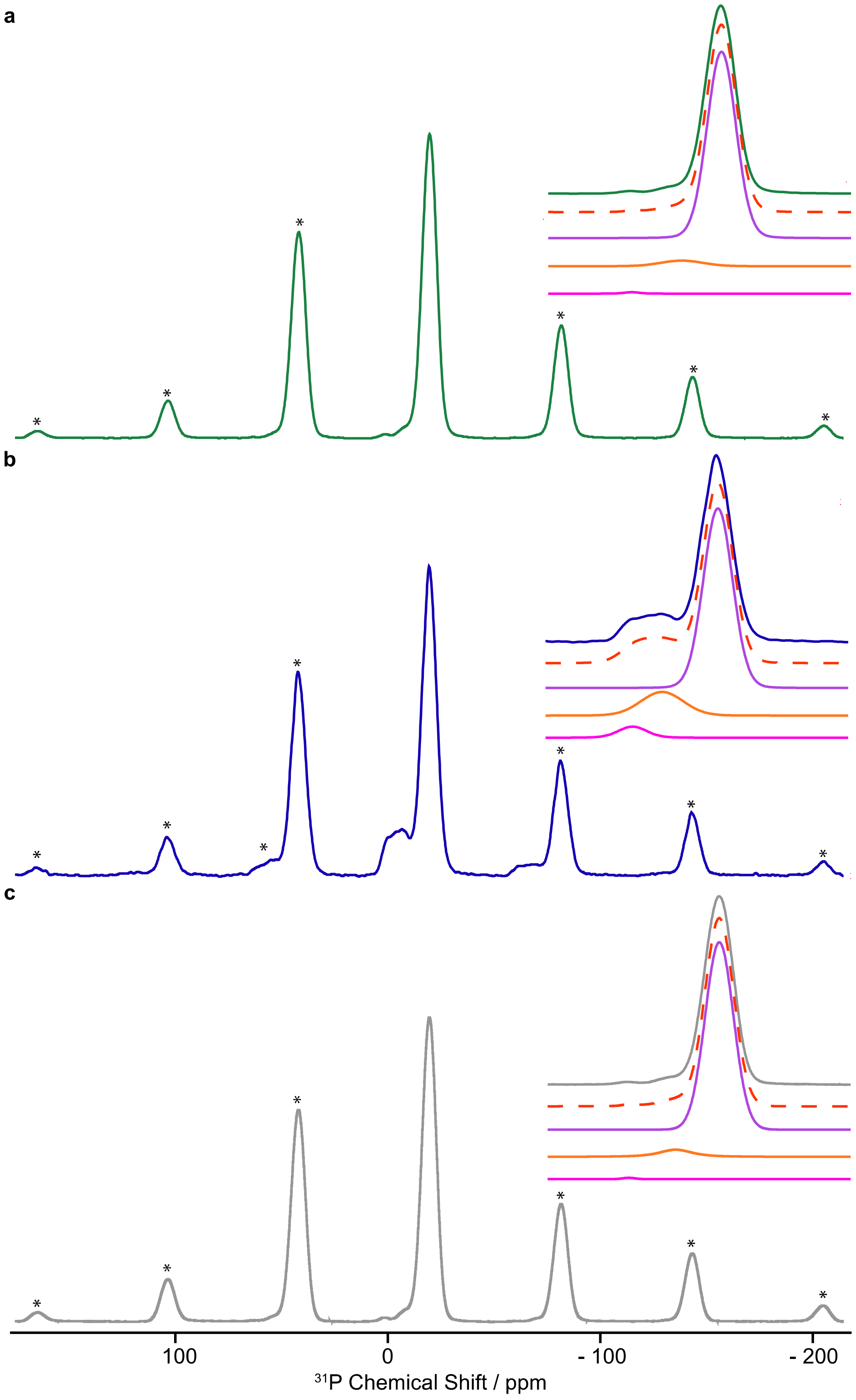

Figure S53. Stacked plot of the ^{31}P directly excited MAS NMR spectra for **a**. $(ZIF\text{-}8)_{0.3}/(IG)_{0.7}$ physical mixture, **b**. $[(ZIF\text{-}8)_{0.3}(IG)_{0.7}]$ composite and **c**. $50(Na_2O)\text{-}50(P_2O_5)$ inorganic glass. Data were recorded at 9.4 T, under a MAS frequency of 10 kHz and using quantitative recycle delays of 30 s. A magnified view of the isotropic region in the 10 to -35 ppm range is shown as inserts which also contain simulated spectra (dashed red lines) decomposed into the different Q^2 (purple), Q^1 (orange) and Q^0 (pink) phosphorous environments. Asterisks (*) denote spinning sidebands.

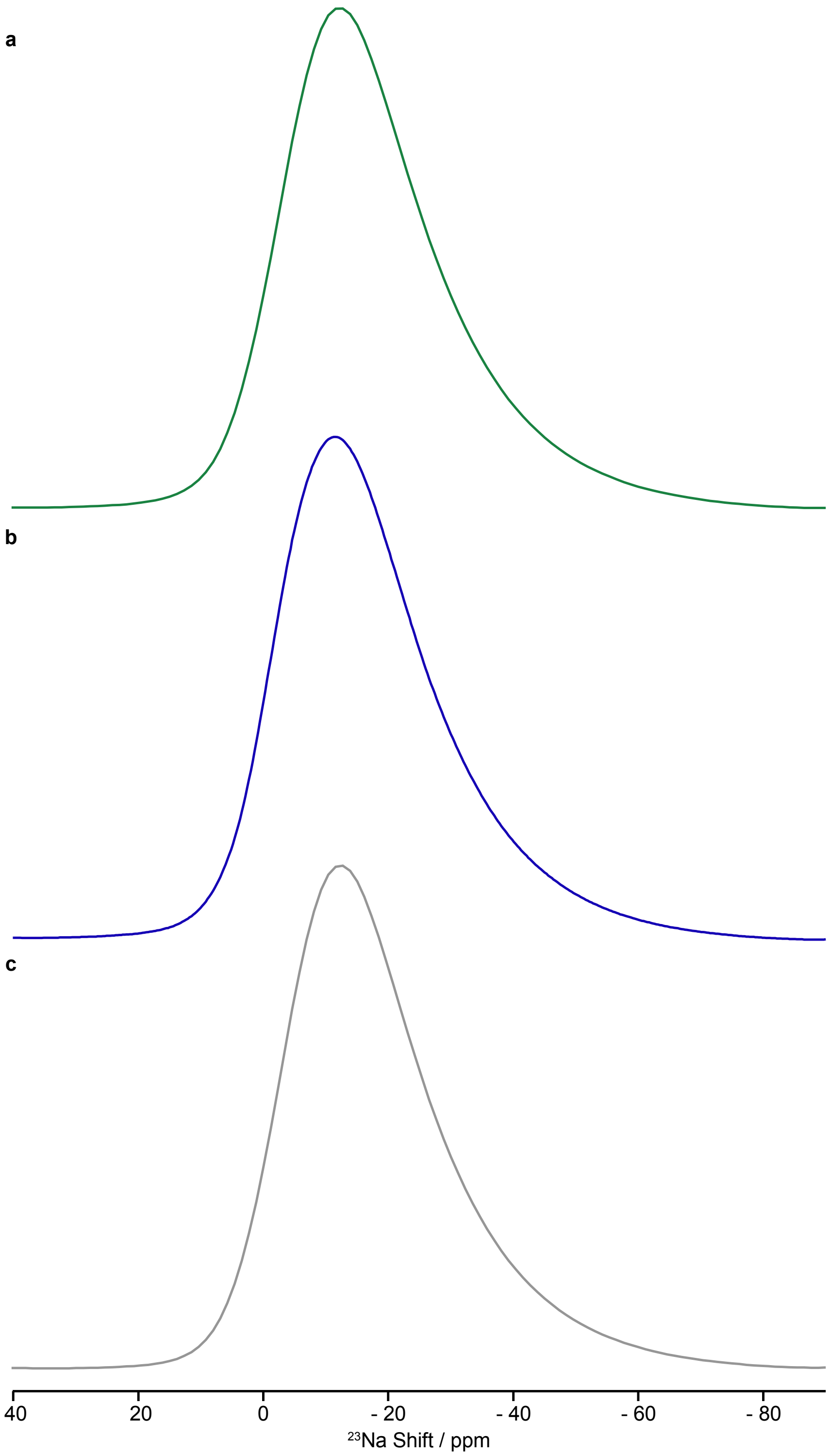

Figure S54. Stacked plot of the ^{23}Na directly excited MAS NMR spectra for **a**. $(ZIF\text{-}8)_{0.3}/(IG)_{0.7}$ physical mixture, **b**. $[(ZIF\text{-}8)_{0.3}(IG)_{0.7}]$ composite and **c**. 50(Na_2O)-50(P_2O_5) inorganic glass. Only the isotropic resonance is shown. Data were recorded at 9.4 T under a MAS frequency of 10 kHz and using quantitative recycle delay of 5 s.

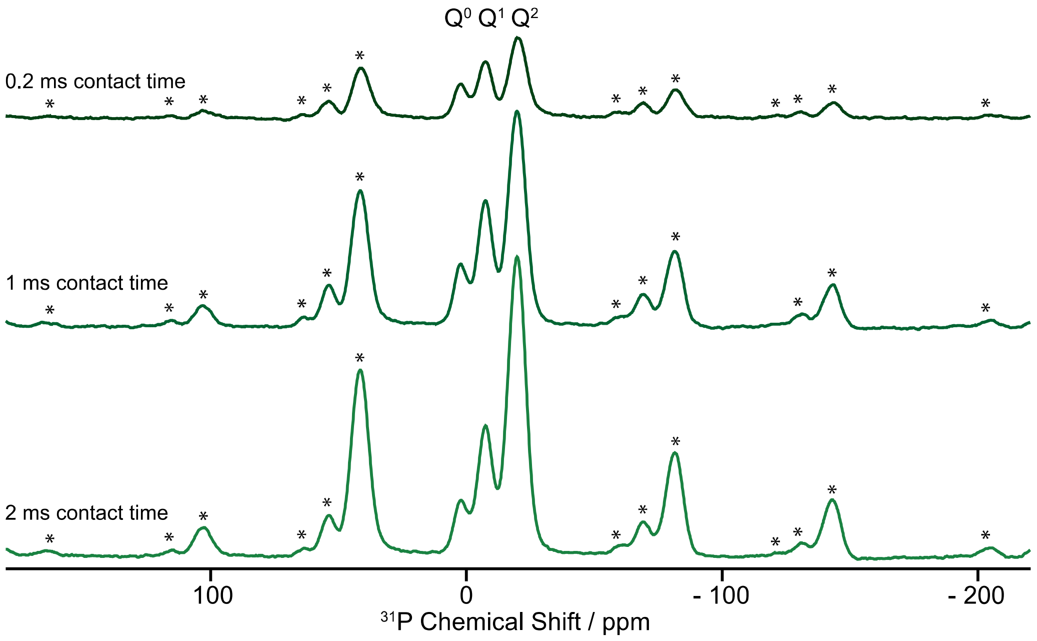

Figure S55. ^{31}P CP MAS NMR spectra of $(ZIF\text{-}8)_{0.3}/(IG)_{0.7}$ physical mixture as a function of the CP contact times. Data were recorded at 9.4 T under a MAS frequency of 10 kHz and using recycle delays of 6 s (approx.1.3 x T_1). Asterisks (*) denote spinning sidebands.

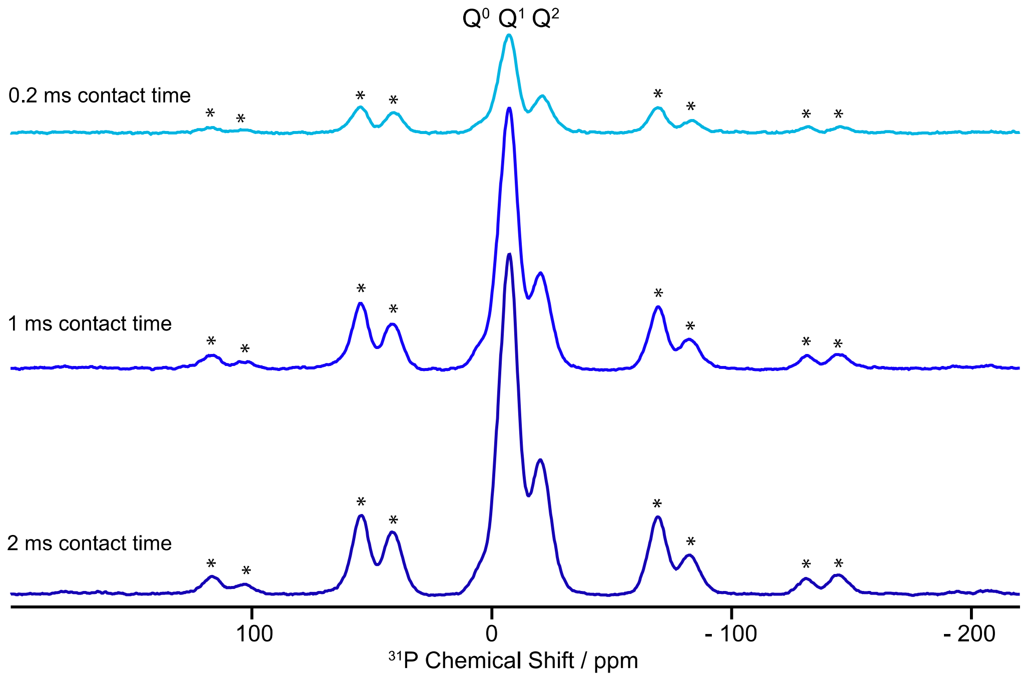

Figure S56. ^{31}P CP MAS NMR spectra of $[(ZIF\text{-}8)_{0.3}(IG)_{0.7}]$ composite as a function of the CP contact times. Data were recorded at 9.4 T under a MAS frequency of 10 kHz and using recycle delays of 4.5 s (approx. 1.3 x T_1). Asterisks (*) denote spinning sidebands.

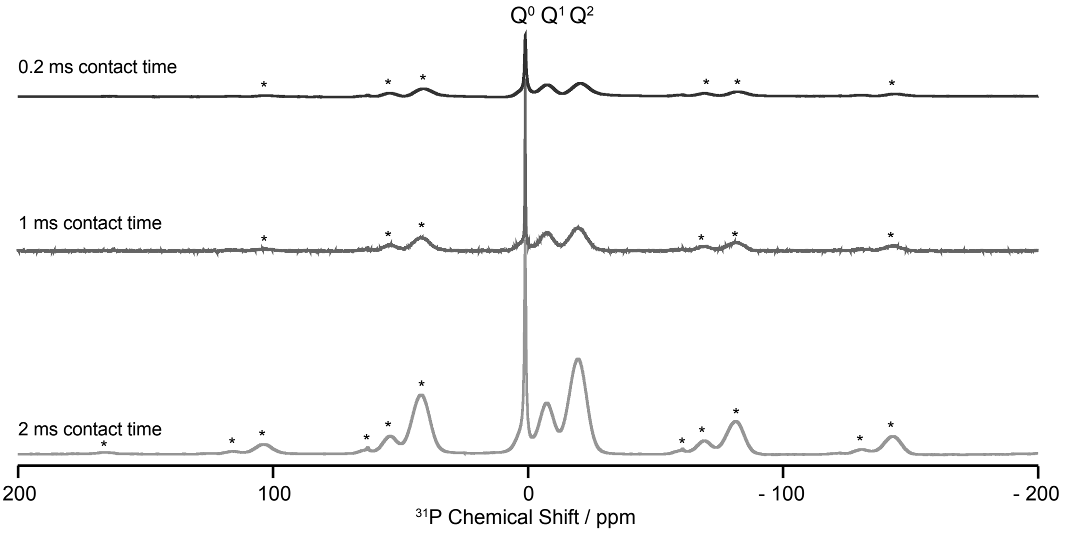

Figure S57. ^{31}P CP MAS NMR spectra of $50(Na_2O)\text{-}50(P_2O_5)$ inorganic glass as a function of the CP contact times. Data were recorded at 9.4 T under a MAS frequency of 10 kHz and using quantitative recycle delays of 4.5 s (> 5 x T_1). The spectrum at 1 ms contact time was recorded with fewer scans hence the lower signal to noise. Asterisks (*) denote spinning sidebands.

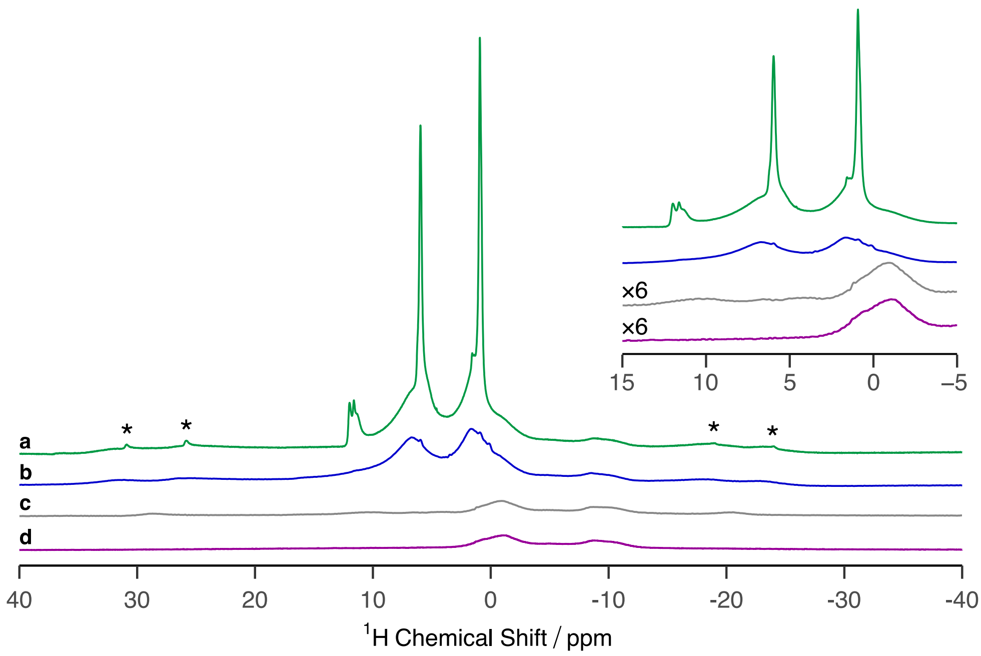

Figure S58. Stacked plot of the ^{1}H MAS NMR spectra for **a.** $(ZIF\text{-}8)_{0.3}/(IG)_{0.7}$ physical mixture (green), **b.** $[(ZIF\text{-}8)_{0.3}(IG)_{0.7}]$ composite (blue), **c.** $50(Na_2O)\text{-}50(P_2O_5)$ inorganic glass (grey) and **d.** NMR probe background using a rotor filled with a non-hydrogen containing sample (purple). Data were recorded at 9.4 T under a MAS frequency of 10 kHz and using quantitative recycle delays (5 x T_1) of 20 s for **a.**, **b.**, **d.** and 10 s for **c.** Asterisks (*) denote spinning sidebands. Magnified views of the spectral region between 15 ppm and -5 ppm (with ×6 intensity for the spectra of the $50(Na_2O)\text{-}50(P_2O_5)$ inorganic glass and NMR probe background) are shown above.

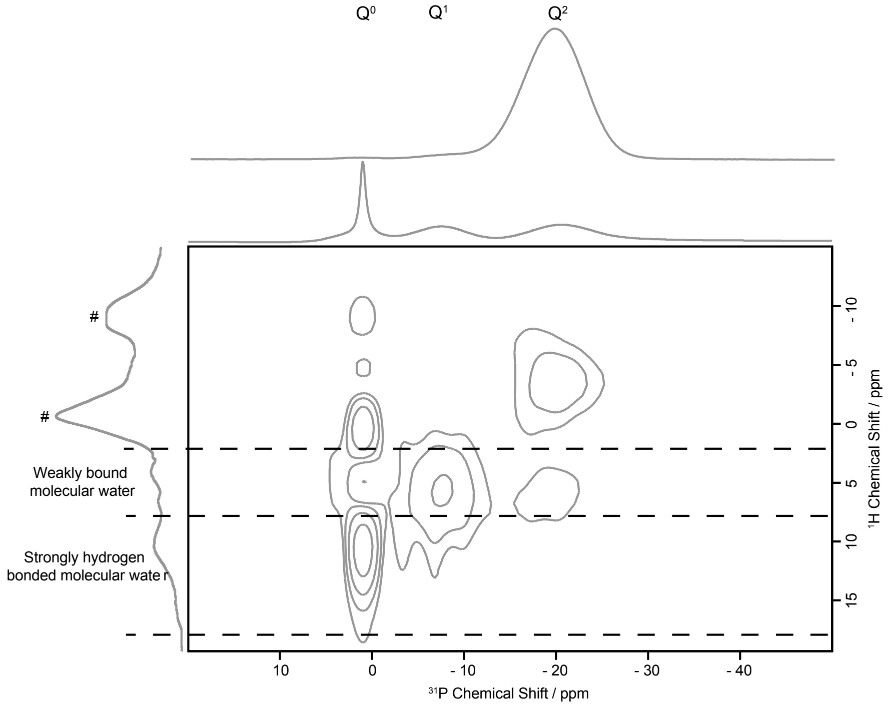

Figure S59. Two dimensional ^{1}H ^{31}P HETCOR NMR spectra of the 50(Na_2O)-50(P_2O_5) inorganic glass. Data were recorded at 9.4 T under a MAS frequency of 10 kHz and using quantitative recycle delays of 6 s (> 5 x T_1). A short contact time for CP of 0.2 ms was used. The vertical spectrum depicts the ^{1}H MAS spectra while the horizontal spectra display the ^{31}P MAS spectra with direct excitation (top) and CP (bottom), respectively). The spectral assignments given in the figure are tentative based on literature data for this glass.[9]

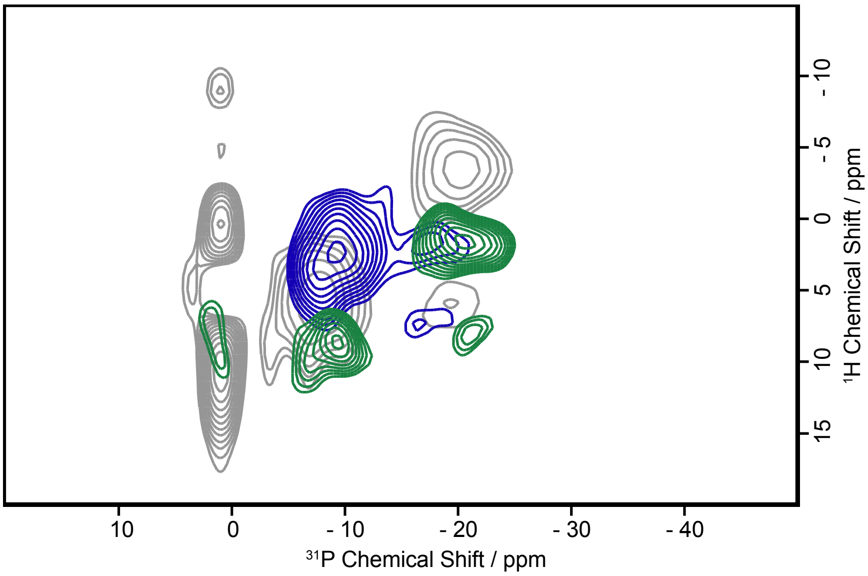

Figure S60. Comparison of two dimensional ^{1}H ^{31}P HETCOR NMR spectra of the ZIF-8)$_{0.3}$/(IG)$_{0.7}$ physical mixture (green), [(ZIF-8)$_{0.3}$(IG)$_{0.7}$] composite (blue) and 50(Na_2O)-50(P_2O_5) inorganic glass (grey). Data from Figures 6a, 6b and S59 are overlaid to facilitate the comparison.

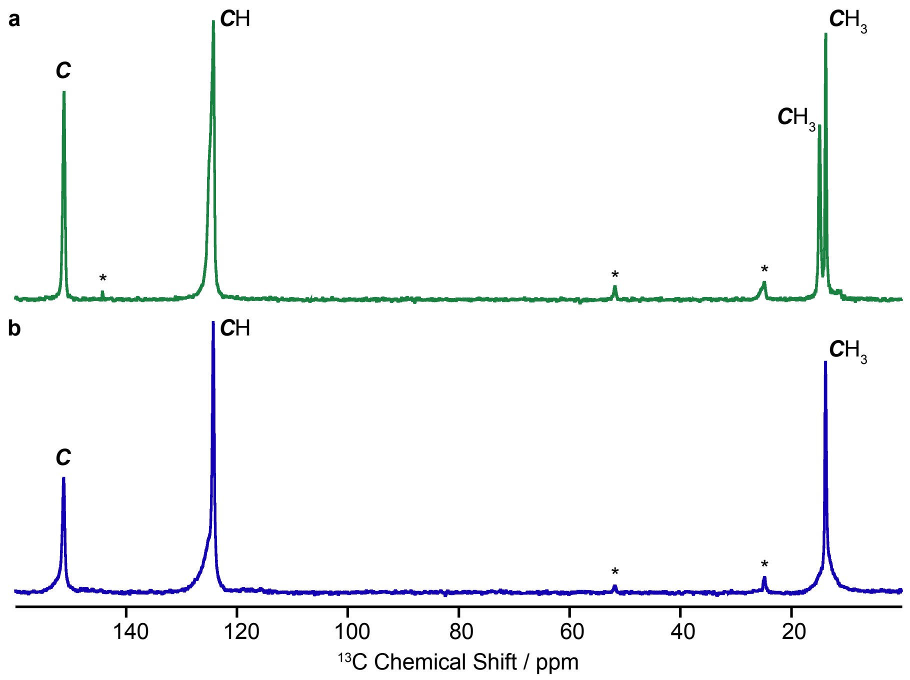

Figure S61. Stacked plot of the isotropic region of the ^{13}C CP MAS NMR spectra for **a**. $(ZIF\text{-}8)_{0.3}/(IG)_{0.7}$ physical mixture and **b**. $[(ZIF\text{-}8)_{0.3}(IG)_{0.7}]$ composite. Data were recorded at 9.4 T under a MAS frequency of 10 kHz and using a recycle delay of 5.5 s (approx. 1.3 x T_1). Asterisks (*) denote spinning sidebands.

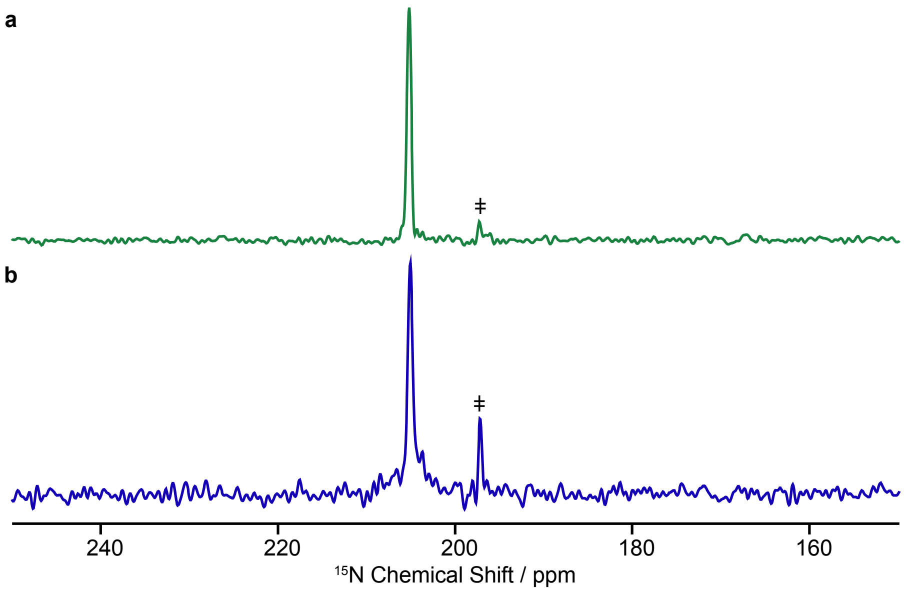

Figure S62. Stacked plot of the isotropic region of the ^{15}N CP MAS NMR spectra for **a**. $(ZIF\text{-}8)_{0.3}/(IG)_{0.7}$ physical mixture and **b**. $[(ZIF\text{-}8)_{0.3}(IG)_{0.7}]$ composite. Data were recorded at 9.4 T under a MAS frequency of 8 kHz and using a recycle delay of 5.5 s (approx. 1.3 x T_1). The symbols (‡) denote the position of the transmitted frequency.

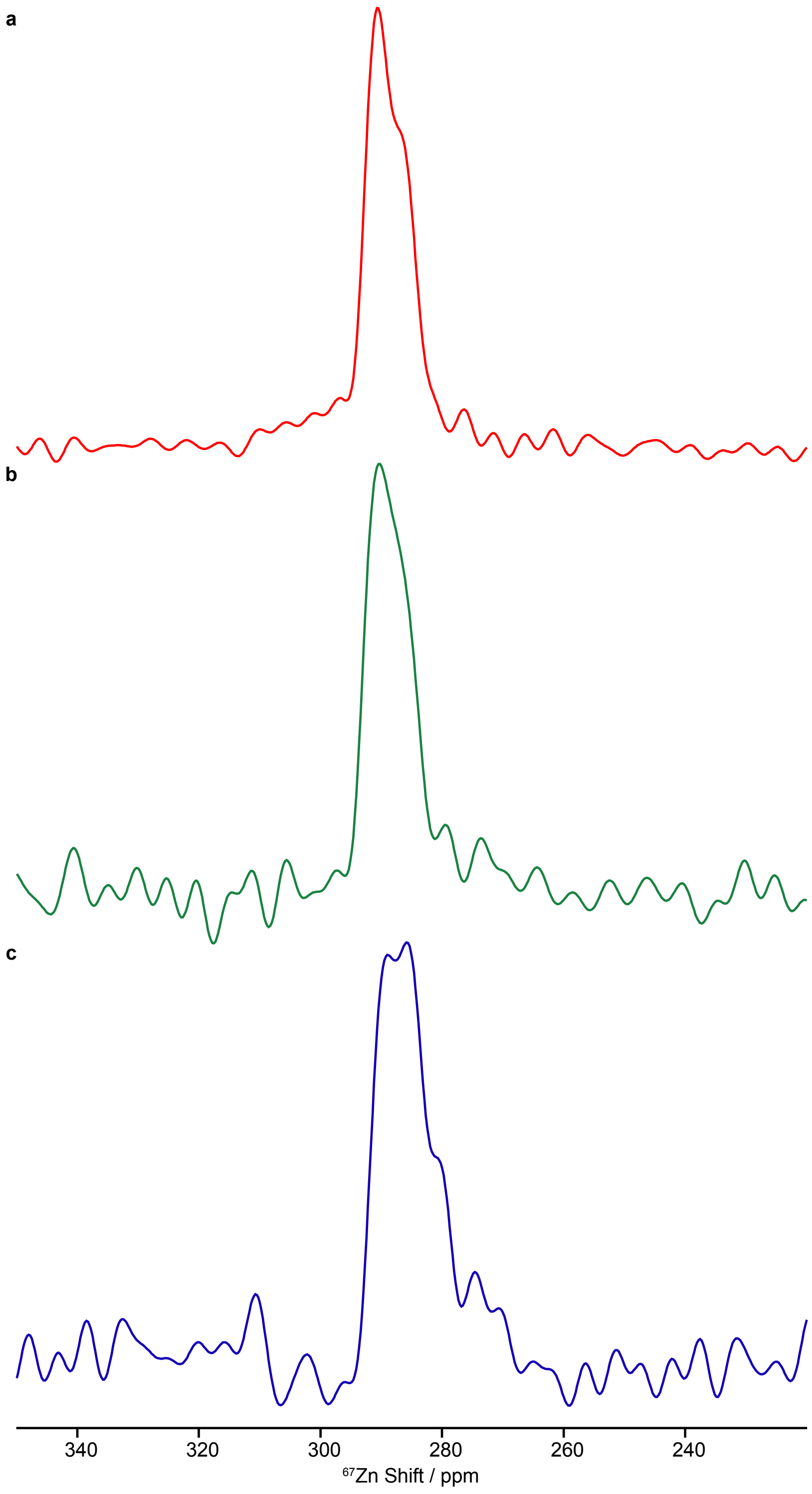

Figure S63. Comparison of the ^{67}Zn MAS NMR spectra for **a**. crystalline ZIF-8, **b**. $(ZIF\text{-}8)_{0.3}/(IG)_{0.7}$ physical mixture and **c**. $[(ZIF\text{-}8)_{0.3}(IG)_{0.7}]$ composite. Data were recorded at 18.8 T under a MAS frequency of 10 kHz. 261312, 409600 and 409600 scans were recorded for signal averaging using a recycle delay of 1 s. The broadening observed results from 2nd order quadrupolar broadening not averaged out by MAS.

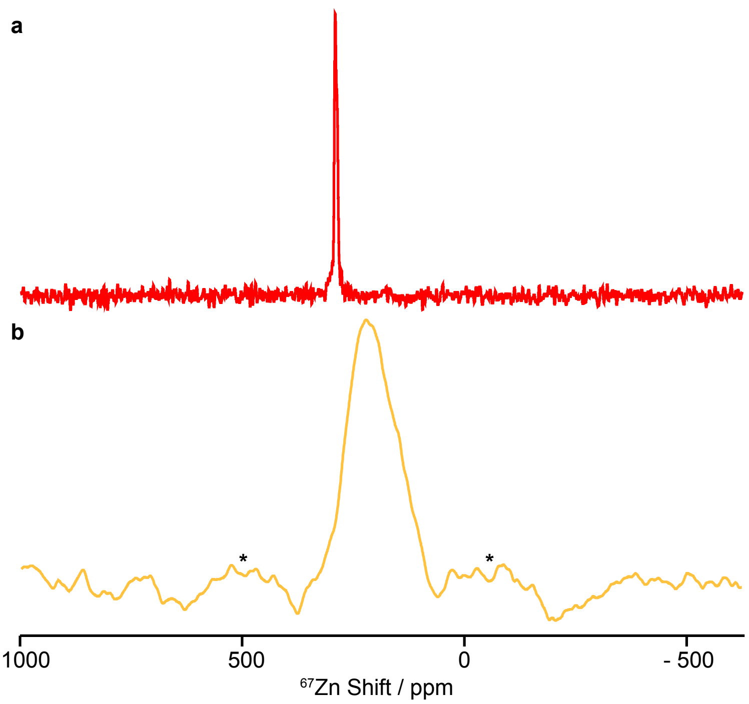

Figure S64. Comparison of the ^{67}Zn MAS NMR spectra for **a**. ZIF-8 crystalline and **b**. ZIF-8 mechanically amorphised ZIF-8. Data were recorded at 18.8 T under a MAS frequency of 10 kHz. 261312 and 841338 scans were recorded for signal averaging using recycle delays of 1 and 0.1 s in **a**. and **b**., respectively. The broadening observed in **b**. arises from a distribution of structural disorder. Asterisks (*) denote spinning sidebands.

11. Differential pair distribution function study

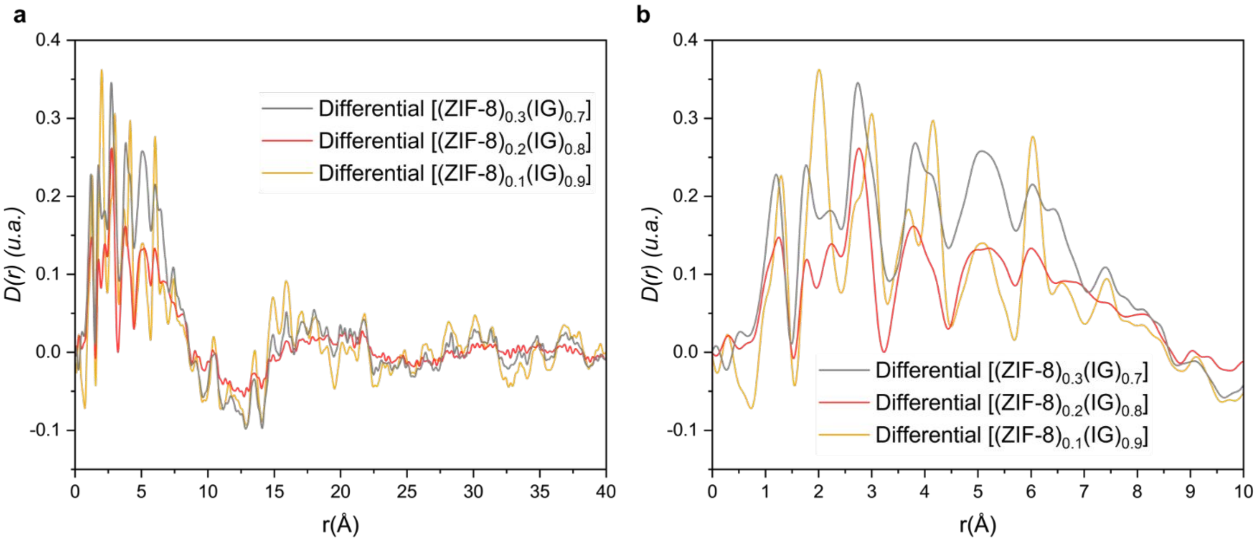

Figure S65. a. Differential PDFs obtained from subtracting the physical mixture from each corresponding composite. **b**. Zoom-in to show the local structure of the differential PDFs. Features at long-range order are similar, whilst the features in the local structure are significantly different, therefore this methodology is not a good approach.

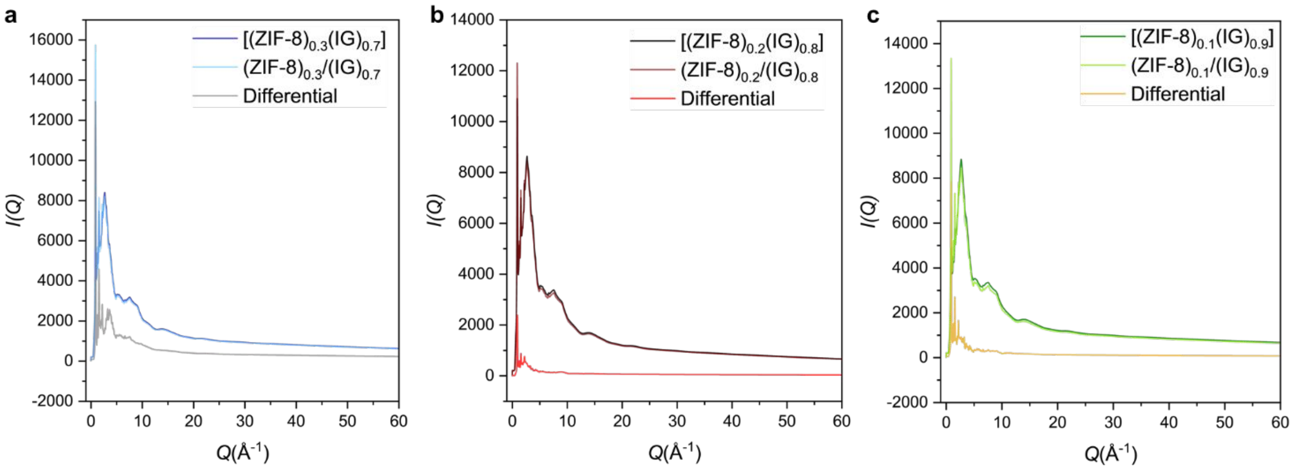

Figure S66. a. *I(Q)* data of [$(ZIF\text{-}8)_{0.3}(IG)_{0.7}$] composite, its corresponding physical mixture and the resulting differential from the subtracting the physical mixture from the composite. **b**. *I(Q)* data of [$(ZIF\text{-}8)_{0.2}(IG)_{0.8}$] composite, its corresponding physical mixture and the resulting differential from subtracting the physical mixture from the composite. **c**. *I(Q)* data of [$(ZIF\text{-}8)_{0.1}(IG)_{0.9}$] composite, its corresponding physical mixture and the resulting differential from the subtracting the physical mixture from the composite.

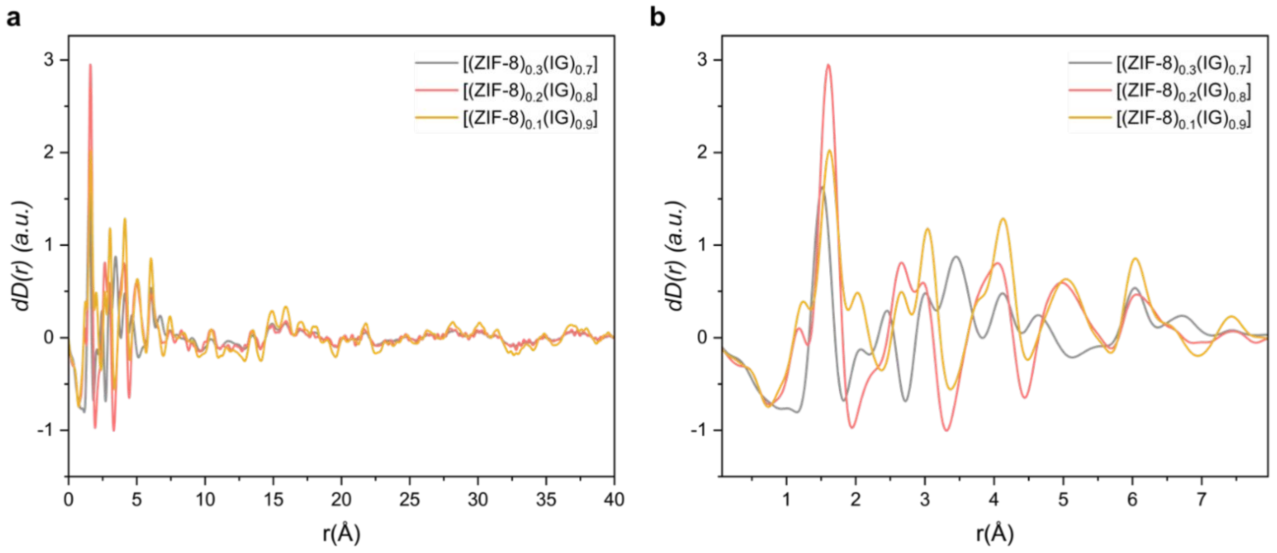

Figure S67. a. Differential pair distribution function *D(r)* of the different MOF-CIGCs [$(ZIF\text{-}8)_{0.3}(IG)_{0.7}$] (grey), [$(ZIF\text{-}8)_{0.2}(IG)_{0.8}$] (red) and [$(ZIF\text{-}8)_{0.1}(IG)_{0.9}$] (yellow). **b**. Zoom-in to show the local structure of the dPDFs. dPDF functions were obtained by the subtracting the physical mixture from the equivalent composite with a normalisation factor to equalise the contributions located at 2.0 Å (Zn-N) for compare between compositions. Two new peaks located at 2.50 and 3.38 Å in [$(ZIF\text{-}8)_{0.3}(IG)_{0.7}$] material may be related with a new correlation Na···N and P···Zn, respectively.

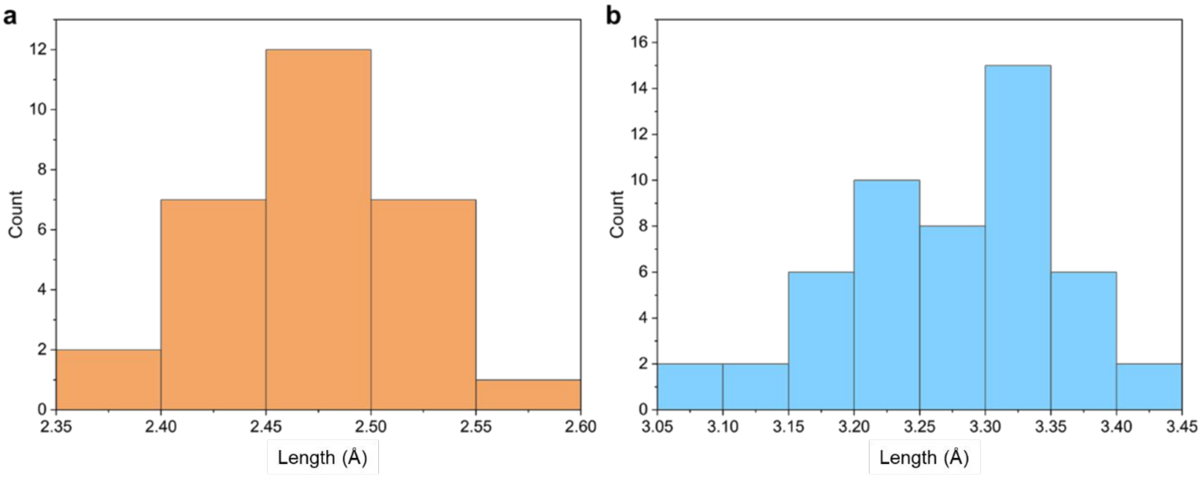

Figure S68. a. Length distribution of atomic distances Na–N for different coordination polymers (CCDC numbers: 143006, 229973, 2065206, 1959297, 1959298, 1576886, 1576880, 286928, 2075203, 2117904, 2107231 and 2091289). **b**. Length distribution of atomic correlations P–O–Zn for different zinc phosphate structures (CCDC numbers: 2310789, 1007095 and 2310787).

12. Principal component analysis

This methodology assembles the data into a correlation or covariance matrix, whit *m* datasets and attempts to describe them in terms of linear combinations of *n* components, satisfying the condition $n<m$. In PCA, these components are constrained to be orthogonal functions and they appear as eigenvalues representing the relative importance of each component (eigenvector).

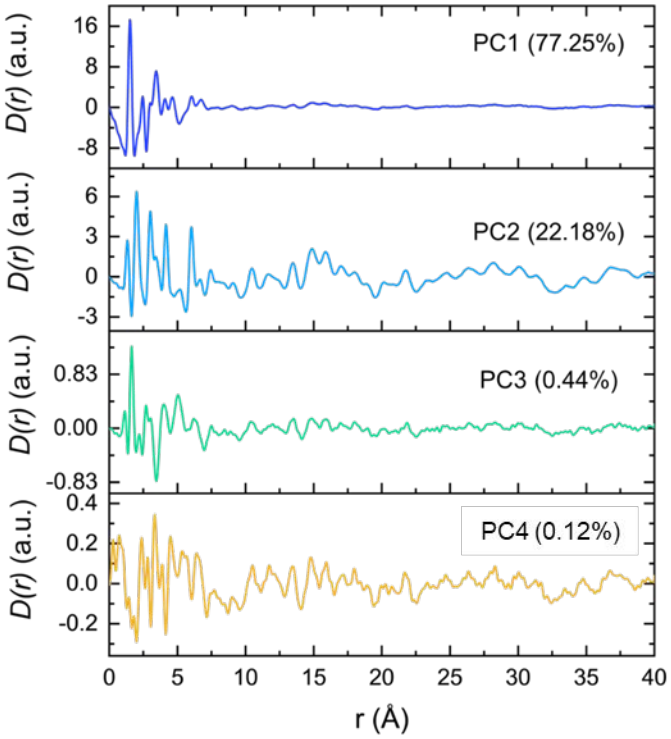

Figure S69. Principal component analysis of the composites with % values of 77.25, 22.18, 0.44, and 0.12% for PC1, PC2, PC3 and PC4, respectively. PC4 was considered to be largely consisting of noise, given the far smaller contribution.

Table S5. Pearson's correlation coefficients

Composites	Glass	$[(ZIF\text{-}8)_{0.1}(IG)_{0.9}]$	$[(ZIF\text{-}8)_{0.2}(IG)_{0.8}]$	$[(ZIF\text{-}8)_{0.3}(IG)_{0.7}]$	ZIF-8
Glass	1	0.97163	0.941	0.88182	-0.09588
$[(ZIF\text{-}8)_{0.1}(IG)_{0.9}]$	0.97163	1	0.98862	0.95153	0.01738
$[(ZIF\text{-}8)_{0.2}(IG)_{0.8}]$	0.941	0.98862	1	0.98627	0.13545
$[(ZIF\text{-}8)_{0.3}(IG)_{0.7}]$	0.88182	0.95153	0.98627	1	0.26074
ZIF-8	-0.09588	0.01738	0.13545	0.26074	1

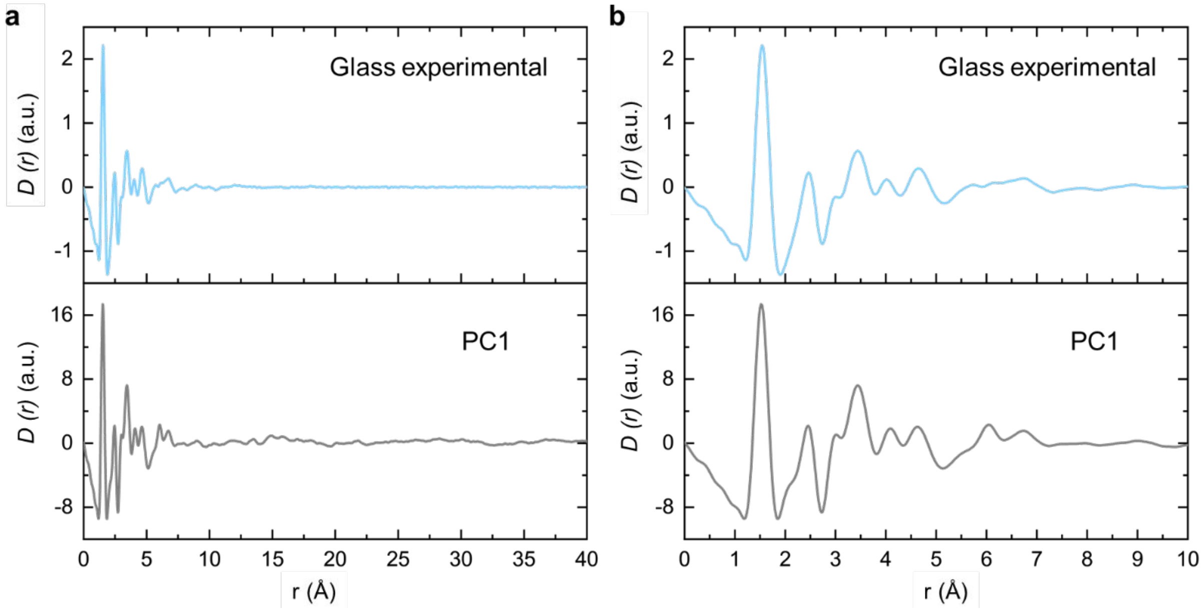

Figure S70. a. Comparison of PC1 *vs* the PDF from pristine inorganic glass. **b**. Comparison of the local structure of PC1 and the inorganic glass PDF.

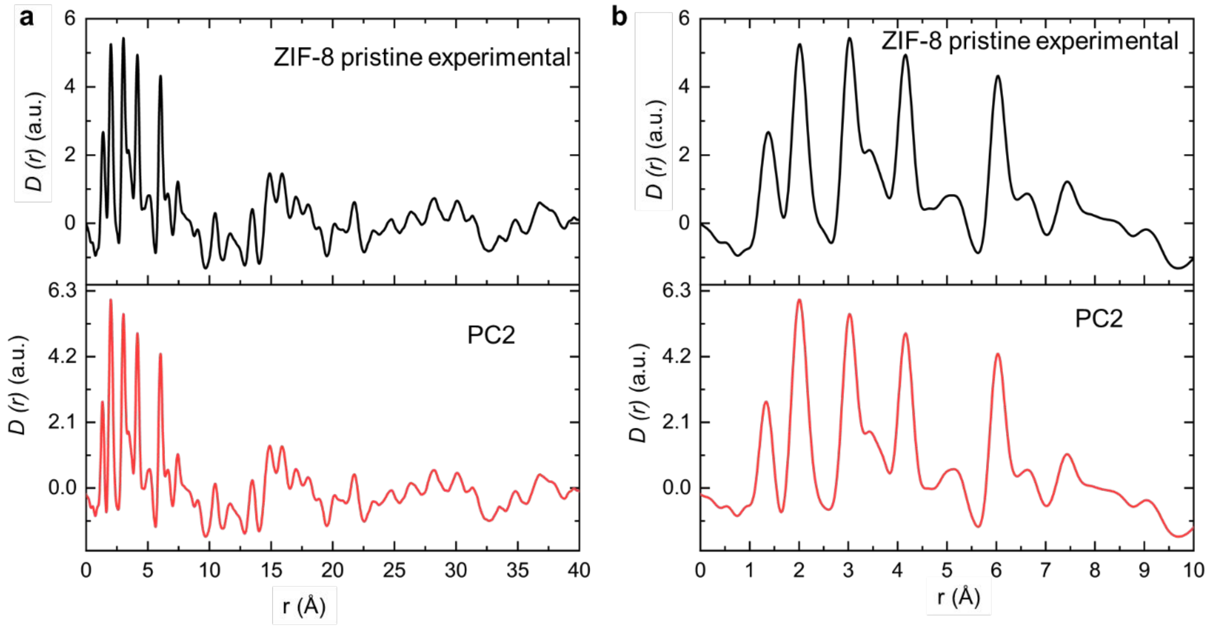

Figure S71. a. Comparison of PC2 *vs* the PDF from pristine ZIF-8. **b**. Comparison of the local form of PC2 and ZIF-8 PDF.

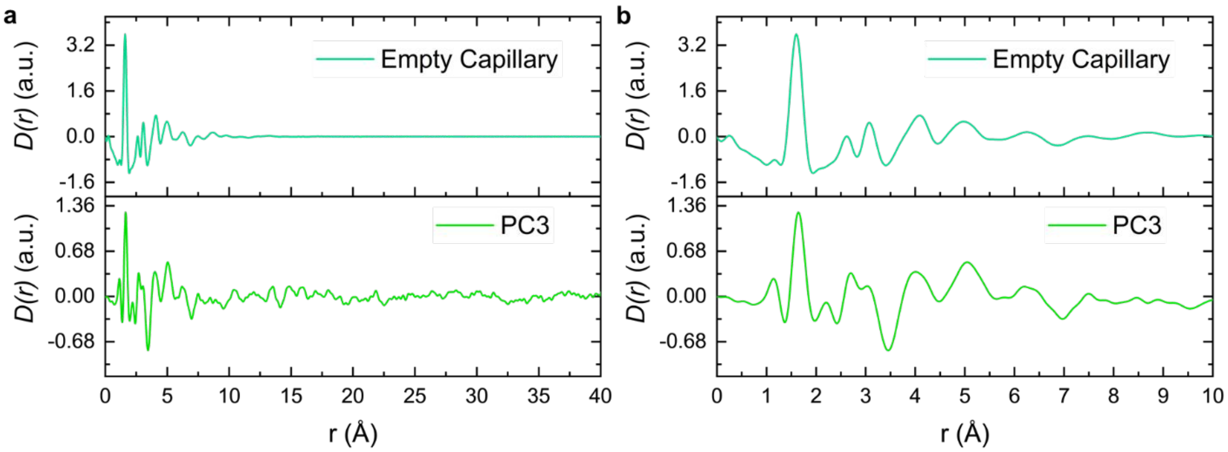

Figure S72. a. Comparison of PC3 *vs* the PDF from the borosilicate capillary. **b**. Comparison of the local form of PC3 and the borosilicate capillary PDF.

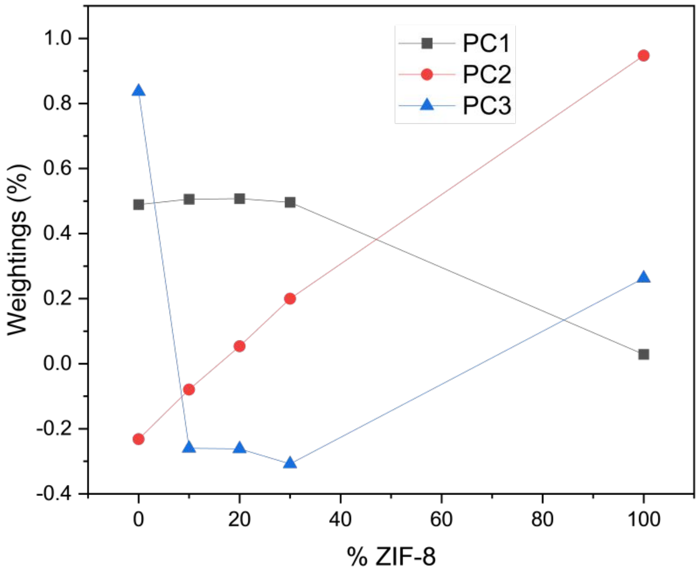

Figure S73. Weighting values of principal components *vs* weight %ZIF-8 in the sample for the compositional series of composites. Weightings of PC3 appear as negative as a result of a flipping process due to PCA.

13. Multiple linear regression

Multiple linear regression analysis of PDFs was carried out using data analysis module of Excel software and applying the equation (1):

$$D_{Composite}(r) = A * D_{IG}(r) + B * D_{ZIF}(r) + C \quad \text{(Eq.1)}$$

Where A is the contribution of the inorganic glass *D(r)*, B the contribution of the ZIF-8 *D(r)* and C is a normalisation constant. In this approach, where the data are only fitted to the two (end member) PDFs, then inevitably some of the suspected third contribution related with the interface interaction might be fitted with the end member PDFs. The end members are ZIF-8 pristine (100%) and pristine inorganic glass (0%).

The refined variables x_1 and x_2 are shown in **Fig. S74** exhibiting a general decreasing/increasing trend with weight% of ZIF-8 in the composite, respectively.

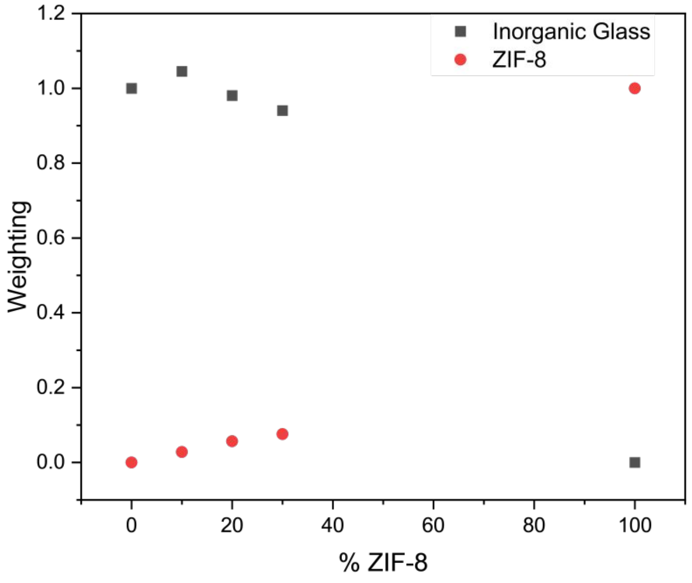

Figure S74. Regression weightings values applying the multilinear regression analysis at different contents of ZIF-8 in the sample.

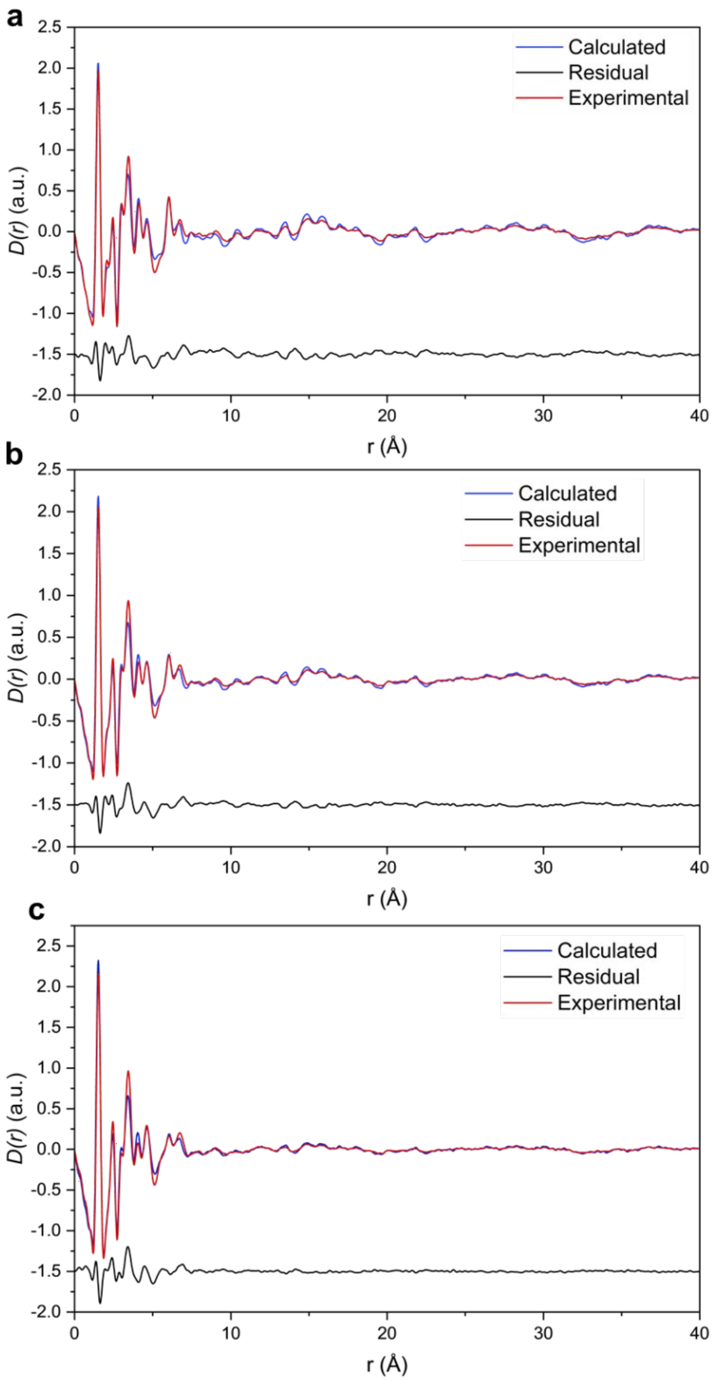

Figure S75. Fits the different composites using multilinear regression. **a**. $[(ZIF\text{-}8)_{0.3}(IG)_{0.7}]$, **b**. $[(ZIF\text{-}8)_{0.2}(IG)_{0.8}]$, **c**. $[(ZIF\text{-}8)_{0.1}(IG)_{0.9}]$.

Table S6. Regression coefficients and associated errors for the composites. A: Inorganic glass contribution, B: ZIF-8 contribution and C: interception.

Regression coefficients	$[(ZIF\text{-}8)_{0.1}(IG)_{0.9}]$	$[(ZIF\text{-}8)_{0.2}(IG)_{0.8}]$	$[(ZIF\text{-}8)_{0.3}(IG)_{0.7}]$
A	1.064(4)	1.013(4)	0.971(4)
B	0.039(1)	0.0698(9)	0.103(1)
C	-0.0001(1)	-0.0012(9)	-0.002(1)
R^2	0.9674	0.9688	0.9631

R^2-value of the end members is 1, due to the perfect fit with one of the components. However, for the intermediate members, we are using a two components model to fit what we believe is a three-component system: ZIF-Inorganic Glass-interface. Therefore, the R-values for intermediate members of the series would be expected to decrease. They also suggest the deviation from the two-component model is greatest for the composite containing a 20% of ZIF-8. This is perhaps the sample that maximizes the interaction between phases.

We can visualize this change in the R-value through the residuals, that are left over after regression. R-values of 1 for end members mean zero residual, and consequently a flat line. Residuals in the intermediate compositions are features that cannot be accounted for using ZIF-8 or the inorganic glass, these represent the minimum difference as some of the interaction PDF features will have been fitted by the ZIF/IG.

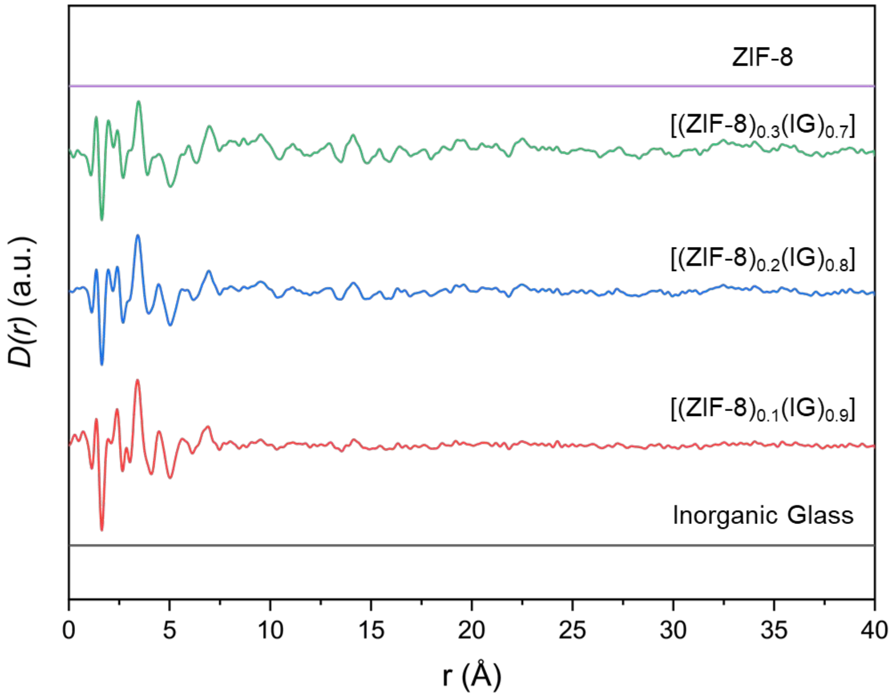

Figure S76. Residuals obtained from the multilinear regression.

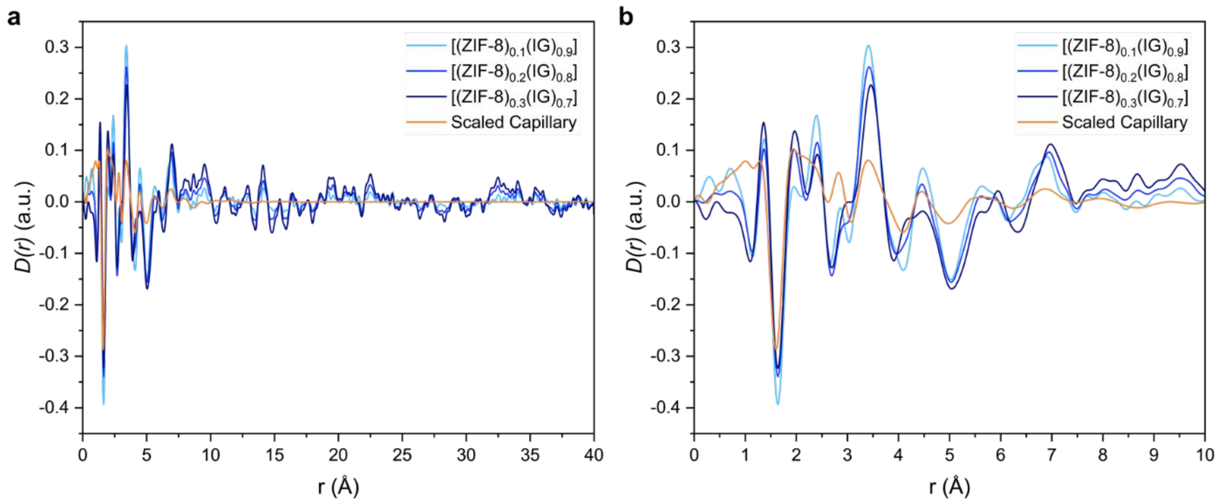

Figure S77. a. Comparison of the residuals from the MLR and the borosilicate capillary scaled by a -0.08 factor. **b**. Zoom-in shows the local structure of the residuals with the scaled borosilicate capillary. Borosilicate capillary was scaled to compare with the residuals obtained from the MLR, due to the contribution of the capillary may affect to the final result in the MLR analysis.

14. Estimation of the interfacial thickness

We really appreciate the referee's suggestion about the estimation of the interface thickness in the composite. This calculation has been now addressed as follows:

The calculation or direct measurement of the thickness of the interface ZIF-8 glass is a very challenging question to answer due to the really small contribution of this interaction compared to the bulk. We attempt to estimate this value from the principal component analysis (PCA) of PDF data and by making a number of significant assumptions that will allow us to calculate an upper bound to the interface thickness. From PCA, we were able to extract all the contributions of the composites, this is: the glass, the ZIF-8 and the interaction at the interface with some contributions of the borosilicate glass capillary. The weightings of these components (PC1, PC2 and PC3) were 77.25%, 22.18% and 0.44%, respectively and correspond to the composite contributions of glass, ZIF-8 and interface.

If we broadly assume (and only for the purposes of this order-of-magnitude calculation) that the relative weights of PC2 and PC3 correspond to the relative scattering contributions of ZIF-8 and the interfacial region, then the interfacial contribution is clearly low compared to both bulk components. Moreover, PC3 also contains residual contributions from the borosilicate glass capillary which are difficult to quantify. We therefore assume that 0.44% is the maximum scattering associated with the interface. To further simplify the calculation, we also make the crude assumptions that the density and scattering strength of the interface region are the same as that of ZIF-8 such that the interface:ZIF-8 scattering ratio is the same as the volume ratio. We also assume that every ZIF-8 particle is a similar size and is surrounded by an interfacial region. Therefore, we can calculate the proportion of the interface from ZIF-8 particles:

$$\%Interface\ Volume\ as\ proportion\ of\ a\ ZIF-8\ particle\ Volume = \frac{\%PC3}{\%PC2+\%PC3} = \frac{0.44}{22.18+0.44} = 0.019$$

All ZIF-8 particles possess a very similar shape (rhombic dodecahedron) with an edge length of 310 nm, see figure S78.

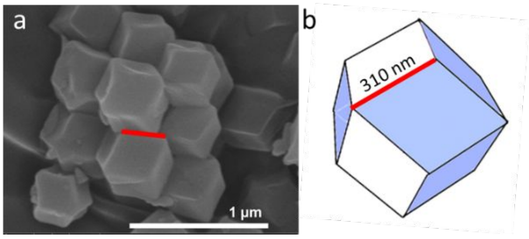

Figure S78. a SEM image of ZIF-8 crystallites. **b**. 3D model of a rhombic dodecahedron similar to ZIF-8 crystallites with a edge length value of 310 nm.

We calculate the volume from the following equation; *e* is the edge length:

$$V_{ZIF-8\,particles} = \frac{15+7\sqrt{5}}{4}e^3$$

and the interface volume would have the following formula:

$$V_{Interface} = \frac{15+7\sqrt{5}}{4}(e+\delta e)^3 - \frac{15+7\sqrt{5}}{4}e^3$$

Substituting the previous obtained value of the % of the interface in ZIF-8 particles and the edge length we obtain:

$$315.89 = 310 + \frac{3\delta e}{e} + \frac{3\delta e^2}{e^2} + \frac{\delta e^3}{e^3}$$

Solving for δe, an approximate value of the maximum interfacial thickness is ≈ 1 nm. This result seems reasonable given the several assumptions used in the mathematical approach describe above.

The obtained features at the PC3 and the residuals from the MLR exhibit very similar features, despite this is a broad estimation. These results are very promising and reliable. However, to improve this results in future, experimental conditions for total scattering data collection must be modified. Firstly, it is likely increment exposure times and increase the flux may result in data with a better resolution. Secondly, increasing the number of variables, that means have more different compositions, to improve the statistics. Finally, would be also desirable to compare the results of PCA, MLR and dPDF with non-negative matrix factorisation (NMF).

Table S7. Estimation of the % of Interface volume relative to the volume of the ZIF-8 particle contribution with different thickness maintaining the same crystal size.

Interface thickness (nm)	% Interface volume relative to the volume of the ZIF-8 particle
0.5	0.007
1.0	0.019
2.0	0.058
5.0	0.290

15. Compression tests

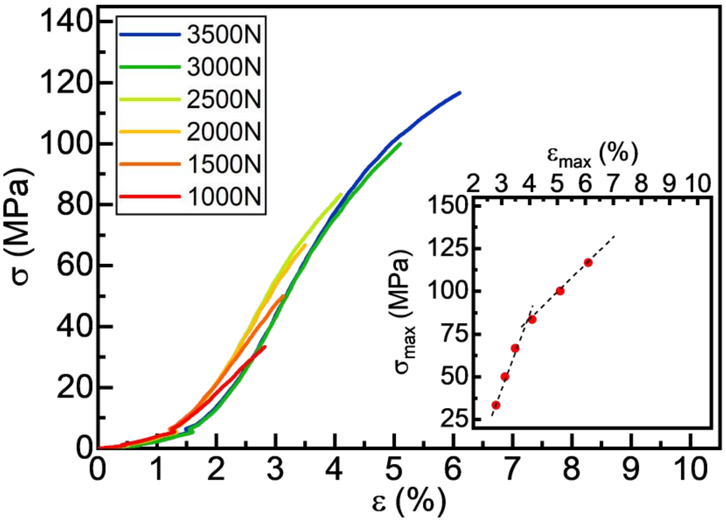

Figure S79. Stress-strain diagram for $[(ZIF\text{-}8)_{0.1}(IG)_{0.9}]$ composite. Pellet dimensions: 6.18 mm x 1.65mm (Area = 29.996 mm^2). The curves reveal densification/particle rearrangement of the pellet under the uniaxial compression until 2000N, as evident from the gradually increasing steepness of the curve with each cycle. Above 2500 N the data revealed cracking of the sample, evident from the generally shifted curves to higher strain (3000 N and 3500 N), which indicates that during the loading to 2500N, initial crack formation occurred. The inset reveals the transition between the regime where pure elastic compression and pellet densification occurs to the regime where defect formation and crack growth are affecting the stress build-up.

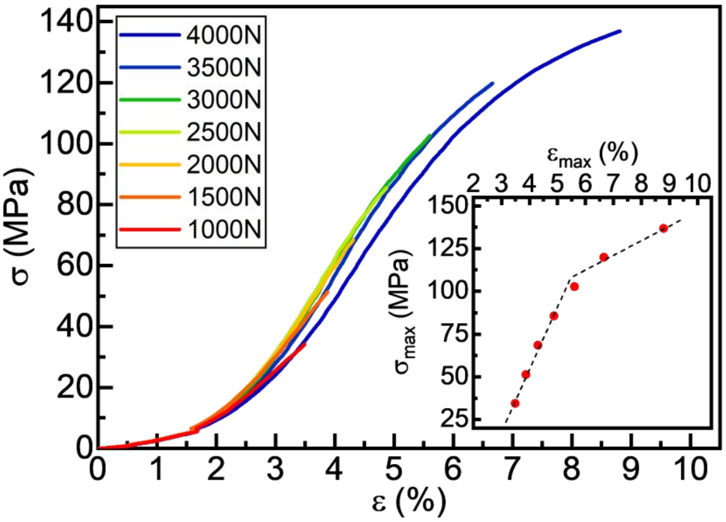

Figure S80. Stress-strain diagram for $[(ZIF\text{-}8)_{0.2}(IG)_{0.8}]$ composite. Pellet dimensions: 6.10 mm x 1.73mm (Area = 29.225 mm^2). The curves reveal densification/particle rearrangement of the pellet under the uniaxial compression until 3000N, as evident from the gradually increasing steepness of the curve with each cycle. Above 3500 N the data revealed cracking of the sample, evident from the generally shifted curves to higher strain (4000 N), which indicates that during the loading to 3500N, initial crack formation occurred. The inset reveals the transition between the regime where pure elastic compression and pellet densification occurs to the regime where defect formation and crack growth are affecting the stress build-up.

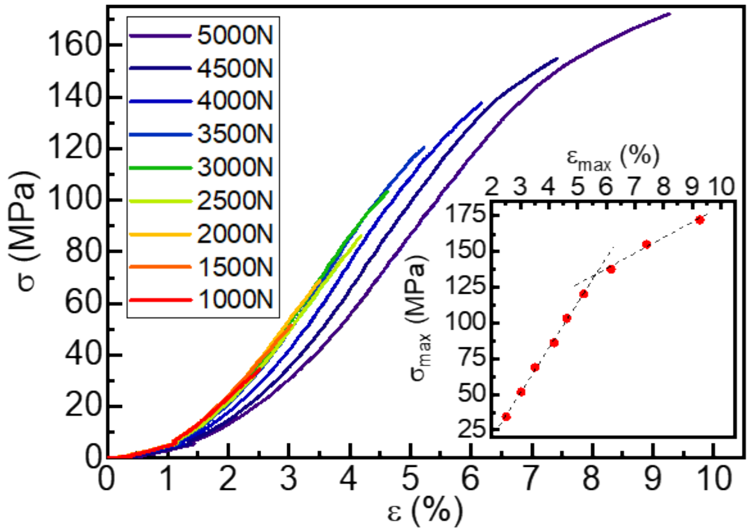

Figure S81. Stress-strain diagram for [(ZIF-8)$_{0.3}$(IG)$_{0.7}$] composite. Pellet dimensions: 6.08 mm x 1.77 mm (Area = 29.033 mm^2). The curves reveal densification/particle rearrangement of the pellet under the uniaxial compression until 3000N, as evident from the gradually increasing steepness of the curve with each cycle. Above 3500 N the data revealed cracking of the sample, evident from the generally shifted curves to higher strain (4000 N and 3500 N), which indicates that during the loading to 3500N, initial crack formation occurred. The inset reveals the transition between the regime where pure elastic compression and pellet densification occurs to the regime where defect formation and crack growth are affecting the stress build-up.

Table S8. Threshold values for stress (σ) and strain (ε).

**Sample**	**ε_{max} (%)**	**σ_{max} (MPa)**
[(ZIF-8)$_{0.1}$(IG)$_{0.9}$]	3.767	81
[(ZIF-8)$_{0.2}$(IG)$_{0.8}$]	5.534	109
[(ZIF-8)$_{0.3}$(IG)$_{0.7}$]	5.613	133

16. Carbon dioxide isotherms

Table S9. STP maximum amount absorbed values obtained from CO_2 adsorption isotherms. Composites are noted as $[(ZIF\text{-}8)_x(IG)_{1-x}]$ and physical mixtures as $(ZIF\text{-}8)_x/(IG)_{1-x,}$ where x is the proportion in weight of ZIF-8.

Sample	STP (cm^3/g)
ZIF-8 0.22 GPa	39.44
ZIF-8 pristine	46.66
$50Na_2O\text{-}50P_2O_5$	1.06
$[(ZIF\text{-}8)_{0.1}(IG)_{0.9}]$	4.95
$[(ZIF\text{-}8)_{0.2}(IG)_{0.8}]$	8.08
$[(ZIF\text{-}8)_{0.3}(IG)_{0.7}]$	10.76
$(ZIF\text{-}8)_{0.1}/(IG)_{0.9}$	4.90
$(ZIF\text{-}8)_{0.2}/(IG)_{0.8}$	8.65
$(ZIF\text{-}8)_{0.3}/(IG)_{0.7}$	12.93

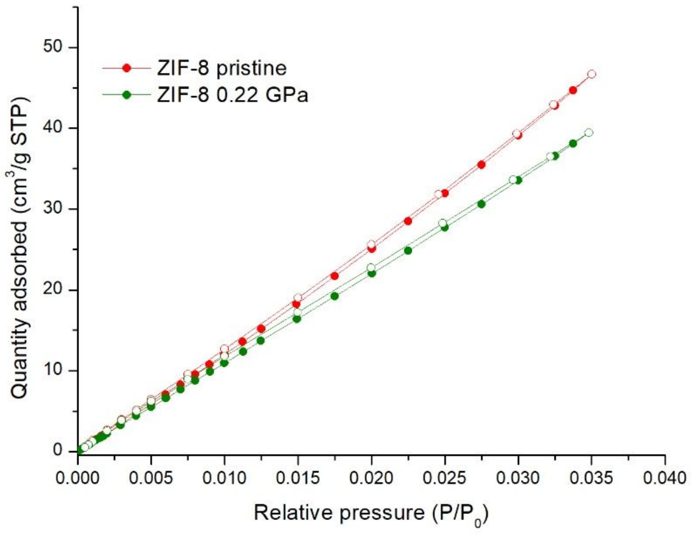

Figure S82. CO_2 adsorption isotherms for ZIF-8 pristine (red) and ZIF-8 after pelletisation at 0.22 GPa (green) showing a similar response. Desorption isotherms where depicted with the same colours than their corresponding adsorption isotherms with the circle fill colour in white.

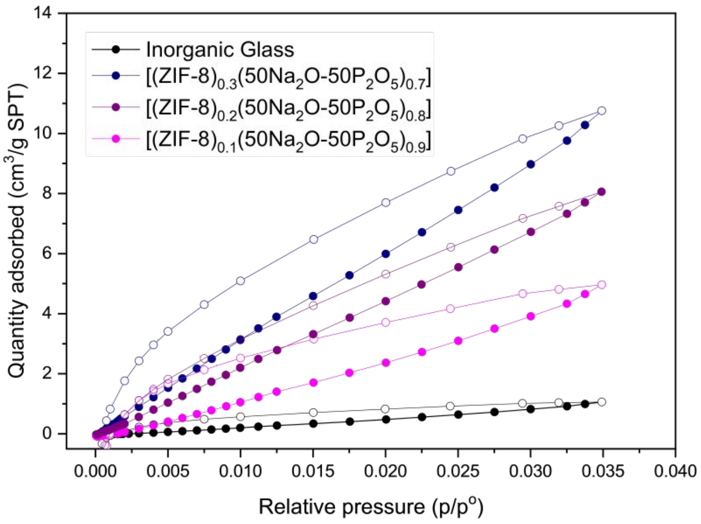

Figure S83. CO_2 adsorption isotherms for inorganic glass (black), [$(ZIF\text{-}8)_{0.3}(IG)_{0.7}$] (navy), [$(ZIF\text{-}8)_{0.2}(IG)_{0.8}$] (purple) and [$(ZIF\text{-}8)_{0.1}(IG)_{0.9}$] (pink) composites. Desorption isotherms where depicted with the same colours than their corresponding adsorption isotherms with the circle fill colour in white.

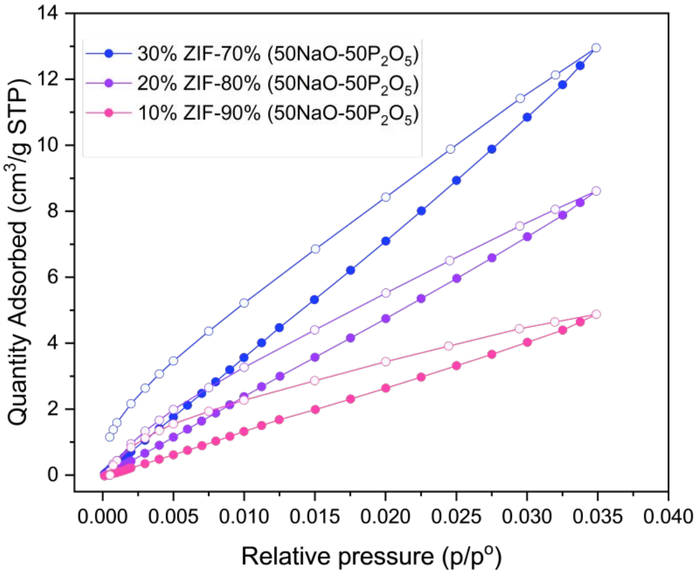

Figure S84. CO_2 adsorption isotherms for $(ZIF\text{-}8)_{0.3}/(IG)_{0.7}$ (blue), $(ZIF\text{-}8)_{0.2}/(IG)_{0.8}$ (light purple) and $(ZIF\text{-}8)_{0.1}/(IG)_{0.9}$ (light pink) physical mixtures. Desorption isotherms where depicted with the same colours than their corresponding adsorption isotherms with the circle fill colour in white.

17. Stability test

For the stability test a 20 mg piece of composite was left exposed in air with 66% relative humidity. PXRD of the samples were collected before and after to expose the sample to the air.

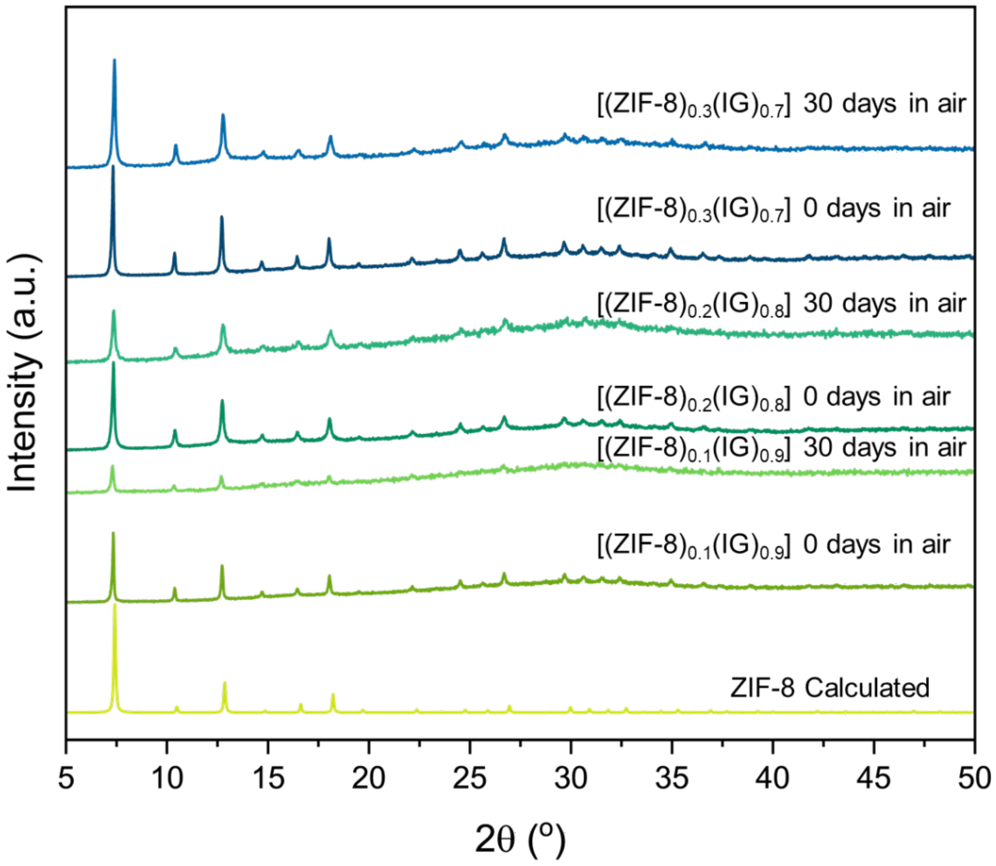

Figure S85. PXRD of the composites before and after being exposed in air for 30 days.

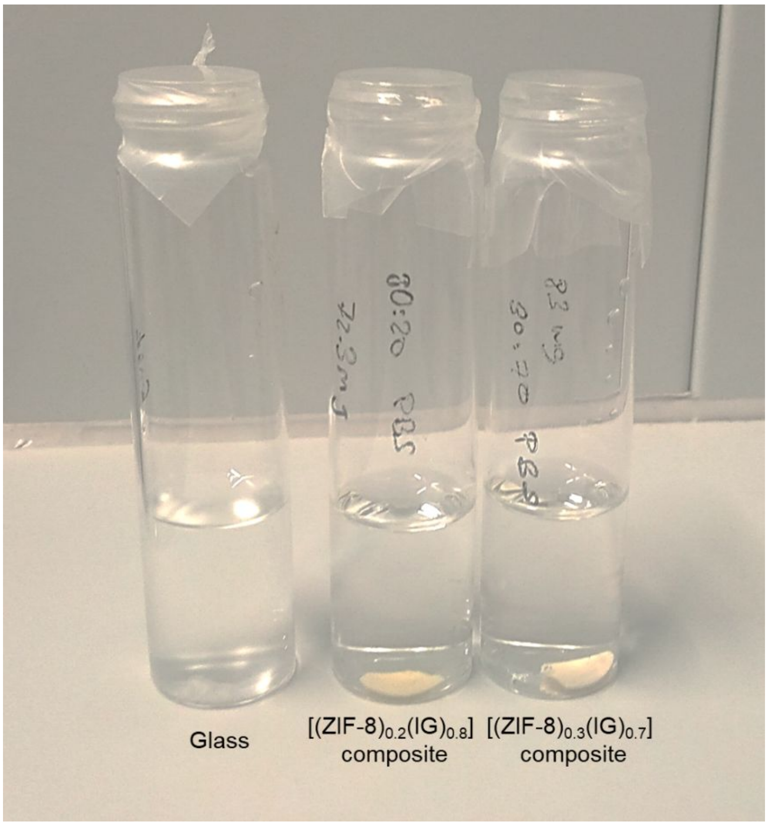

Figure S86. Image of 70 mg of the glass and composites after being exposed into phosphate buffer saline (PBS) solution for 10 days.

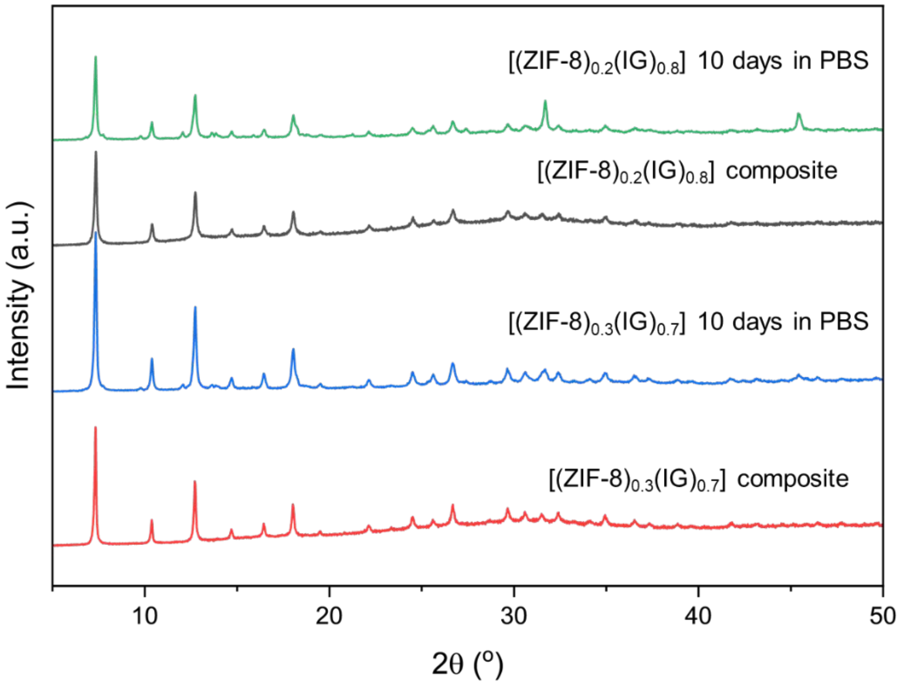

Figure S87. PXRD of the composites before and after being exposed into phosphate buffer saline (PBS) solution for 10 days.

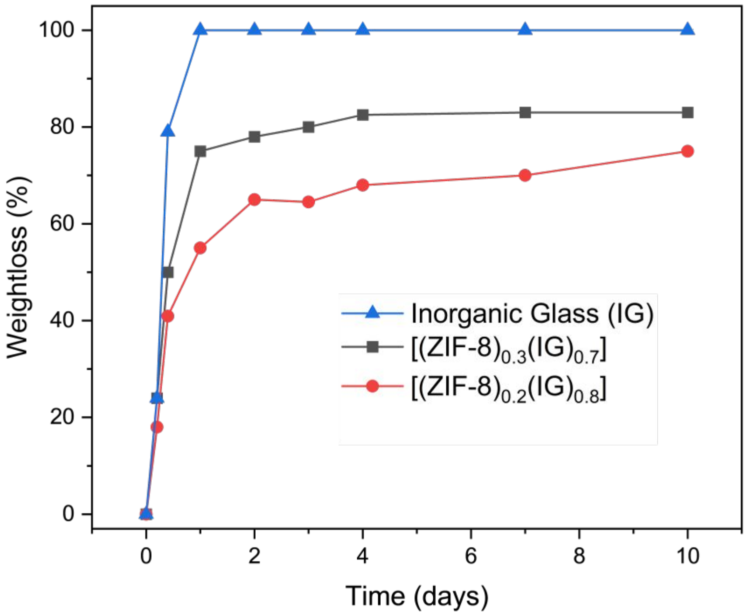

Figure S88. Kinetic curves for the weightloss of the composite or IG within the time when the material is immersed in a phosphate buffer saline (pH = 7.4) solution.

Stability test in multiple polar, namely: methanol, ethanol, and acetone. A piece of the [(ZIF-8)$_{0.3}$(IG)$_{0.7}$] composite was immersed in the correspondent solvent for 48 hours. Difference of weight and PXRD patterns before and after the immersion were registered.

Table S10. Weightloss of the composite after being immersed for 48 hours in the selected solvent. The purity of the employed solvents was 99%.

Solvent	Methanol	Ethanol	Acetone
Weight before immersion (mg)	26.2	27.8	31.5
Weight after immersion (mg)	25.5	25.9	30.6
%loss in 48 h	2.67	6.83	2.86

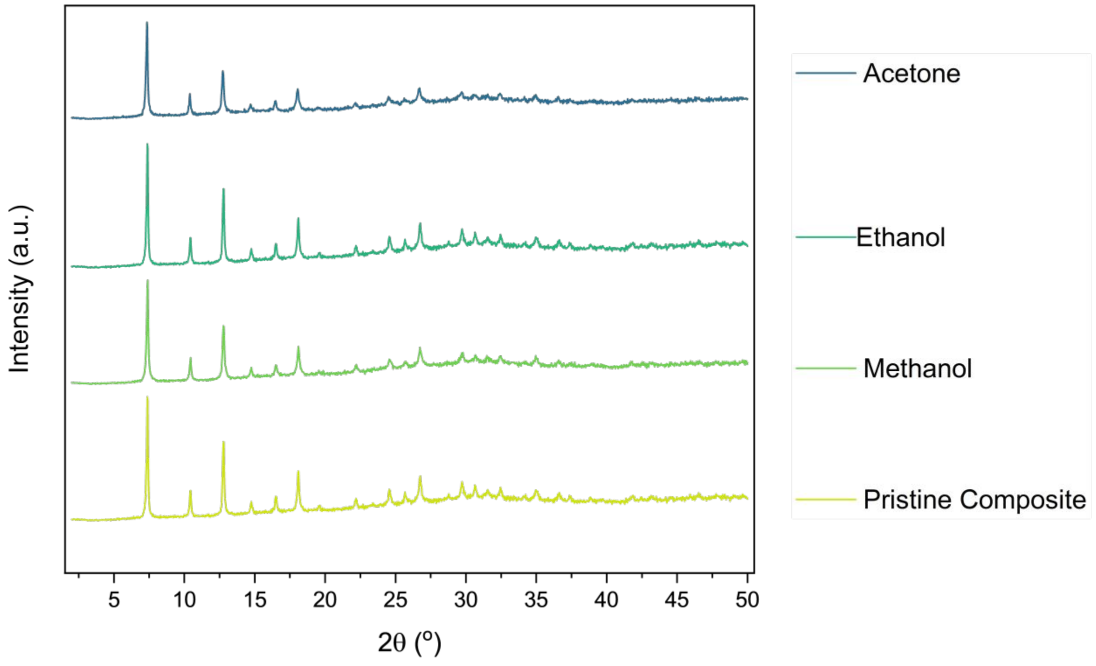

Figure S89. PXRD patterns of the [(ZIF-8)$_{0.3}$(IG)$_{0.7}$] composite after being immersed in 10 mL of the multiple polar solvents.

18. References

(1) Avci, C.; Ariñez-Soriano, J.; Carné-Sánchez, A.; Guillerm, V.; Carbonell, C.; Imaz, I.; Maspoch, D. Post-Synthetic Anisotropic Wet-Chemical Etching of Colloidal Sodalite ZIF Crystals. *Angew. Chemie* **2015**, *127* (48), 14625–14629.

(2) Morris, W.; Stevens, C. J.; Taylor, R. E.; Dybowski, C.; Yaghi, O. M.; Garcia-Garibay, M. A. NMR and X-Ray Study Revealing the Rigidity of Zeolitic Imidazolate Frameworks. *J. Phys. Chem. C* **2012**, *116* (24), 13307–13312.

(3) Poletto Rodrigues, B.; Limbach, R.; Buzatto de Souza, G.; Ebendorff-Heidepriem, H.; Wondraczek, L. Correlation Between Ionic Mobility and Plastic Flow Events in NaPO3-NaCl-Na2SO4 Glasses . *Frontiers in Materials* . 2019.

(4) Longley, L.; Calahoo, C.; Southern, T. J. F.; Evans, R. C.; Wondraczek, L.; Bennett, T. D. The Reactivity of an Inorganic Glass Melt with ZIF-8. *Dalt. Trans.* **2021**, *50* (10), 3529–3535.

(5) Farrow, C. L.; Juhas, P.; Liu, J. W.; Bryndin, D.; Božin, E. S.; Bloch, J.; Proffen, T.; Billinge, S. J. L. PDFfit2 and PDFgui: Computer Programs for Studying Nanostructure in Crystals. *J. Phys. Condens. Matter* **2007**, *19* (33), 335219.

(6) Hu, Y.; Kazemian, H.; Rohani, S.; Huang, Y.; Song, Y. In Situ High Pressure Study of ZIF-8 by FTIR Spectroscopy. *Chem. Commun.* **2011**, *47* (47), 12694–12696.

(7) Ordoñez, M. J. C.; Balkus, K. J.; Ferraris, J. P.; Musselman, I. H. Molecular Sieving Realized with ZIF-8/Matrimid® Mixed-Matrix Membranes. *J. Memb. Sci.* **2010**, *361* (1), 28–37.

(8) Narayanan, M. K.; Shashikala, H. D.; Manjaiah, M. Statistical Optimization of Melt-Quenching Process Parameters for Multiple Properties of Ternary Barium Phosphate Glasses. *Mater. Chem. Phys.* **2015**, *152*, 127–134.

(9) Wenslow, R. M.; Mueller, K. T. Structural Details of Aqueous Attack on a Phosphate Glass by 1H/31P Cross-Polarization NMR. *J. Phys. Chem. B* **1998**, *102* (45), 9033–9038.
